# Synthesis of some quinazolinones inspired from the natural alkaloid L*-*norephedrine as EGFR inhibitors and radiosensitizers

**DOI:** 10.1080/14756366.2020.1854243

**Published:** 2020-12-28

**Authors:** Mostafa M. Ghorab, Maged S. Abdel-Kader, Ali S. Alqahtani, Aiten M. Soliman

**Affiliations:** a Department of Drug Radiation Research, National Center for Radiation Research and Technology (NCRRT), Egyptian Atomic Energy Authority (EAEA), Cairo, Egypt; b Department of Pharmacognosy, College of Pharmacy, Prince Sattam Bin Abdulaziz University, Al-Kharj, Saudi Arabia; c Department of Pharmacognosy, College of Pharmacy, Alexandria University, Alexandria, Egypt; d Department of Pharmacognosy, College of Pharmacy, King Saud University, Riyadh, Saudi Arabia; e Medicinal, Aromatic and Poisonous Plants Research Center (MAPPRC), College of Pharmacy, King Saud University, Riyadh, Saudi Arabia

**Keywords:** Quinazolinone, cytotoxicity, EGFR, anticancer, docking

## Abstract

A set of quinazolinones synthesized by the aid of L-norephedrine was assembled to generate novel analogues as potential anticancer and radiosensitizing agents. The new compounds were evaluated for their cytotoxic activity against MDA-MB-231, MCF-7, HepG-2, HCT-116 cancer cell lines and EGFR inhibitory activity. The most active compounds **5** and **6** were screened against MCF-10A normal cell line and displayed lower toxic effects. They proved their relative safety with high selectivity towards MDA-MB-231 breast cancer cell line. Measurement of the radiosensitizing activity for **5** and **6** revealed that they could sensitize the tumour cells after being exposed to a single dose of 8 Gy gamma radiation. Compound **5** was able to induce apoptosis and arrest the cell cycle at the G2-M phase. Molecular docking of **5** and **6** in the active site of EGFR was performed to gain insight into the binding interactions with the key amino acids.

## Graphical Abstract

**Figure:**
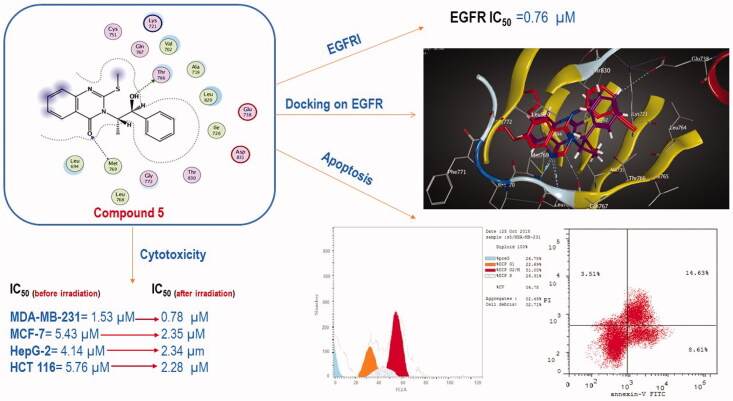

## 1. Introduction

Cancer is characterized by the disturbance of normal cellular processes required for cell growth, division and differentiation^1–3^. Surgery, radiotherapy and chemotherapy, including immunotherapy, targeted and combined therapy, are different strategies advocated for cancer treatment^4–6^.

Protein kinases (PKs) play a pivotal role in cell proliferation by controlling signal transduction through the phosphorylation of different amino acid residues, namely tyrosine, threonine and serine^7^. Tyrosine kinases (TKs) are divided into receptor tyrosine kinases (RTKs) and non-receptor tyrosine kinases (NRTKs) in human genome. RTKs are vital components of cellular signaling pathways that are active during embryonic development and adult homeostasis. Due to their role as growth factor receptors, many RTKs have been involved in the onset or progression of various cancers, either by mutations or receptor/ligand overexpression; thus, they are considered attractive candidates for therapeutic intervention^7^^,^^8^. An example of RTK family members is epidermal growth factor receptor (EGFR). EGFR is a member of the ErbB receptor family and plays an essential role in cell signaling. Signaling is initiated by binding ligands to the extracellular domain of the EGFR, activating kinases and promoting cancer cell survival, invasiveness and drug resistance^9^^,^^10^. EGFR has a critical role in regulating several cellular functions such as cell growth, proliferation, differentiation and apoptosis, leading to the development of several types of solid tumors^11^. EGFR (HER-1) and ERB-B2 (HER-2) are characterized in solid tumors as breast, ovary, lung and others. The inhibition of EGFR is classified as targeted therapy as it aims at the differences between cancer and normal cells and is characterized by its high selectivity and lowered side effects.

**Figure 1.**
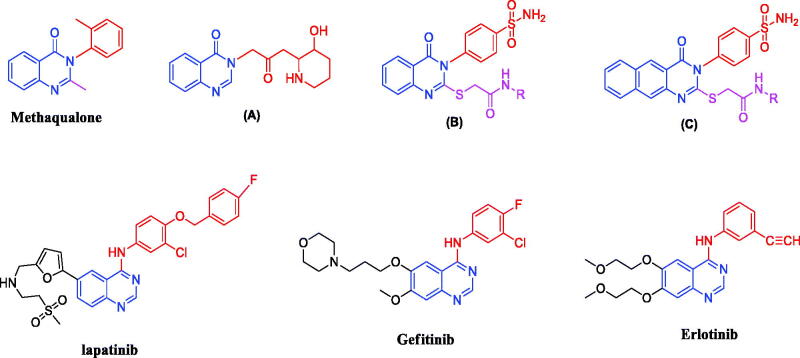
Quinazoline-based scaffolds used to design our target compounds.

Quinazolines are fused heterocyclic ring systems known for their variable biological activity^12–15^. They are well known for their inhibitory activity towards various protein kinase enzymes and their anticancer activity^16^. For example, lapatinib, a dual reversible EGFR and HER2 inhibitor. Also, gefitinib and erlotinib are reversible EGFR inhibitors; they are examples of FDA approved small molecules TK inhibitors^17^. Methaqualone, a potent hypnotic, was considered as an important landmark in synthetic anticonvulsants^18^. The 3-[β-keto-gamma-(3-hydroxy-2-piperidyl)-propyl]-4-quinazolone (**A**) was the first isolated natural quinazolinone alkaloid known by its antimalarial activity^19^. The quinazolinone derivatives (**B**) and benzo[g]quinazolinone (**C**) were reported to possess potent EGFR and HER2 inhibitory activity^20^^,^^21^ (Figure 1). On the other hand, the Ephedra alkaloid, Norephedrine (NE) is a stereoisomer of phenylpropanolamine that is naturally occurring sympathomimetic^22^. Investigation revealed that long-term use of NE caused severe side effects, including fatality^23^. In addition to medicinal use, the properties of this alkaloid have attracted considerable attention in natural product chemistry field that leads to its use as a starting material in the preparation of chiral ligands for asymmetric catalytic synthesis^24^^,^^25^.

In continuation of our studies aiming to find new leads with potential anticancer activities, various substituted quinazolinones have been designed to accommodate different electronic natures as heterocycles representing the primary scaffold in many cytotoxic agents hoping to develop potent and safe anticancer agents and EGFR inhibitors. All the synthesized compounds were screened against MDA-MB-231, MCF-7, HepG-2, HCT-116 cancer cell lines and the most potent compounds were evaluated against MCF-10A normal cells to determine the selectivity of the compounds on the different cell lines. Also, the *in vitro* EGFR inhibitory activity of the compounds was measured. The effect of the most potent compounds on cell cycle progression and the radiosensitizing activity were evaluated. Docking studies were carried out to confirm the possible mode of action of the promising compounds.

## 2. Materials and methods

### 2.1. Chemistry

Melting points were determined uncorrected by a Gallen Kamp melting point apparatus (Sanyo Gallen Kamp, UK). Precoated silica gel plates (*Kieselgel* 0.25 mm, 60 F254, Merck, Germany) were used for TLC with solvent system of chloroform/methanol (8:2), spots were detected by UV light. IR spectra (KBr discs) were recorded using FT-IR spectrophotometer (Perkin Elmer, USA). ^1^H, ^13 ^C NMR and 2D NMR experiments were scanned on an NMR spectrophotometer (Bruker AXS Inc., Switzerland), operating at 500 MHz for ^1^H and 125.76 MHz for ^13 ^C. Chemical shifts are expressed in *δ*-values (ppm) relative to TMS as an internal standard, using DMSO-d_6_ and CDCl_3_ as solvents. EIMS were measured using Shimadzu-GC/MS. Elemental analyses were performed on a model 2400 CHNSO analyser (Perkin Elmer, USA). All the values were within ±0.4% of the theoretical values. The X-ray data were collected at *T* = 298 K on Enraf Nonius 590 Kappa CCD single crystal diffractometer equipped with graphite monochromated Mo Kα (*λ*=0.71073 Å) radiation using ψ–ω scan technique. All reagents used were of AR grade.

#### 2.1.1. Methyl 2-(3-(1-hydroxy-1-phenylpropan-2-yl)thioureido)benzoate (3) & 3-(1-hydroxy-1-phenylpropan-2-yl)-2-thioxo-2,3-dihydroquinazolin-4(1H)-one (4)

Methyl 2-isothiocyanatobenzoate **1** (0.193 g, 0.001 mol) was allowed to react with L-norephedrine (2-amino-1-phenylpropan-1-ol) (0.151 g, 0.001 mol) **2** in NMR tube in CDCl_3_ and measured immediately for ^1^H and ^13 ^C NMR to give **3**. When the reaction was carried out in the presence of chloroform containing a catalytic amount of triethylamine the reaction gave **4** instead of **3** at room temperature. The product **4** was crystallized from ethanol. Derivative **3** was rapidly converted to **4** at room temperature.

**3*:*** ^1^H NMR (500 MHz, CDCl_3_): 0.99 (d, *J* = 6.0 Hz, 3H, CH_3_ of L-norephedrine), 2.87 (bs, 1H, NH), 3.83 (s, 3H, O–CH_3_), 4.81 (bs, 1H, N–CH), 5.13 (bs, 1H, O–CH), 6.64 (d, *J* = 6.8 Hz, 1H, OH), 7.05–7.93 (m, 9 aromatic), 10.31 (bs, 1H, NH). ^13^C NMR (126 MHz, CDCl_3_): 13.30 (CH_3_), 55.76 (O–CH_3_), 75.20 (N–CH), 77.33 (O–CH), 118.80, 122.74, 123.69, 125.06, 126.92 (2), 128.35 (2), 131.44, 133.31, 140.65 (2), 167.96, 179.68. MS *m*/*z* (%): 344 (M^+^) (1.96), 179 (100).

**4*:*** Yield, 83%; m.p. 80.3 °C. IR (KBr, cm^−1^): 3455 (OH), 3244 (NH), 3088 (arom.), 2970, 2865 (aliph.), 1691 (CO), 1277 (CS). ^1^H NMR (500 MHz, DMSO-d_6_): *δ* 1.60 (d, *J* = 6.0 Hz, 3H, CH_3_ of L-norephedrine), 5.33 (bs, 1H, N–CH), 5.66 (bs, 1H, O–CH), 6.11 (bs, 1H, NH), 7.06–7.30 (m, 7 aromatic), 7.63 (t, *J* = 6.0 Hz, 1H), 7.88 (d, *J* = 7.0 Hz, 1H), 12.30 (s, 1H, OH). ^13 ^C NMR (126 MHz, DMSO-d_6_): *δ* 13.93 (CH_3_), 61.15 (N–CH), 73.84 (O–CH), 115.20, 116.04, 124.39, 126.74 (2), 126.87, 127.41, 128.12 (2), 135.33, 138.51, 142.37, 159.57, 176.01. MS *m*/*z* (%): 312 (M^+^) (10), 78 (100). Anal. Calcd. For C_17_H_16_N_2_O_2_S (312): C, 65.36; H, 5.16; N, 8.97. Found: C, 65.57; H, 5.41; N, 9.19.

#### 2.1.2. 3-(1-Hydroxy-1-phenylpropan-2-yl)-2-(methylthio)quinazolin-4(3H)-one (5)

A mixture of **4** (0.312 g, 0.001 mol) and methyl iodide (0.141 g, 0.001 mol) in dry acetone (30 mL) containing K_2_CO_3_ was refluxed for 12 h. The obtained solid was crystallized from ethanol to give **5**.

**5:** Yield, 78%; m.p. 121.5 °C. IR (KBr, cm^−1^): 3405 (OH), 3055 (arom.), 2966, 2871 (aliph.), 1683 (CO), 1612 (CN). ^1^H NMR (500 MHz, DMSO-d_6_): *δ* 1.71 (d, *J* = 6.5 Hz, 3H, CH_3_ of L-norephedrine), 2.47 (s, 3H, S–CH_3_), 4.45 (bt, 1H, N–CH), 5.57 (bs, 1H, O–CH), 5.87 (bs, 1H, OH), 7.10–7.38 (m, 7 aromatic), 7.69 (t, *J* = 6.5 Hz, 1H), 8.09 (d, *J* = 7.0 Hz, 1H). ^13^C NMR (126 MHz, DMSO-d_6_): *δ* 14.62 (CH_3_), 15.37 (S–CH_3_), 61.98 (N–CH), 72.80 (O–CH), 119.34, 125.51, 125.76, 126.07, 126.36 (2), 127.42, 127.58 (2), 134.55, 142.16, 146.04, 157.15, 161.09. MS *m*/*z* (%): 296 (M^+^-2CH_3_) (12), 180 (100). Anal. Calcd. For C_18_H_18_N_2_O_2_S (326): C, 66.23; H, 5.56; N, 8.58 Found: C, 66.49; H, 5.88; N, 8.87.

#### 2.1.3. 3-Amino-2-thioxo-2,3-dihydroquinazolin-4(1H)-one (6)

The method for the synthesis of compound **6** was reported by El-Hiti et al^26^.

**6:** Yield, 89%; m.p. 261.2 °C. IR (KBr, cm^−1^): 3356, 3280, 3176 (NH_2_, NH), 3096 (arom.), 1696 (CO), 1283 (CS). ^1^H NMR (500 MHz, DMSO-d_6_): *δ* 6.39 (s, 2H, NH_2_), 7.37 (t, *J* = 7.0, 1H), 7.42 (d, *J* = 7.3 Hz, 1H), 7.75 (t, *J* = 7.0, 1H), 7.99 (t, *J* = 7.0, 1H), 12.30 (s, 1H, NH). ^13^C NMR (126 MHz, DMSO-d_6_): *δ* 114.70, 115.78, 124.39, 126.67, 134.85, 138.21, 155.41, 169.27. MS *m*/*z* (%): 193 (M^+^) (95), 162 (100). Anal. Calcd. For C_8_H_7_N_3_OS (193): C, 49.73; H, 3.65; N, 21.75. Found: 49.48; H, 3.31; N, 21.48.

#### 2.1.4. 3-Amino-2-(methylthio)quinazolin-4(3H)-one (7)

A mixture of **6** (0.193 g, 0.001 mol) and methyl iodide (0.141 g, 0.001 mol) was refluxed in dry acetone containing K_2_CO_3_ for 12 h. The reaction mixture was filtered and crystallized from ethanol to give **7**.

**7:** Yield, 81%; m.p. 178.9 °C. IR (KBr, cm^−1^): 3431, 3257 (NH_2_), 3100 (arom.), 2919, 2866 (aliph.), 1689 (CO), 1618 (C=N). ^1^H NMR (500 MHz, DMSO-d_6_): *δ* 2.44 (s, 3H, S–CH_3_), 5.77 (s, 2H, NH_2_), 7.41–8.07 (m, 4H, Ar–H). ^13^C NMR (126 MHz, DMSO-d_6_): *δ* 14.09, 118.75, 125.29, 125.97, 126.07, 134.27, 147.03, 160.49, 160.94. MS *m*/*z* (%): 207 (M^+^) (26), 58 (100). Anal. Calcd. For C_9_H_9_N_3_OS (207): C, 52.16; H, 4.38; N, 20.27. Found: C, 52.02; H, 4.16; N, 20.01.

#### 2.1.5. 3-Methyl-2-phenyl-2H-thiazolo[2,3-b]quinazolin-5(3H)-one (8)

Compound **8** was reported by Ghorab et al^27^.

#### 2.1.6. 3-Methyl-2-phenyl-3,4-dihydro-[1,3,4]oxadiazino[2,3-b]quinazolin-6(2H)-one (9), 3-methyl-2-phenyl-3,4-dihydro-[1,3,4]thiadiazino[2,3-b]quinazolin-6(2H)-one (10) and 3-amino-2-(1-hydroxy-1-phenylpropan-2-ylamino)quinazolin-4(3H)-one (11)

To a solution of **1** (0.193 g, 0.001 mol) in ethanol (25 mL) with **2** (0.151 g, 0.001 mol), hydrazine hydrate (0.05 g, 0.001 mol) was added and refluxed for 20 h. The progress of the reaction was monitored by TLC that indicates the presence of three products. The mixture was filtered to give three compounds **9**, **10** and **11**. The mixture was separated by silica gel column chromatography (45 × 2 i.d. cm, 30 gm) eluting with chloroform, followed by chloroform/methanol mixtures in a gradient system. Fractions 4–7 eluted with chloroform afforded **9** (103 mg) after crystallization from methanol. Fractions 10–12 eluted with chloroform afforded **10** (68 mg) after crystallization from methanol. Fractions 21– 24 eluted with 5% methanol in chloroform afforded **11** (71 mg) after crystallization from methanol.

**9**: Yield, 35%; m.p. 147.5 °C. IR (KBr, cm^−1^): 3149 (NH), 3098 (arom.), 2946, 2907 (aliph.), 1685 (CO), 1602 (CN). ^1^H NMR (500 MHz, DMSO-d_6_): *δ* 0.95 (d, *J* = 6.5 Hz, 3H, CH_3_ of L-norephedrine), 5.18 (t, *J* = 7.0 Hz, 1H, N–CH), 6.18 (d, *J* = 8.0 Hz, 1H, O–CH), 7.37–7.78 (9 aromatic), 8.01 (d, 1H, NH). ^13^C NMR (126 MHz, DMSO-d_6_): *δ* 13.96 (CH_3_), 53.96 (N–CH), 81.86 (O–CH), 118.83, 124.48, 125.84, 126.14, 126.32 (2), 128.52 (2), 128.69, 133.66, 134.72, 148.76, 154.84, 159.86. MS *m*/*z* (%): 293 (M^+^) (1.95), 64 (100). Anal. Calcd. For C_17_H_15_N_3_O_2_ (293): C, 69.61; H, 5.15; N, 14.33. Found: C, 69.40; H, 4.86; N, 14.05.

**10**: Yield, 22%; m.p. 219.9 °C. IR (KBr, cm^−1^): 3210 (NH), 3100 (arom.), 2939, 2810 (aliph.), 1678 (CO), 1610 (CN). ^1^H NMR (500 MHz, DMSO-d_6_): *δ* 1.72 (d, *J* = 6.5 Hz, 3H, CH_3_ of L-norephedrine), 4.24 (t, *J* = 7.0 Hz, 1H, N–CH), 5.86 (d, *J* = 7.5 Hz, 1H, S–CH), 7.10–7.85 (9 aromatic), 8.12 (d, 1H, NH). ^13^C NMR (126 MHz, DMSO-d_6_): *δ* 14.75 (CH_3_), 45.98 (N–CH), 72.69 (S–CH), 119.58, 125.83, 126.16, 126.30 (2), 126.50, 127.54, 127.69 (2), 134.84, 141.95, 145.64, 152.85, 161.10. MS *m*/*z* (%): 309 (M^+^) (17), 79 (100). Anal. Calcd. For C_17_H_15_N_3_OS (309): C, 66.00; H, 4.89; N, 13.58. Found: C, 66.31; H, 5.16; N, 13.89.

**11:** Yield, 23%; m.p. 79.5 °C. IR (KBr, cm^−1^): 3460 (OH), 3391, 3312, 3215 (NH_2_, NH), 3072 (arom.), 2946, 2815 (aliph.), 1678 (CO), 1608 (CN). ^1^H NMR (500 MHz, DMSO-d_6_): *δ* 0.96 (bs, 3H, CH_3_ of L-norephedrine), 3.43 (s, 1H, NH), 4.29 (bs, 1H, N–CH), 4.91 (bs, 1H, O–CH), 5.57 (s, 2H, NH_2_), 5.73 (s, 1H, OH), 7.13–7.45 (8 aromatic), 7.93 (d, *J* = 7.2 Hz, 1H, aromatic). ^13^C NMR (126 MHz, DMSO-d_6_): *δ* 13.44 (CH_3_), 51.74 (N–CH), 73.43 (O–CH), 116.47, 121.40, 124.49, 125.97, 126.48 (2), 126.59, 127.29, 127.91 (2), 133.94, 143.01, 150.25, 161.10. MS *m*/*z* (%): 310 (M^+^) (21) 78 (100). Anal. Calcd. For C_17_H_18_N_4_O_2_ (310): C, 65.79; H, 5.85; N, 18.05. Found: C, 66.15; H, 6.11; N, 18.39.

#### 2.1.7. 3-Amino-2-hydroxyquinazolin-4(3H)-one (12), 3-amino-2-ethoxyquinazolin-4(3H)-one (13) and [1,2,4,5]tetrazino[3,2-b:6,5-b']diquinazoline-8,16(6H,14H)-dione (14)

A mixture of **7** (0.207 g, 0.001 mol) and **2** (0.151 g, 0.001 mol) was refluxed in ethanol 95% (30 mL) containing K_2_CO_3_ (0.138 g, 0.001 mol) for 12 h. The reaction was monitored by TLC and indicated the presence of two products **12** and **13**. The products were separated by silica gel column chromatography (45 × 2 i.d. cm, 30 gm) eluting with chloroform, followed by chloroform/methanol mixtures in a gradient system. Fractions 12–15 eluted with 2% methanol in chloroform afforded **13** (117 mg) after crystallization from methanol. Fractions 23–26 eluted with 5% methanol in chloroform afforded **12** (43 mg) after crystallization from methanol. When the reaction was repeated but in the presence of DMF instead of ethanol, dimer **14** was formed instead of **11**. The products obtained were crystallized from dioxane.

**12:** Yield, 24%; m.p. 294.1 °C. IR (KBr, cm^−1^): 3488 (OH), 3212, 3152 (NH_2_), 3055 (arom.), 1680 (CO), 1606 (CN). ^1^H NMR (500 MHz, DMSO-d_6_): *δ* 5.50 (s, 2H, NH_2_), 7.19–7.24 (m, 2H), 7.30–7.65 (m, 1H), 7.94 (dd, *J* = 1.2, 8.0 Hz, 1H), 11.62 (s, 1H, OH). ^13^C NMR (126 MHz, DMSO-d_6_): *δ* 113.87, 115.69, 123.02, 127.34, 134.93, 138.61, 148.93, 159.61. MS *m*/*z* (%): 177 (M^+^) (36), 118 (100). Anal. Calcd. For C_8_H_7_N_3_O_2_ (177): C, 54.24; H, 3.98; N, 23.72. Found: C, 54.52; H, 4.21; N, 24.01.

**13:** Yield, 57%; m.p. 102.8 °C. IR (KBr, cm^−1^): 3220, 3182 (NH_2_), 3099 (arom.), 2918, 2844 (aliph.), 1679 (CO), 1920 (CN). ^1^H NMR (500 MHz, DMSO-d_6_): *δ* 1.39 (t, *J* = 7.0, 3H, CH_3_), 4.49 (q, *J* = 7.0, 2H, CH_2_), 5.72 (s, 2H, NH_2_), 7.33 (t, *J* = 7.3, 1H), 7.44 (d, *J* = 8.0 Hz, 1H), 7.69 (m, 1H), 8.01 (dd, *J* = 1.0, 8.0 Hz, 1H). ^13^C NMR (126 MHz, DMSO-d_6_): *δ* 14.61 (CH_3_), 64.79 (CH_2_), 118.14, 124.56, 125.87, 126.59, 134.44, 146.12, 151.56, 160.30. MS *m*/*z* (%): 205 (M^+^) (32), 128 (100). Anal. Calcd. For C_10_H_11_N_3_O_2_ (205): C, 58.53; H, 5.40; N, 20.48. Found: C, 58.88; H, 5.76; N, 20.76.

**14:** Yield, 76%; m.p. 201.4 °C. IR (KBr, cm^−1^): 3217, 3176 (NH), 3075 (arom.), 1696 (CO), 1618 (CN). ^1^H NMR (500 MHz, DMSO-d_6_): *δ* 3.42 (bs, 2H, 2NH), 7.38 (t, *J* = 7.5 Hz, 2H), 7.50 (d, *J* = 7.5 Hz, 2H), 7.70 (t, *J* = 7.5 Hz, 2H), 8.02 (d, *J* = 7.5 Hz, 2H). ^13^C NMR (126 MHz, DMSO-d_6_): *δ* 120.34 (2), 125.93 (2), 126.40 (2), 128.73 (2), 134.94 (2), 148.79 (2), 156.84 (2), 161.67 (2). MS *m*/*z* (%): 318 (M^+^) (28), 158 (100). Anal. Calcd. For C_16_H_10_N_6_O_2_ (318): C, 60.38; H, 3.17; N, 26.40. Found: C, 60.07; H, 2.82; N, 26.07.

#### 2.1.8. 3-Methyl-2-phenyl-2H-oxazolo[2,3-b]quinazolin-5(3H)-one (15), 2,4-dimethyl-1,3-diphenyl-3,4-dihydro-2a,4a,9b-triazapentaleno[1,6-ab]naphthalen-5(2a^1^H)-one (16), 8,18-dimethyl-7,17-diphenyl-7,8,17,18-tetrahydro-[1,6,3,8]dioxadiazecino[2,3-b:7,8-b']diquinazoline-10,20-dione (17) and 3-(1-hydroxy-1-phenylpropan-2-yl)-2-(1-hydroxy-1-phenylpropan-2-ylamino)quinazolin-4(3H)-one (18)

To a solution of **5** (0.326 g, 0.001 mol) in DMF (20 mL) containing K_2_CO_3_ (0.138 g, 0.001 mol), L-norephedrine **2** (0.151 g, 0.001 mol) was added and refluxed for 10 h. The reaction mixture progress was monitored by TLC. It showed the presence of four products **15**, **16**, **17** and **18** that were separated by silica gel column chromatography (45 × 2 i.d. cm, 40 gm) eluting with chloroform, followed by chloroform/methanol mixtures in a gradient system. Fractions 3–8 eluted with chloroform afforded **15** (111 mg) after crystallization from methanol. Fractions 11–12 eluted with chloroform afforded **17** (56 mg) after crystallization from methanol. Fractions 16–18 eluted with 2% methanol in chloroform afforded **16** (47 mg) after crystallization from methanol. Fractions 27–29 eluted with 5% methanol in chloroform afforded **18** (34 mg) after crystallization from methanol.

**15:** Yield, 40%; m.p. 136.6 °C. IR (KBr, cm^−1^): 3048 (arom.), 2970, 2816 (aliph.), 1690 (CO), 1621 (CN). ^1^H NMR (500 MHz, DMSO-d_6_): *δ* 0.95 (d, *J* = 6.7 Hz, 3H, CH_3_ of L-norephedrine), 5.17 (p, *J* = 7.6 Hz, 1H, N–CH), 6.18 (d, *J* = 7.6 Hz, 1H, O–CH), 7.35–8.08 (m, 9H). ^13^C NMR (126 MHz, DMSO-d_6_): *δ* 14.45 (CH_3_), 54.49 (N–CH), 82.37 (O–CH), 119.33, 124.90, 126.31, 126.61, 126.80 (2), 128.90 (2), 129.15, 134.14, 135.12, 149.24, 155.30, 160.34. MS *m*/*z* (%): 278 (M^+^) (44), 77 (100). Anal. Calcd. For C_17_H_14_N_2_O_2_ (278): C, 73.37; H, 5.07; N, 10.07. Found: C, 73.65; H, 5.32; N, 10.32.

**16:** Yield, 12%; m.p. >350 °C. IR (KBr, cm^−1^): 3077 (arom.), 2927, 2846 (aliph.), 1693 (CO). ^1^H NMR (500 MHz, DMSO-d_6_): *δ* 1.23 (d, *J* = 11.5 Hz, 3H, CH_3_ of L-norephedrine), 1.24 (d, *J* = 12.0 Hz, 3H, CH_3_ of L-norephedrine), 4.73 (d, *J* = 11.0 Hz, 1H), 4.79 (d, *J* = 12.0 Hz, 1H), 5.33 (p, *J* = 7.0, 1H), 5.46 (p, *J* = 7.2, 1H), 7.21–8.00 (m, 14H, aromatic). ^13^C NMR (126 MHz, DMSO-d_6_): *δ* 16.49 (CH_3_), 16.74 (CH_3_), 51.18 (N–CH), 52.64 (N–CH), 52.99 (N–CH), 53.43 (N–CH), 113.76, 114.90, 115.49, 123.07, 127.69, 127.90, 128.41 (2), 128.74 (2), 129.17, 135.55, 135.63, 139.89, 140.12, 140.84, 150.24, 151.33, 162.77, 163.12. MS *m*/*z* (%): 394 (M^+^) (14), 235 (100). Anal. Calcd. For C_26_H_24_N_3_O (394): C, 79.16; H, 6.13; N, 10.65. Found: C, 79.51; H, 6.35; N, 10.89.

**17:** Yield, 10%; semisolid. IR (KBr, cm^−1^): 3101 (arom.), 2933, 2818 (aliph.), 1690 (2CO), 1622 (2CN). ^1^H NMR (500 MHz, DMSO-d_6_): *δ* 0.60 (d, *J* = 6.2 Hz, 6H, 2CH_3_ of L-norephedrine), 4.16 (P, *J* = 1.0 Hz, 2H, 2 (N–CH)), 5.68 (d, *J* = 8.5 Hz, 2H, 2 (O–CH)), 7.12–7.87 (m, 18H). ^13^C NMR (126 MHz, DMSO-d_6_): *δ* 13.74 (2), 53.02 (2) (N*–*CH), 79.94 (2) (O–CH), 113.57 (2), 123.47 (2), 126.53 (2), 127.99 (2), 128.58 (4),128.81 (4), 132.72 (2), 136.34 (2), 139.62 (2), 149.71 (2), 158.88 (2), 161.80 (2). MS *m*/*z* (%): 556 (M^+^) (13), 278 (100). Anal. Calcd. For C_34_H_28_N_4_O_4_ (556): C, 73.37; H, 5.07; N, 10.07. Found: C, 73.08; H, 4.89; N, 9.11.

**18:** Yield, 8%; m.p. 88.8 °C. IR (KBr, cm^−1^): 3450 (2OH), 3321(NH), 3079 (arom.), 2976, 2823 (aliph.), 1680 (CO), 1618 (CN). ^1^H NMR (500 MHz, DMSO-d_6_): *δ* 1.58 (d, *J* = 6.6 Hz, 6H, 2 (CH_3_) of L-norephedrine), 4.15, 4.40 (bs, 1NH), 5.10 (bs, *J* = 11.5 Hz, 2H, 2 (N–CH)), 5.24 (m, 2H, 2 (O–CH)), 5.62 (d, *J* = 5.3 Hz, 2H, 2 (OH)), 7.01–7.55 (m, 14H, aromatic). ^13^C NMR (126 MHz, DMSO-d_6_): *δ* 15.31 (CH_3_) (2), 56.53 (N–CH) (2), 74.05 (2), 115.14, 122.80 (2), 126.96, 127.63, 128.03, 128.30 (4), 128.65 (4), 135.28, 139.58 (2), 143.50, 150.47, 162.45. MS *m*/*z*: 430 (M^+^ + 1) (100). Anal. Calcd. For C_26_H_27_N_3_O_3_ (429): C, 72.71; H, 6.34; N, 9.78. Found: C, 72.46; H, 6.08; N, 9.40.

### 2.2. Biological evaluation

#### 2.2.1. MTT assay

MDA-MB-231, MCF-7, HepG-2, HCT-116 cancer cell lines and MCF-10A normal cells were obtained from American Type Culture Collection. The 96-well plate was incubated for 24 h before the MTT assay. The cell layer was washed with 0.25% (w/v) Trypsin, 0.53 mM EDTA solution. Cells were cultured using DMEM supplemented with 10% foetal bovine serum, 10 µg/mL insulin and 1% penicillin–streptomycin. Reconstituted MTT (10%) was added and incubated for 2 h. Formazan crystals were dissolved by the MTT solubilizing solution after incubation. Absorbance was measured at a wavelength of 570 nm^28^. IC_50_ was estimated according to the equation of Boltzmann sigmoidal concentration–response curve and compared to erlotinib and staurosporine.

#### 2.2.2. EGFR assay

EGFR kinase kit (0.192 mg/mL) was obtained from Invitrogen. An ATP solution and a kinase/peptide mixture were developed just before use. The solution on the plate was mixed carefully and incubated for 1 h at 25 °C. Then, 5 mL of the prepared solution was added to each well. The plate was incubated for 1 h and determined by an ELISA Reader (PerkinElmer, USA). Curve fitting using Graph Pad Prism 5 was constructed. Each experiment was repeated three times. IC_50_ was represented as means ± SE.

#### 2.2.3. Radiosensitizing evaluation

Irradiation was performed at the National Centre for Radiation Research and Technology (NCRRT), Egyptian Atomic Energy Authority, using Gamma cell-40 (^137^Cs) source. The promising compounds **5** and **6** were selected to be re-evaluated for *in vitro* cytotoxic activity after the cells containing the compounds were gamma-irradiated at a dose level of 8 Gy with a dose rate of 0.758 rad/s for 17.59 min. Cytotoxicity was measured two days after irradiation. The IC_50_ of the tested compounds is calculated using GraphPad Prism 5.

#### 2.2.4. Cell cycle analysis

The MDA-MB-231 cells (10^5^/well) were incubated with compound **5** at its IC_50_. After 24 h, the cells were washed twice with PBS, then collected and fixed with ice-cold ethanol 70% (v/v). The cells were re-suspended with 0.1 mg/mL RNase, stained with 40 mg/mL PI and examined using flow cytometry (FACScalibur-Becton Dickinson).

#### 2.2.5. Apoptotic assay

Cells were prepared as previously mentioned. Treatment of cells (10^5^) with Annexin V-FITC and propidium iodide (PI) was by apoptosis detection kit [BD Biosciences, San Jose, CA]. The binding of Annexin V-FITC and PI was examined using flow cytometry FACScalibur (BD Biosciences, San Jose, CA). CellQuest software was used for performing quadrant analysis of co-ordinate dot plots.

### 2.3. Molecular docking

Docking studies were performed using Molecular Operating Environment software (MOE, 2015.10) provided by chemical computing group, Canada. The software was used to carry out the docking of the promising compounds in the receptor's active site. The protein crystal structure was obtained from the Protein Databank, PDB: 1M17 containing the EGFR enzyme co-crystallized with erlotinib. All the water molecules were removed, 3D protonation was performed. The pocket was determined by the alpha triangle matcher technique. The Energy Minimization was performed using MMFF94X force field with RMSD gradient of 0.001 kcal mol^−1 ^Å^−1^ and the partial charges were calculated. The co-crystallized ligand was self-docked inside the active site. The compounds to be docked were drawn on ChemBioOffice 12 and copied as smiles to MOE followed by docking of **5** and **6**.

## 3. Results and discussion

### 3.1. Chemistry

**Scheme 1.**
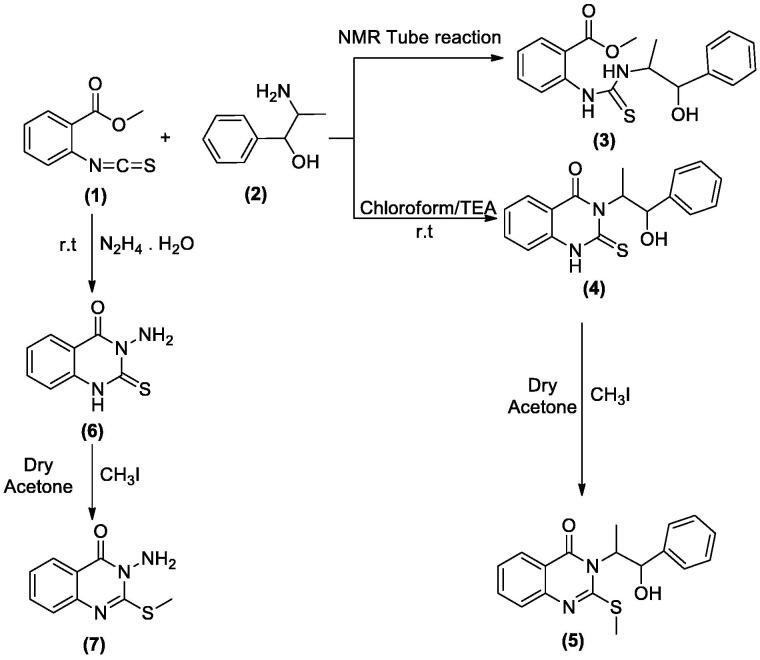
Synthesis of derivatives **3**–**7**.

The behaviour of the methyl 2-isothiocyanatobenzoate **1** towards the natural alkaloid L-norephedrine **2** was studied. When the isothiocyanate **1** reacted with **2** in chloroform containing a catalytic amount of triethylamine (TEA) at room temperature the unexpected quinazolinone **4** was formed despite the prospective thiourea derivative **3**. In a trial to obtain the open-chain thiourea derivative **3**, NMR tube reaction was carried out between **1** and **2**. The structure of **3** was confirmed by the presence of O–CH_3_ singlet signal at *δ*_H_ 3.83; *δ*_C_ 55.76 ppm in the ^1^H and ^13 ^C NMR, ester carbonyl at *δ*_C_ 167.96 ppm, C=S at *δ*_C_ 179.68 and the two NH singlets at *δ*_H_ 2.87 and 10.31 ppm. The M^+^ at 344 *m*/*z* confirmed the structure of **3**. While in NMR data of **4** the O–CH_3_, as well as one NH signal disappeared. The ester carbonyl signal in the ^13^C NMR of **3** was replaced by amide carbonyl at *δ*_C_ 159.57 ppm in **4** while C=S appeared at *δ*_C_ 176.01 ppm. Methylation of **4** by methyl iodide (MeI) in dry acetone in the presence of anhydrous K_2_CO_3_ gave the corresponding 2-(methyl thio)quinazolin-4(*3H*)-one derivative **5**. Both ^1^H and ^13^C NMR supported the structure of **5** by the appearance of S–CH_3_ signals at *δ*_H_ 2.47; *δ*_C_ 15.37 ppm. Moreover, the C=S signal at *δ*_C_ 176.01 ppm in **4** was replaced by C=N signal at *δ*_C_ 161.09 ppm in **5.** On the other hand, the reaction of **1** with hydrazine hydrate afforded the reported quinazolinone derivative **6**^26^, which was further reacted with MeI to give **7**. The structure of **6** showed NH proton singlet at *δ*_H_ 7.99 and carbon C=S signal at *δ*_C_ 169.27 ppm. However, in **7** the C=S signal was replaced by C=N signal at *δ*_C_ 160.94 ppm. The NMR data of **7** also showed the S–CH_3_ signals at *δ*_H_ 2.44; *δ*_C_ 14.09 ppm. Both ^1^H NMR of **6** and **7** showed signals for NH_2_ at *δ*_H_ 6.39 and 5.77 ppm, respectively. The MS spectra of **6** and **7** at 193 and 207 *m*/*z*, respectively were in full support of the proposed structures (Scheme 1).

**Scheme 2.**
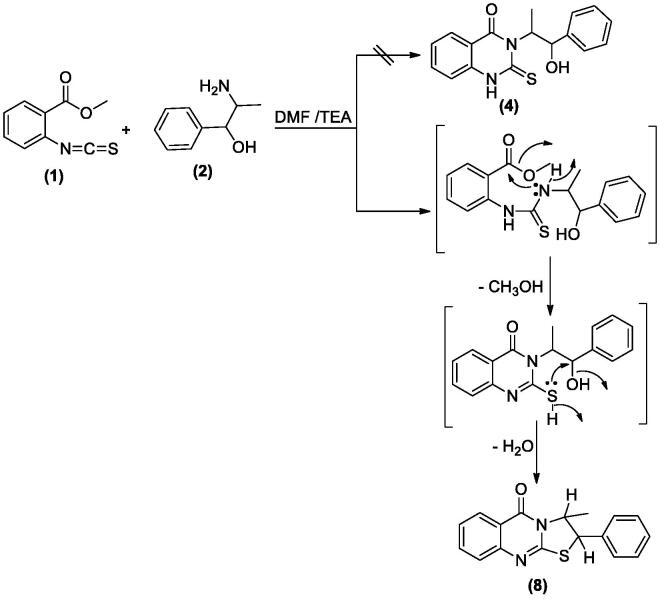
Synthesis of compound **8**.

**Figure 2.**
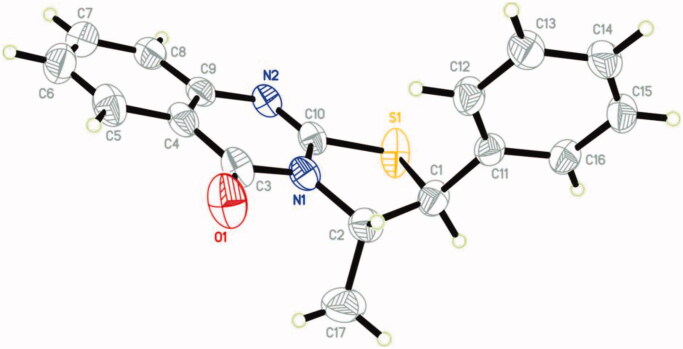
X-ray crystallographic structure of compound **8**.

When **1** reacted with **2** in DMF in the presence of a few drops of TEA yielded the thiazoloquinazolinone **8** rather than the expected **4** (Scheme 2, see Supplementary data 1). The structure of **8** was confirmed by different spectroscopic data and X-ray crystallographic analysis^29^ is displayed in Figure 2.

**Scheme 3.**
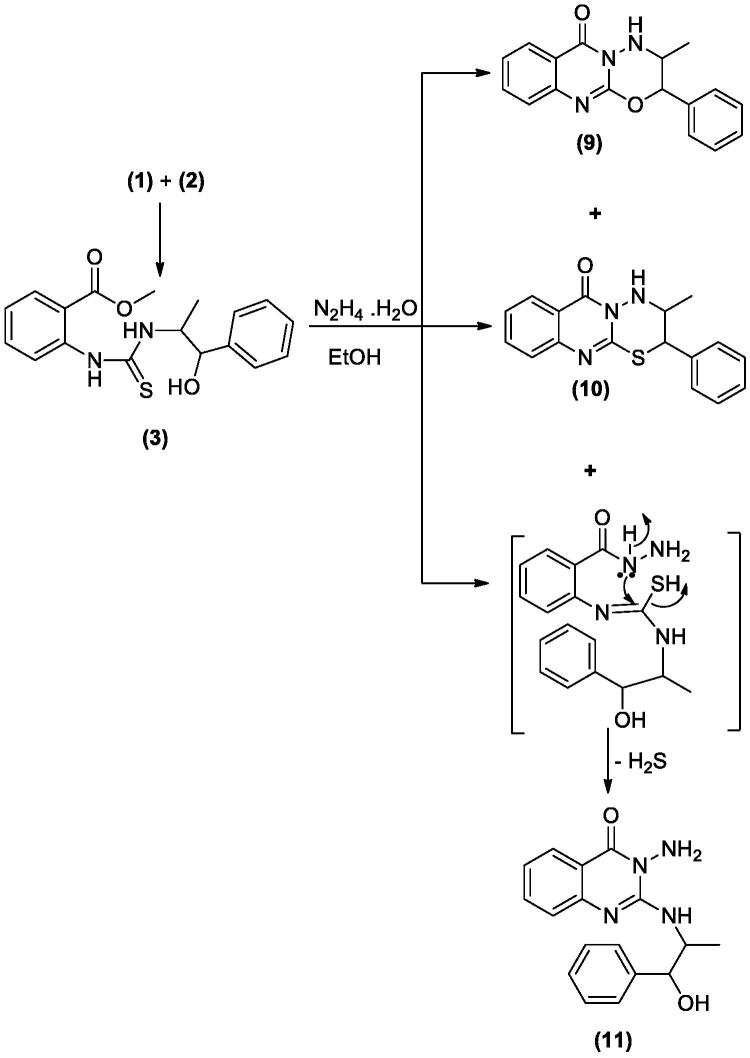
Synthesis of compounds **9**–**11**.

**Figure 3.**
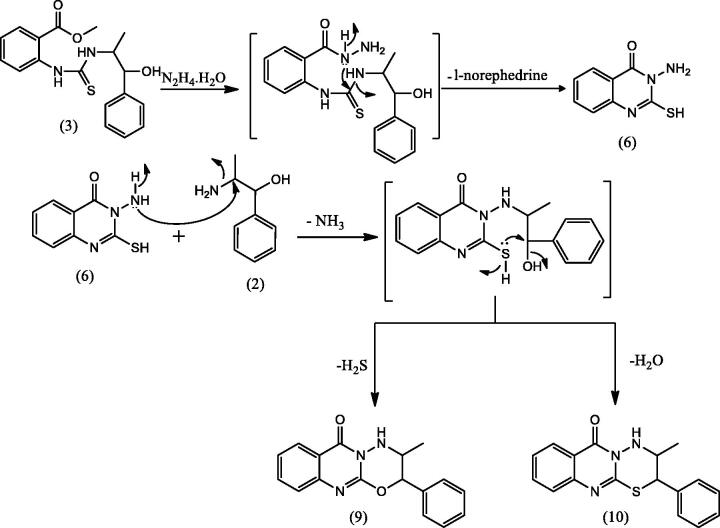
Formation of compounds **9** and **10**.

In Scheme 3, the interaction of **3** with hydrazine hydrate in absolute EtOH gives the expected product **11** in addition to two other derivatives **9** and **10** that were apparent in TLC. The mechanism of formation of **9** and **10** is explained in Figure 3. The ^1^H and ^13^C NMR data of **9** and **10** have common features like NH doublet at *δ*_H_ 8.01 and 8.12, O=C–N signal at *δ*_C_ 159.86 and 161.10 ppm, respectively. Both **9** and **10** showed signal at *δ*_C_ 154.84 and 152.85 ppm assigned for C=N carbons attached to another hetero atom, respectively. Compound **9** keeps the CH–O signals at *δ*_H_ 6.18 (d, *J* = 8.0); *δ*_C_ 81.86 ppm. These signals were replaced in **10** by *δ*_H_ 5.86 (d, *J* = 7.5); *δ*_C_ 72.69 ppm assigned for CH–S. The mass spectrum of **9** showed M^+^ at *m*/*z* 293 in complete agreement with the proposed structure, while the M^+^ at 309 *m*/*z* for **10** supported the replacement of one oxygen atom with a sulphur atom. The ^1^H NMR data of **11** showed signals for NH at *δ*_H_ 3.43 (s), NH_2_ at *δ*_H_ 5.57 (s) and OH at *δ*_H_ 5.73 (s) ppm. Moreover, the chemical shift of the CH–O signals at *δ*_H_ 4.91 (bs); *δ*_C_ 73.43 ppm indicated a non-substituted OH group. All these data proved that **11** lack the ring structure present in **9** and **10**. The M^+^ at 310 *m*/*z* further confirmed the formation of **11**.

**Scheme 4.**
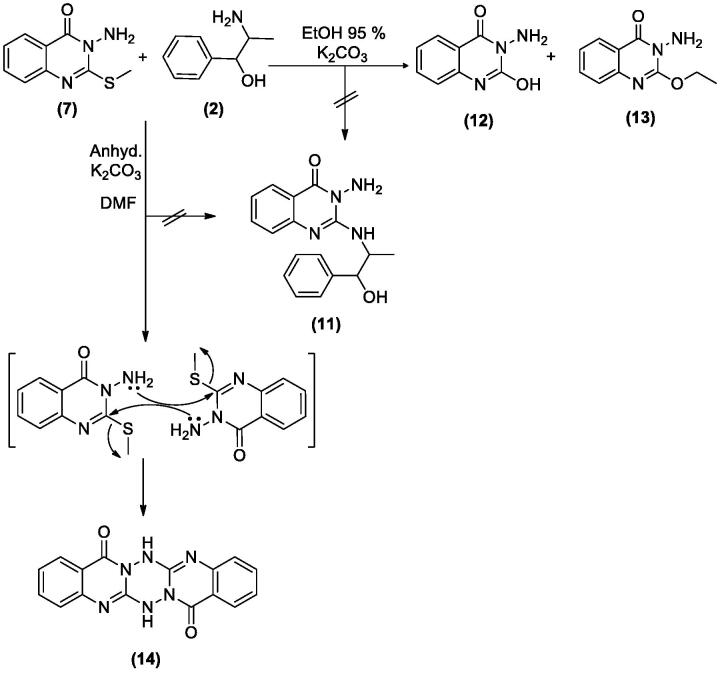
Synthesis of compounds **12**–**14**.

**Figure 4.**
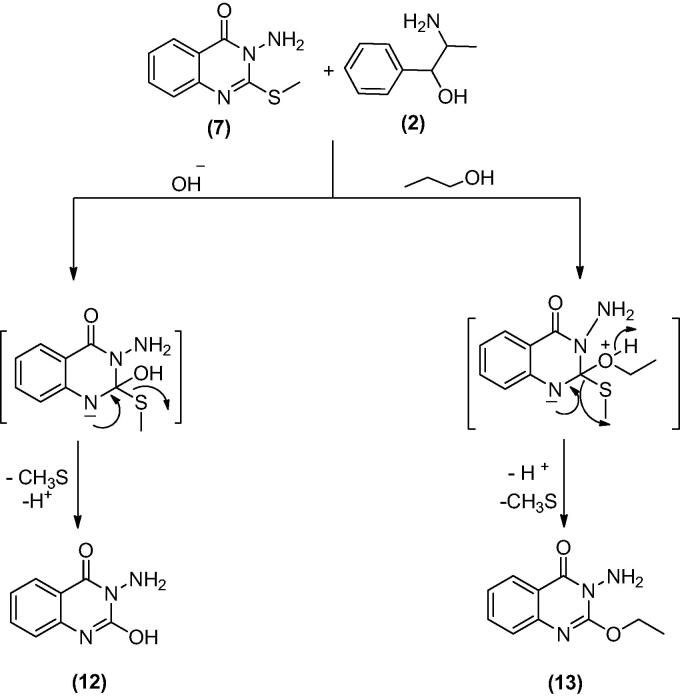
The mechanism of formation of compounds **12** and **13**.

Treatment of **7** with **2** in EtOH 95%, in the presence of K_2_CO_3_ afforded a mixture of **12** and **13**. While when the same reaction was repeated in DMF instead of EtOH the unexpected dimer **14** was formed. However, both reactions were expected to give **11** (Scheme 4). The formation of **12** and **13** was assumed to proceed *via* addition–elimination mechanisms, as depicted in Figure 4. The NMR data structure of **12** and **13** indicated the disappearance of the S–CH_3_ signals present in **7**. ^1^H NMR spectrum of **12** showed NH_2_ signal at *δ*_H_ 5.50 and OH signal at *δ*_H_ 11.62 ppm. The mass spectrum of **12** showed an M^+^ at 177 *m*/*z* provided further evidence for replacing the S–CH_3_ with OH group. In **13** signals for an ethoxy group at *δ*_H_ 1.39 (t, *J* = 7.0, CH_3_), *δ*_C_ 14.61 ppm and *δ*_H_ 4.49 (q, *J* = 7.0, CH_2_), *δ*_C_ 64.79 ppm along with M^+^ at 205 *m*/*z*, besides the disappearance of the S–CH_3_ signals present in **7**. The data of **14** indicated the replacement of NH_2_ signal by NH at *δ*_H_ 3.42 ppm. However, the MS data showed an M^+^ at 318 *m*/*z*, noting that **14** is formed *via* dimerization of **7**, as shown in Scheme 4.

**Scheme 5.**
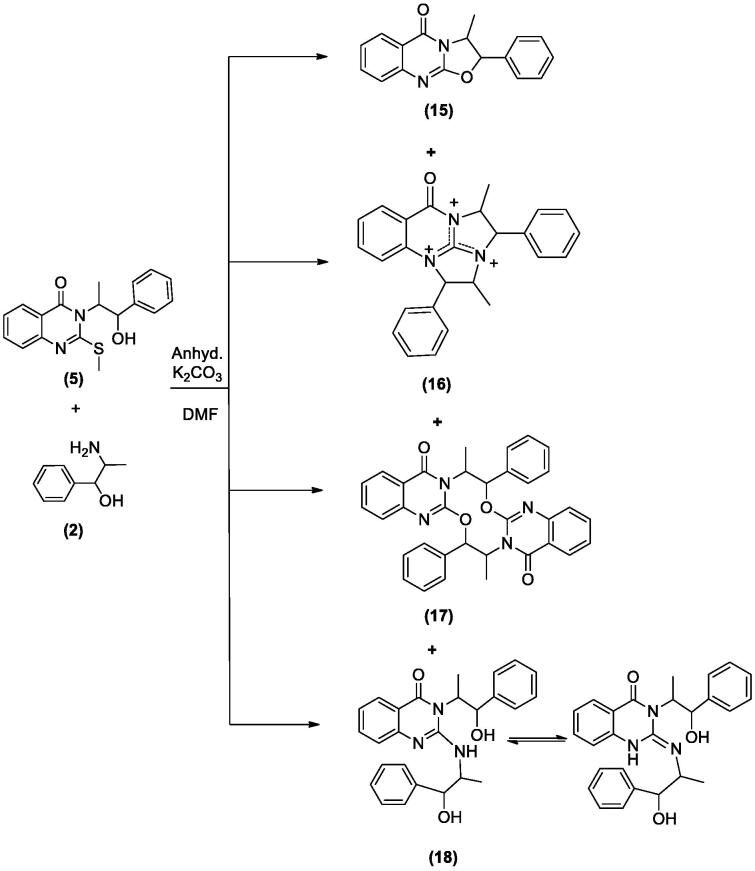
Synthesis of compounds **15**–**18**.

**Figure 5.**
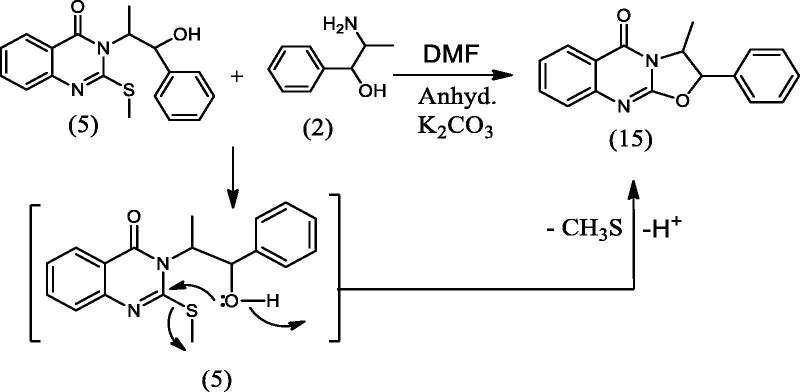
Formation of compound **15**.

**Figure 6.**
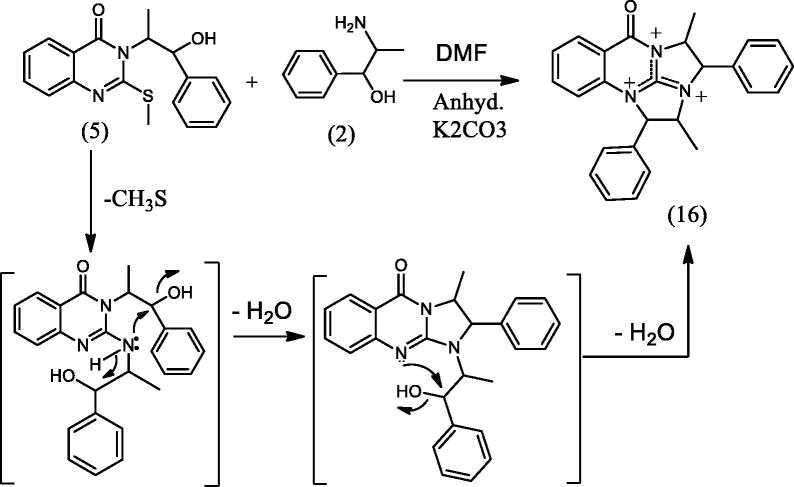
Formation of compound **16**.

**Figure 7.**
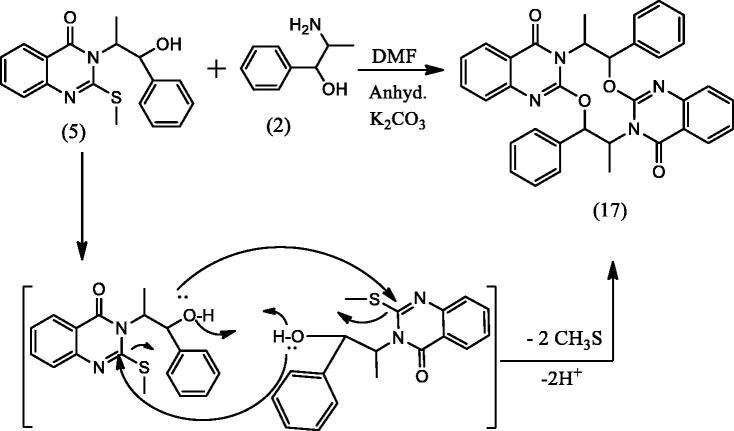
Formation of compound **17**.

In Scheme 5, the reaction of **5** with **2** in DMF containing K_2_CO_3_ afforded the expected product **18** in addition to three other products **15**, **16** and **17**. The mechanism of formation of **15**, **16** and **17** is present in Figures 5–7. The NMR data of **15** indicated the disappearance of the S–CH_3_ signals present in **5** and the downfield shift of the CH–O signals from *δ*_H_ 5.57, *δ*_C_ 72.80 ppm in **5** to *δ*_H_ 6.18, *δ*_C_ 82.37 ppm in **15**. These data were diagnostic for self-cyclization of **5**–**15** and were further supported by the mass data that showed M^+^ at 278 *m*/*z*. The reaction between one molecule of **5** and **2** according to Figure 6 resulted in the formation of the unique structure of **16**. The ^1^H and ^13^C NMR data of **16** indicated the presence of four CH–X groups at *δ*_H_ 4.73 (d, *J* = 11.0), *δ*_C_ 53.43; *δ*_H_ 4.79 (d, *J* = 12.0), *δ*_C_ 52.99, *δ*_H_ 5.33 (p, *J* = 7.0), *δ*_C_ 52.64 and *δ*_H_ 5.46 (p, *J* = 7.2), *δ*_C_ 51.18 ppm. The chemical shift indicated that none of the heteroatoms is oxygen, the formation of the complex ring structure involved water elimination and the presence of two methyl groups at *δ*_H_ 1.23 (d, *J* = 11.5), *δ*_C_ 16.49 and *δ*_H_ 1.24 (d, *J* = 12.0), *δ*_C_ 16.74 ppm. The M^+^ at *m*/*z* 394 was in complete agreement with the proposed structure of **16**. The NMR data of **17** indicated the disappearance of the S–CH_3_ signals and the OH signal at *δ*_H_ 5.87 ppm present in **5.** The CH–O signals in **17** showed a downfield shift to *δ*_H_ 5.68 (d, *J* = 8.5), *δ*_C_ 79.94 ppm diagnostic for derivatized oxygen atom. Mass spectrum showed M^+^ at 556 *m*/*z* consistent with the molecular formula C_34_H_28_N_4_O_4_ surely prove the dimeric nature of **17**. The addition of **2** *via* the elimination of S–CH_3_ resulted in **18**. The signals of two moieties of L-norephedrine were overlapped in both ^1^H and ^13^C NMR spectra. HR ESI showed a quasi-molecular ion at 430.2127 *m*/*z* (calc. 430.2131) for M^+^ + 1 ion certainly supporting the structure of **18**. The NH proton appeared as two broad singlets at 4.15, 4.40 each integrated for half proton diagnostic for the suggested tautomerisation in the structure. All the assignments of ^1^H and ^13^C NMR signals were performed based on DEPT 135 as well as 2D NMR experiments including COSY, HSQC and HMBC (see Supplementary data 2).

### 3.2. Biological evaluation

#### 3.2.1. *In vitro* cytotoxic activity evaluation

The *in vitro* cell viability activity of the targeted compounds **4**–**18** was measured through MTT assay against a panel of cell lines MDA-MB-231, MCF-7, HepG-2 and HCT-116 human cancer cell lines derived from breast, liver and colon tumors. A closer look at Table 1 indicates that compounds **4**–**18** showed variable IC_50_ values against the tested cell lines and was compared to erlotinib and staurosporine, as standards. Compound **5** was the most potent against all the cell lines with IC_50_ ranging from 1.53 to 5.76 µM. Compound **6** takes second place after **5** as a promising homologue. Compounds **5**, **6** and **15** displayed more potent activity against MDA-MB-231 cell line in comparison to erlotinib with IC_50_ values = 1.53, 1.60 and 2.41 versus 3.73 µM. While the remaining compounds showed good to moderate activities towards the tested cell lines. The most potent compounds **5** and **6** were screened against MCF-10A normal breast cell line to determine their selectivity and relative safety towards normal cells. The compounds showed low cytotoxic effect with IC_50_=61.85 and 49.21 µM against MCF-10A cell line. Measuring the selectivity index^30^ indicates that compounds **5** and **6** showed the highest selectivity towards MDA-MB-231 followed by HepG-2 cell lines (Table 2).

**Table 1.** Antiproliferative and EGFR inhibitory activity of the target compounds **4**–**18**.

Cpd no.	Structure	IC_50_ (µM)^a^				
		MDA-MB-231	MCF-7	HCT-116	HepG-2	EGFR
**4**	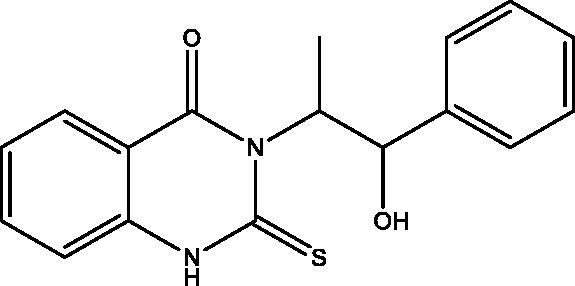	35.24 **±** 0.24	25.55 **±** 0.02	23.49 **±** 0.05	22.68 **±** 0.61	1.39 ± 0.14
**5**	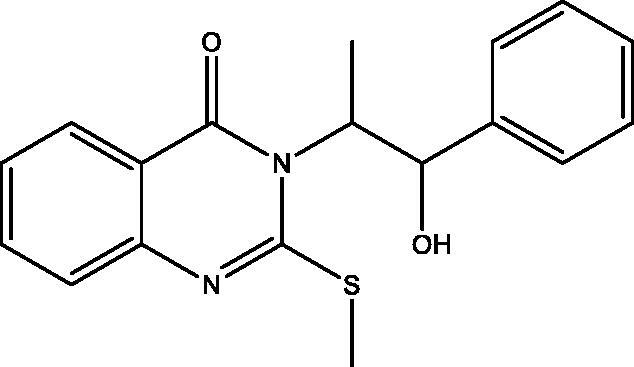	1.53 **±** 0.01	5.43 **±** 0.14	5.76 **±** 0.11	4.14 **±** 0.03	0.76 ± 0.10
**6**	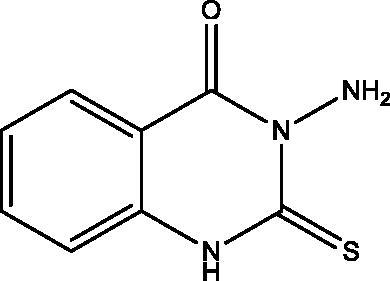	1.60 **±** 0.01	7.23 **±** 0.07	10.6 **±** 0.19	17.6 **±** 0.15	2.13 ± 0.21
**7**	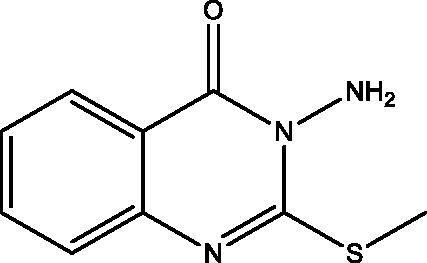	23.48 **±** 0.06	68.13 **±** 1.21	59.25 **±** 0.78	48.29 **±** 0.93	3.44 ± 0.15
**8**	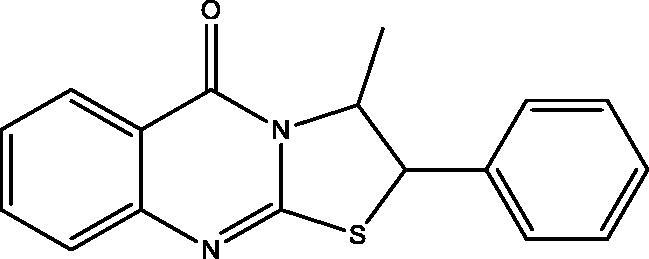	38.19 **±** 0.38	70.13 **±** 0.87	49.22 **±** 0.49	47.32 **±** 0.47	3.74 ± 0.17
**9**	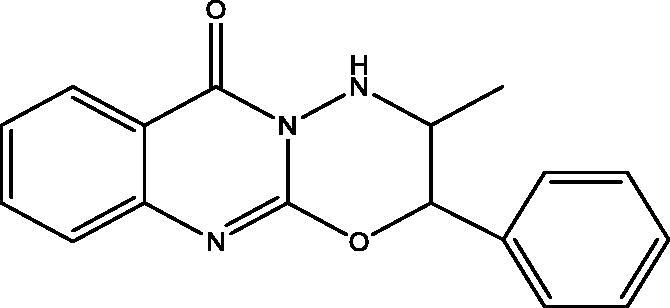	84.15 **±** 0.62	>100	90.54 ± 0.73	>100	9.78 ± 0.31
**10**	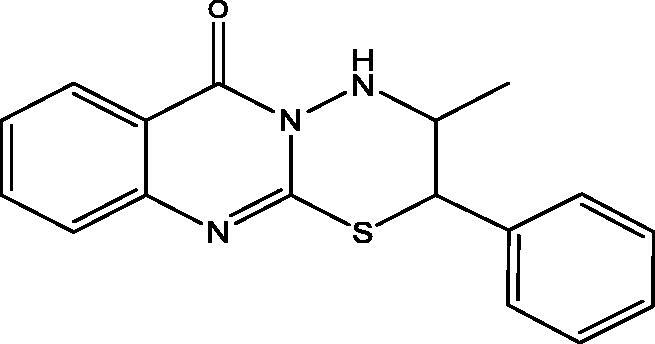	21.86 **±** 0.04	37.31 ± 0.29	46.27 **±** 0.63	35.58 **±** 0.36	3.09 ± 0.20
**11**	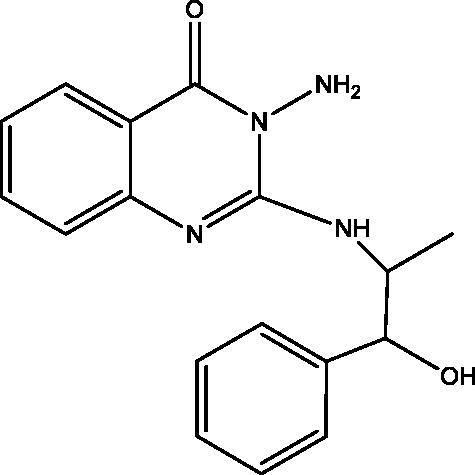	16.39 **±** 0.06	39.42 ± 0.30	44.15 **±** 0.22	33.65 **±** 0.26	3.87 ± 0.11
**12**	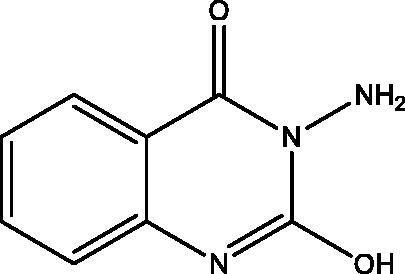	57.78 **±** 0.43	66.31 **±** 0.72	50.75 **±** 0.29	20.54 **±** 0.31	7.39 ± 0.28
**13**	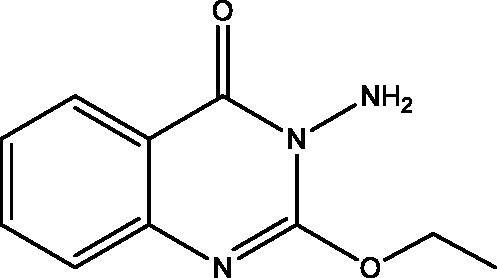	81.43 **±** 0.27	>100	>100	>100	8.35 ± 0.32
**14**	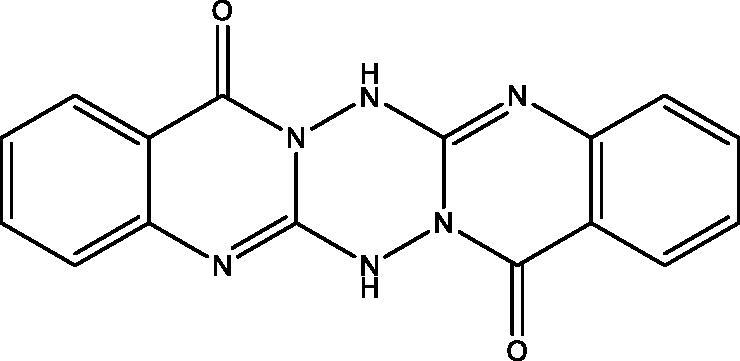	26.55 **±** 0.04	30.12 **±** 0.19	29.32 **±** 0.16	41.25 **±** 0.23	1.39 ± 0.27
**15**	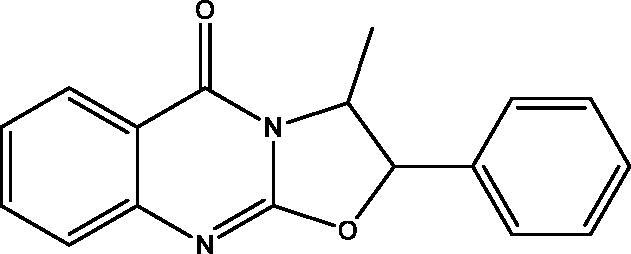	2.41 **±** 0.03	19.67 **±** 0.13	26.51 **±** 0.17	28.46 **±** 0.40	1.09 ± 0.12
**16**	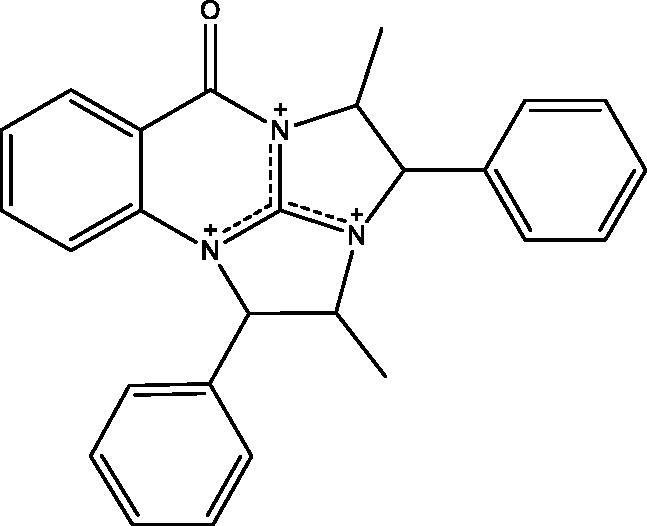	69.12 **±** 0.21	>100	>100	76.65 **±** 0.81	7.83 ± 0.19
**17**	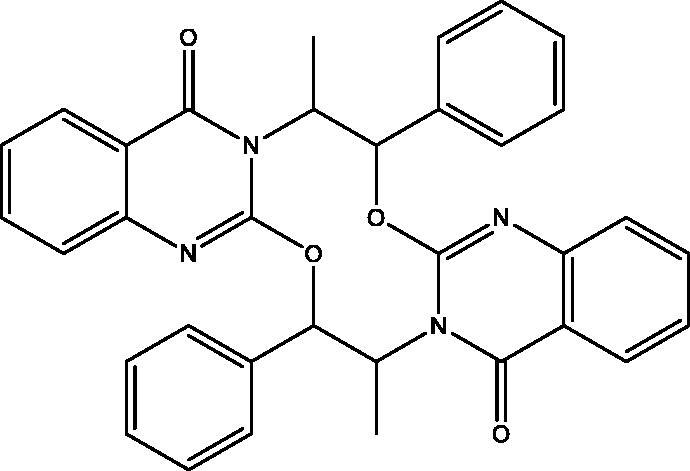	5.45 **±** 0.06	20.14 **±** 0.27	18.43 **±** 0.20	24.30 ± 0.18	1.02 ± 0.05
**18**	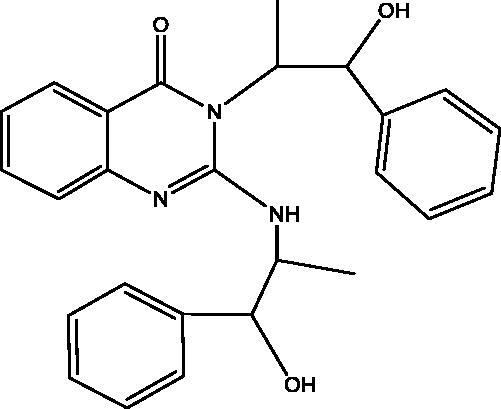	62.94 **±** 0.10	62.59 **±** 0.18	44.90 **±** 0.52	50.0 **±** 0.13	0.99 ± 0.04
**Erlotinib**	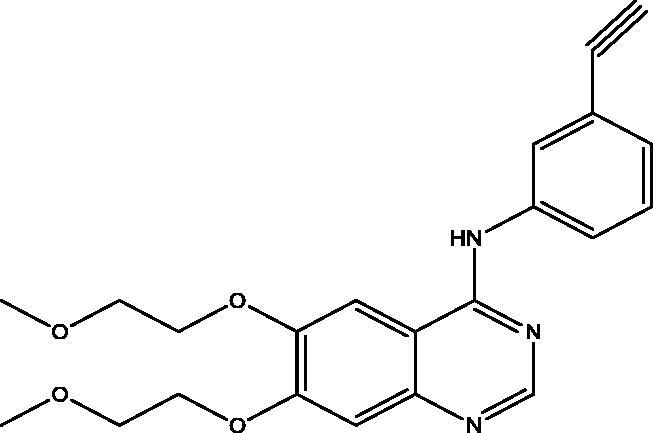	3.73 ± 0.01	4.48 **±** 0.02	2.78 **±** 0.04	3.04 **±** 0.20	0.31 ± 0.01
**Staurosporine**	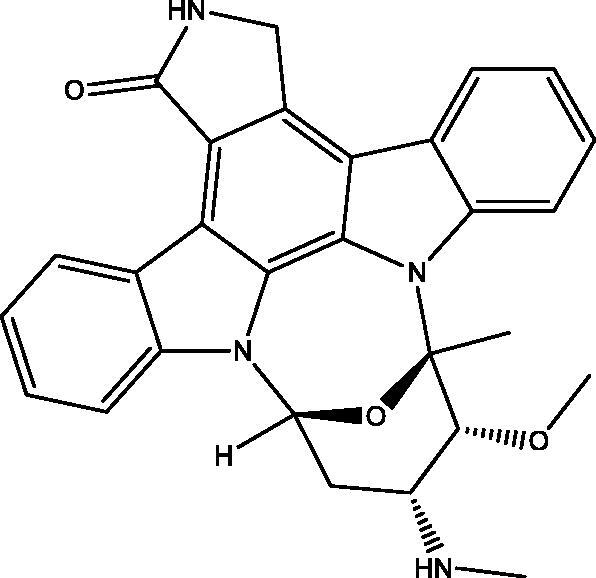	25.26 **±** 0.36	20.32 **±** 0.31	10.31 **±** 0.28	11.45 **±** 0.19	0.82 ± 0.08

^a^ The results represent the mean of three different experiments ± SE.

**Table 2.** The selectivity index of compounds **5** and **6** towards the tested cell lines.

Cpd no.	IC_50_ (µM)	Selectivity index (SI)			
	MCF-10A	MDA-MB-231	MCF-7	HCT-116	HepG-2
**5**	61.85 ± 2.14	40.42	11.39	10.74	14.93
**6**	49.21 ± 1.52	30.75	6.81	4.64	23.10

#### 3.2.2. EGFR kinase assay

All the newly synthesized compounds, **4–18**, were subjected to EGFR-TK inhibitory assay. Furthermore, a representative compound eliciting superior EGFR inhibition was subjected to cell cycle analysis and apoptotic assay to investigate its effect on cell cycle progression and apoptosis. Table 1 shows the inhibition data of EGFR (IC_50_ values) for the examined compounds, erlotinib and staurosporine, as reference standards. All analogues showed excellent EGFR inhibition potential ranging from 0.76 to 9.78 µM. The 3-(1-hydroxy-1-phenylpropan-2-yl)-2-(methylthio)quinazolin-4(*3H*)-one **5** demonstrated superior enzyme inhibition better than that expressed by erlotinib (IC_50_=0.76 versus 0.92 µM). Compound **5** is the most active compound towards all the tested cell lines and EGFR inhibitory activity with relative safety towards normal cells and high selectivity towards MDA-MB-231 cell line. The activity of **5** displayed a remarkable decrease by the replacement of the methyl mercaptan with the thione group as in **4**, while replacement with the 1-hydroxy-1-phenylpropan-2-ylamino group as in **18** demonstrates a narrow range change in activity from 0.76 to 0.99 µM. Furthermore, replacement of the 1-hydroxy-1-phenylpropan-2-yl in **4** with the amino group as in **6** leads to lowering the EGFR inhibitory activity (IC_50_ 1.39 versus 2.13 µM). Also, replacement of the thione group in **6** with methyl mercaptan **7**, 1-hydroxy-1-phenylpropan-2-ylamino **11**, hydroxy **12** or ethoxy group **13** reduces the activity. Regarding the two homologues **8** and **15**, the replacement of sulphur with oxygen greatly enhances the EGFR activity, while the opposite occurs in **9** and **10**. The two dimers **14** and **17** show very potent activity that demonstrates that a bulky rigid structure is favourable for binding with the receptor.

#### 3.2.3. Radiosensitizing activity

Radiotherapy is second to surgery in cancer treatment. The major drawback of radiotherapy is its inability to differentiate between cancerous and normal tissues. Radiation causes ionization and excitation of atoms that result in the generation of short-lived free radicals. These free radicals can damage proteins and membranes, leading to single or double DNA strand breaks^31^^,^^32^. A radiosensitizing agent can induce tumor sensitization to ionizing radiation, thus lowering the required dose for treatment. This enhancement of radiation effects not only control the local tumors but also limit the metastatic spread. EGFR inhibitors can adopt another mechanism of action by inhibiting accelerated repopulation of tumor cells during fractionated radiotherapy as they block the membrane receptors of growth factors or interfere with the signaling pathways involved in cell proliferation^33^^,^^34^.

The ability of the most active compounds **5** and **6** to enhance gamma radiation-induced tumor cell death was examined. The results proved the ability of the two compounds to sensitize the cancerous cells to the lethal effects of ionizing radiation (Table 3). Compounds **5** and **6** showed enhanced cytotoxicity on all cell lines after irradiation with a single dose of 8 Gy gamma radiation. Compound **5** was more potent on all the tested cell lines with IC_50_ < 5 µM.

**Table 3.** IC_50_ of compounds **5** and **6** on cancer cell lines after being subjected to irradiation.

Cpd no.	IC_50_ (µM)^a^ after irradiation			
	MDA-MB-231	MCF-7	HCT-116	HepG-2
**5**	0.78 **±** 0.15	2.35 **±** 0.18	2.28 **±** 0.41	2.34 **±** 0.23
**6**	1.04 **±** 0.04	4.26 **±** 0.01	6.98 **±** 0.25	14.26 **±** 0.11

^a^ The values represent the mean of three different experiments ± SE.

#### 3.2.4. Effect on cell cycle progression

**Figure 8.**
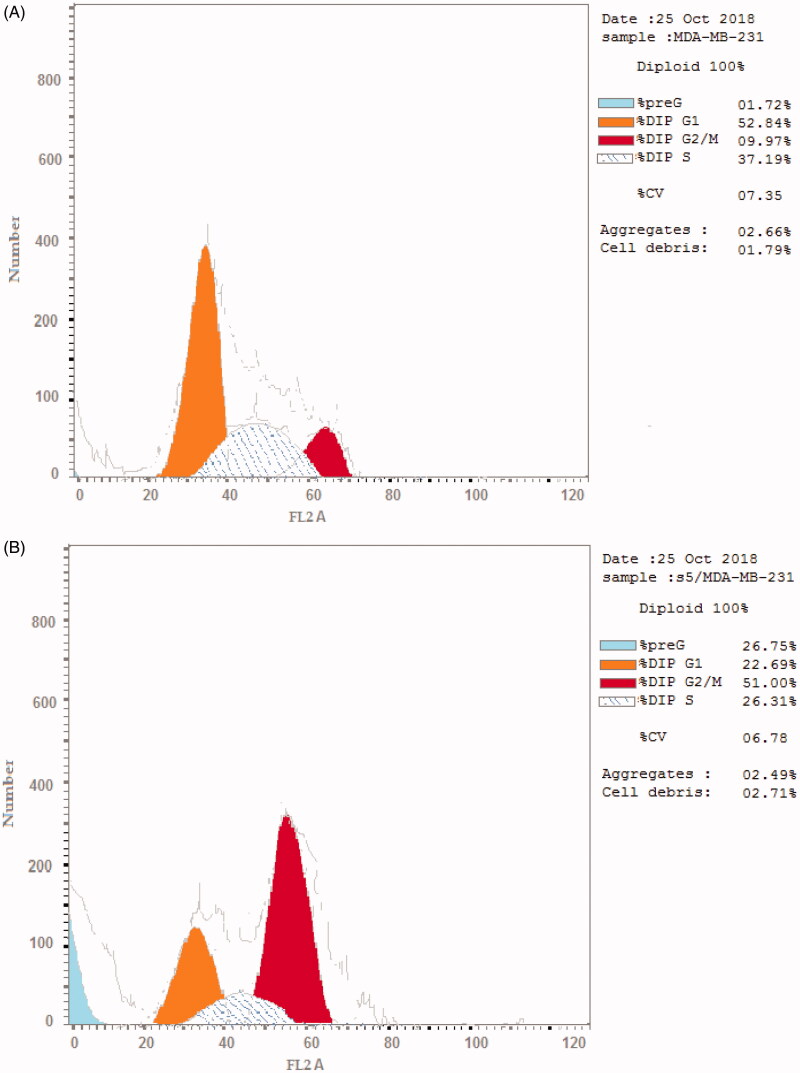
Flow cytometry analysis for MDA-MB-231 (A) control cells (B) compound **5**.

**Figure 9.**
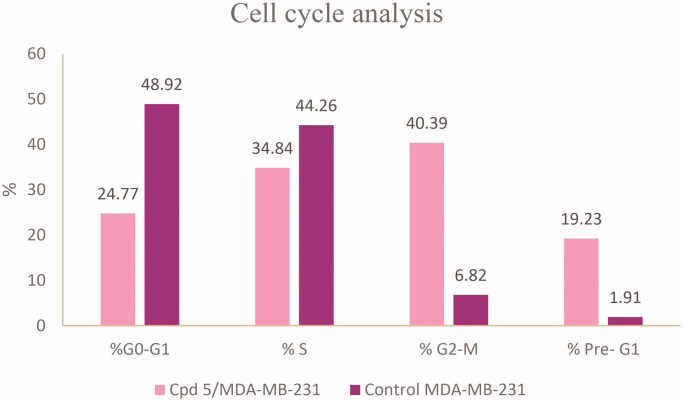
Histogram showing the effect of compound **5** on cell cycle analysis using MDA-MB-231 cell line.

The therapeutic effect of the anticancer agent depends upon its ability to stop cell cycle progression by arresting cell division at certain checkpoints promoting apoptosis. These checkpoints exist at G1-S, S and G2-M phases^35^^,^^36^. The most potent and selective compound **5** was chosen to determine its ability to induce apoptosis using MDA-MB-231 cells according to the reported method^37^. The cells were treated with compound **5** at a concentration equals to its IC_50_ value on EGFR (0.76 μM) for 24 h. It is clear from Figures 8 and 9 that compound **5** interfered with the cell cycle in the G2-M phase. At that phase, accumulating cells reached 40.39% after treatment of control MDA-MB-231 cells (6.82%) with compound **5**. Furthermore, compound **5** raised the percentage of cells at pri-G1 phase by 10 folds to reach 19.23% after being 1.91% in control cells. On the contrary, the cell population in G1 and S phases decrease after treatment with compound **5.** So, compound **5** induces apoptosis through cell cycle arrest in the G2-M phase.

#### 3.2.5. Apoptotic assay

**Figure 10.**
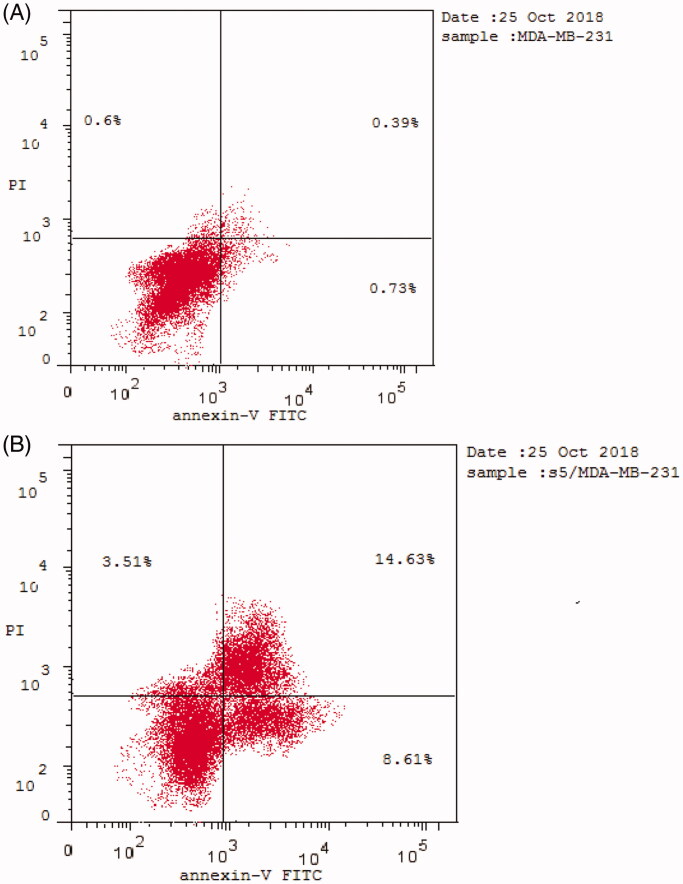
Effect of compound **5** on % of apoptotic cells using Annexin V/PI assay.

**Figure 11.**
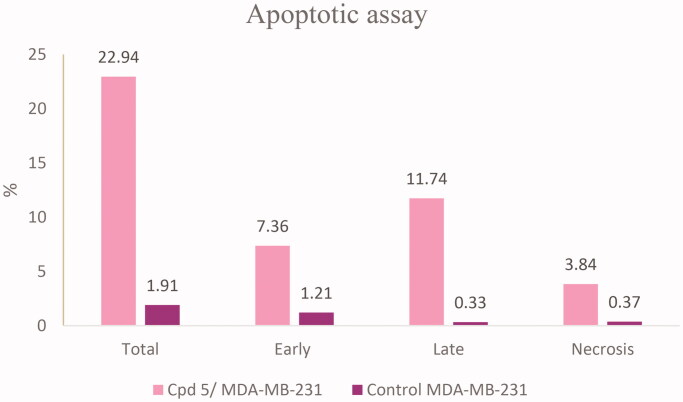
Induction of apoptosis by compound **5** in MDA-MB-231 cell line.

Phosphatidylserine (PS) exposure on the outer plasma membrane was detected during apoptosis and forms the basis for Annexin V/PI (propidium iodide) double staining assay to detect apoptotic cell death. At early apoptosis, the cell membrane excludes viability dyes such as PI and permits the determination of apoptotic cell kinetics according to the cell cycle^38^^,^^39^. To investigate the mode of induced cell death, MDA-MB-231 cells were incubated with compound **5** at 0.76 μM for 24 h. Compound **5** induced apoptosis (19.1%) by more than 12 folds over the control (1.54%). Compound **5** induced early apoptosis by 7.36% and enhanced late apoptosis by 11.74% compared with the untreated control cells (Figures 10 and 11).

### 3.3. Molecular docking

**Figure 12.**
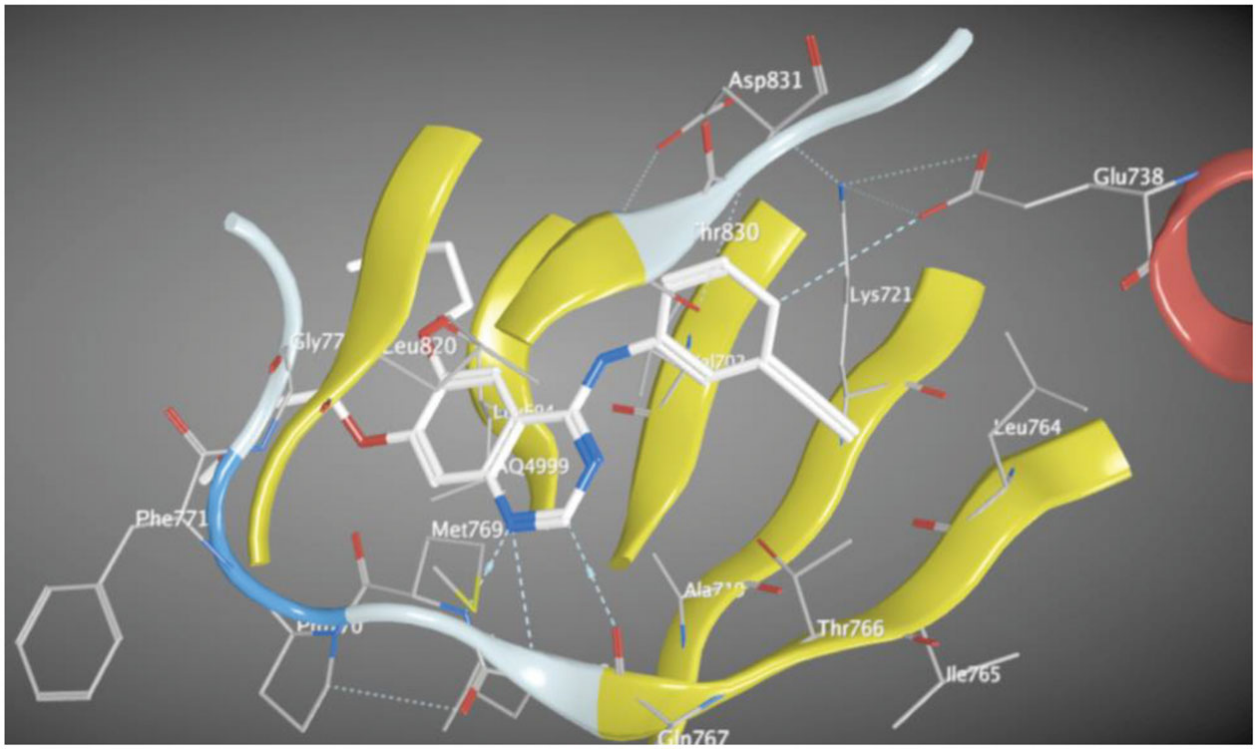
The docking pose of erlotinib inside the active site of **1M17**.

**Figure 13.**
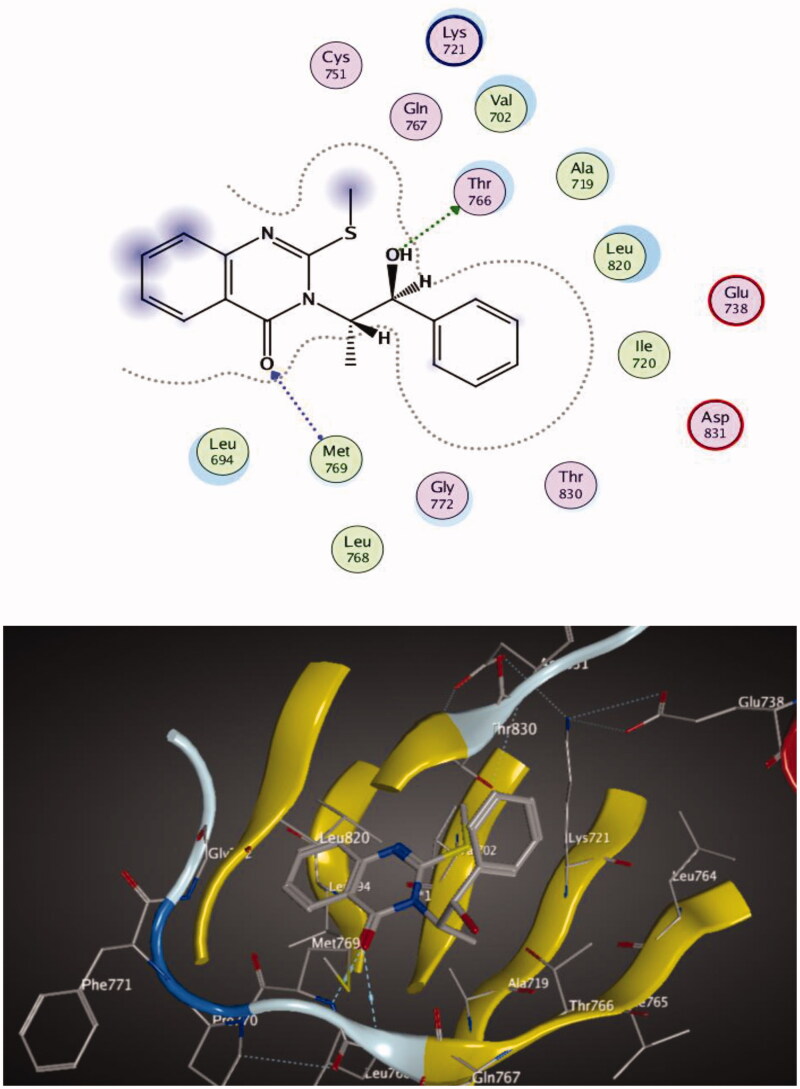
2D & 3D docking poses of compound **5** inside the active site of **1M17**.

**Figure 14.**
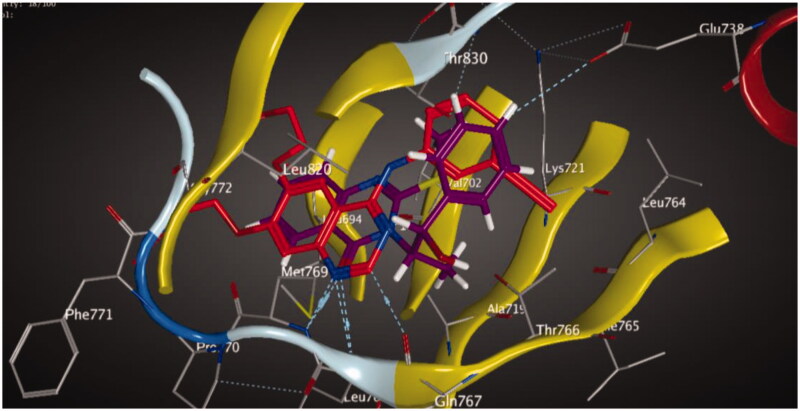
Superimposition of erlotinib (red) and compound **5** (magenta) showed that they adopt the same orientation inside the active site.

**Figure 15.**
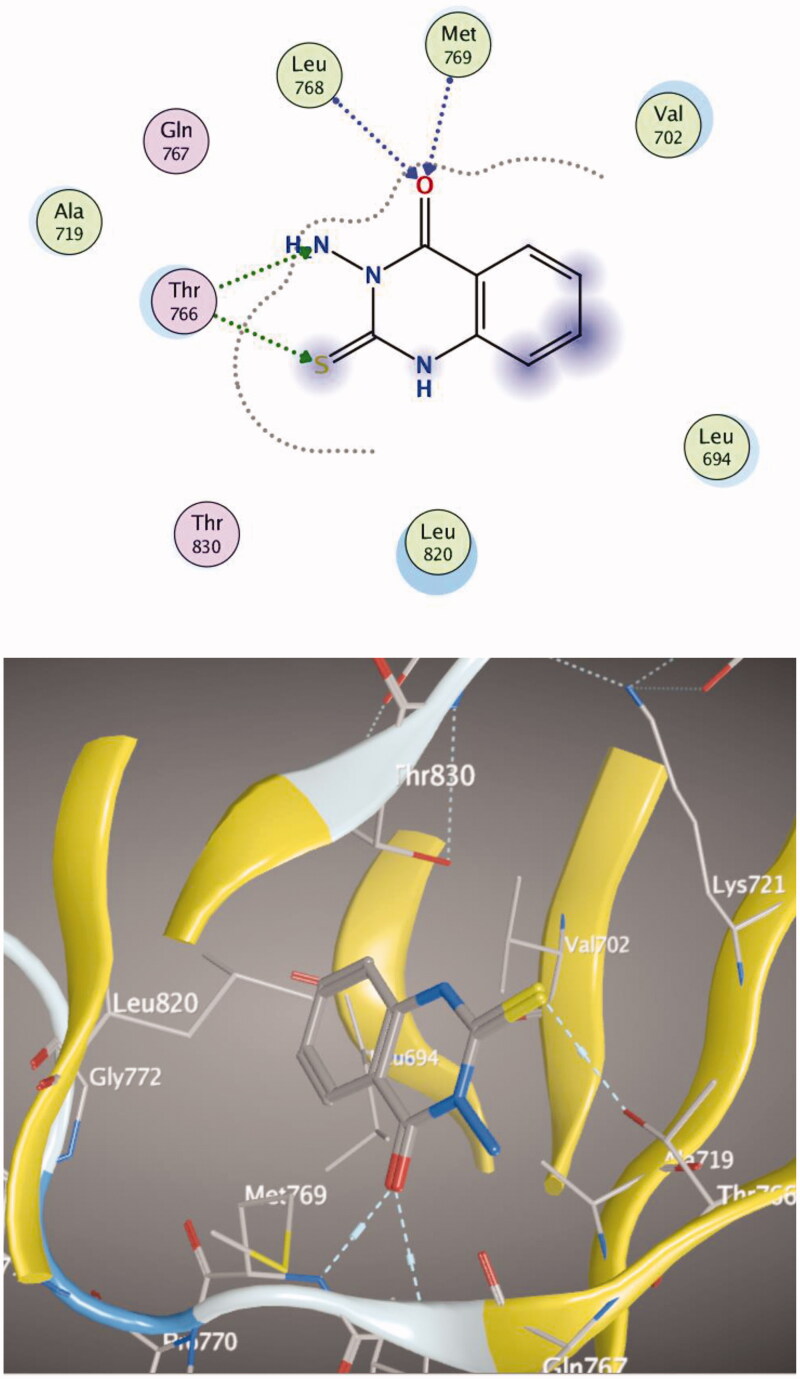
2D and 3D visuals of compound **6** inside 1M17 active site.

**Figure 16.**
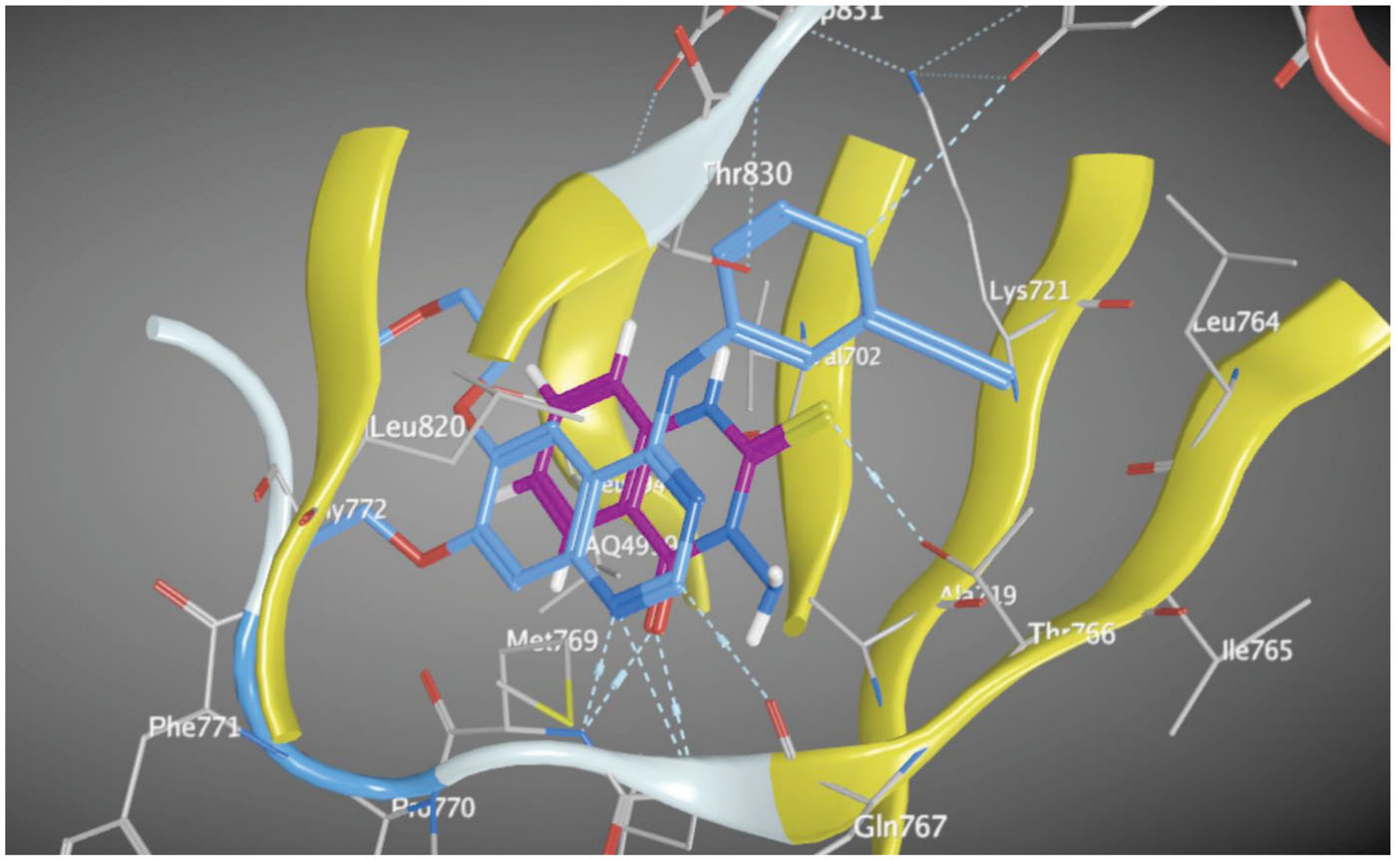
Overlaying of compound **6** (blue) and erlotinib (magenta) in the active site of EGFR.

Molecular docking of compounds **5** and **6** was performed on the active site of EGFR co-crystallized with erlotinib (PDB: 1M17) (Figure 12)^40^. The active site of 1M17 consists mainly of these key amino acids; Met 769, Leu 694, Thr 766, Ala 719, Leu 764, Gln 767, Leu 768, Pro 770, Phe 771, Gly 772, Leu 820, Thr 830 and Asp 831. The ligand compounds **5** and **6** were docked into the active site of the target protein 1M17 and the binding affinities, energy scores and RMSD values for compounds were recorded. Validation of molecular docking showed that the RMSD values are within acceptable limits (less than 2 Å)^41^. The best binding affinity with the lowest energy score for the compounds was computed as −10.14 kcal mol^−1^ (compound **5**) and −10.03 kcal mol^−1^ (compound **6**). According to these findings, together with the above-mentioned biological evaluation, compounds **5** and **6** may act as effective docking material for EGFR tyrosine kinase. The 2D and 3D visuals of the interaction map for compound **5** can be seen in Figure 13. The hydrogen bond formation connected the CO of quinazolinone with Met 769 of the target protein with a length of 2.39 Å and Thr 766 by OH with 2.63 Å. Superimposing compound **5** with erlotinib showed that they adopt the same orientation inside the active site with RMSD = 1.243 Å (Figure 14). On the other hand, four conventional hydrogen bond interactions were observed between compound **6** and the macromolecule 1M17 as follows; the CO of the quinazolinone with Met 769 and Leu 768 with a recorded distance of 2.54 and 2.79 Å, respectively. In addition to Thr 766 that forms two hydrogen bonds with NH and CS at a distance of 2.88 and 3.01 Å (Figure 15). Overlaying of erlotinib and compound **6** can be observed in Figure 16 with RMSD = 1.236 Å.

## 4. Conclusion

In this study, a novel series of quinazolinone and fused quinazolinone derivatives synthesized by the aid of L-norephedrine were obtained. All these compounds showed variable anticancer activity against MDA-MB-231, MCF-7, HepG-2 and HCT-116 cancer cell lines and EGFR inhibitory activity comparable to erlotinib. The 3-(1-hydroxy-1-phenylpropan-2-yl)-2-(methylthio)quinazolin-4(*3H*)-one **5** and 3-amino-2-thioxo-2,3-dihydroquinazolin-4(*1H*)-one **6** were the most promising in this series towards the cancer cell lines and EGFR. Compounds **5** and **6** were further selected to measure their relative safety and selectivity towards normal cells. They showed mild cytotoxic activity towards MCF-10A normal cell line and high selectivity towards MDA-MB-231 cell line. Besides, they displayed radiosensitizing activity through their ability to sensitize the cancer cells to the lethal effect of gamma irradiation. The most potent compound in this series, **5**, undergoes cell cycle analysis and annexin V/PI assay to detect apoptotic cell death. Compound **5** proved to arrest the cell cycle progression at the G2-M phase, induce early apoptosis and enhance late apoptosis. Moreover, molecular docking of compounds **5** and **6** showed the key interactions required for EGFR inhibition. Finally, compounds **5** and **6** could be considered as promising leads for the development of new anticancer and radiosensitizing agents.

## Supplementary Material

Supplemental Material
