# Supplementary material for: Synthesis of some quinazolinones inspired from the natural alkaloid L-norephedrine as EGFR inhibitors and radiosensitizers
Source: J Enzyme Inhib Med Chem. 2020 Dec 28;36(1):218–38. doi: 10.1080/14756366.2020.1854243 (PMC7781899; doi:10.1080/14756366.2020.1854243)

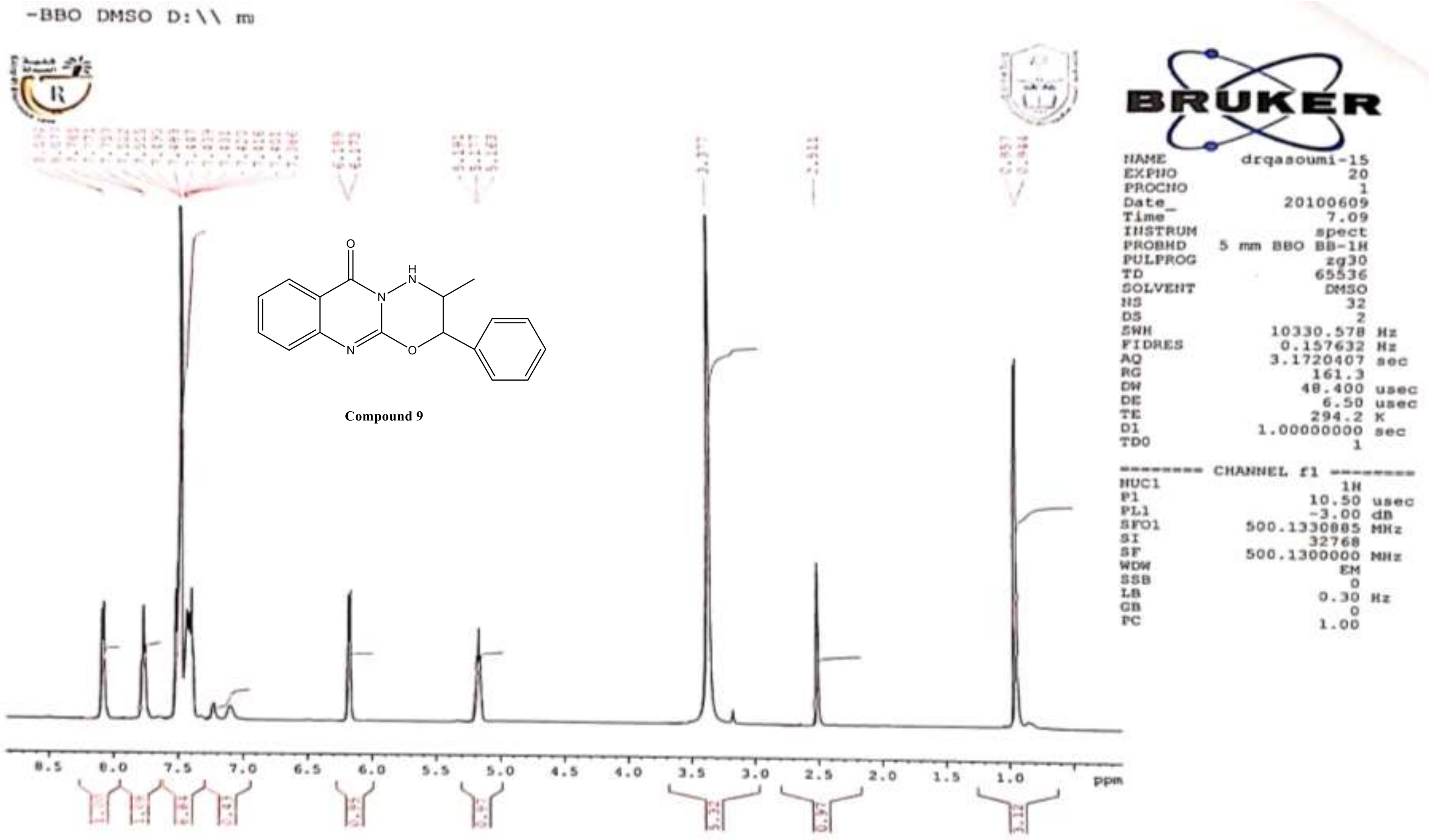
-BBO DMSO D:\\ m
BRUKER
NAME drqasoumi-15
EXPNO 20
PROCNO 1
Date_ 20100609
Time 7.09
INSTRUM spect
PROBHD 5 mm BBO BB-1H
PULPROG zg30
TD 65536
SOLVENT DMSO
NS 32
DS 2
SWH 10330.578 Hz
FIDRES 0.157632 Hz
AQ 3.1720407 sec
RG 161.3
DW 48.400 usec
DE 6.50 usec
TE 294.2 K
D1 1.00000000 sec
TD0 1
======== CHANNEL f1 ========
NUC1 1H
P1 10.50 usec
PL1 -3.00 dB
SFO1 500.1330885 MHz
SI 32768
SF 500.1300000 MHz
WDW EM
SSB 0
LB 0.30 Hz
GB 0
PC 1.00
8.5 8.0 7.5 7.0 6.5 6.0 5.5 5.0 4.5 4.0 3.5 3.0 2.5 2.0 1.5 1.0 ppm
O
H
N
N
O

Compound 9

3BO DMSO D:\\ mmj

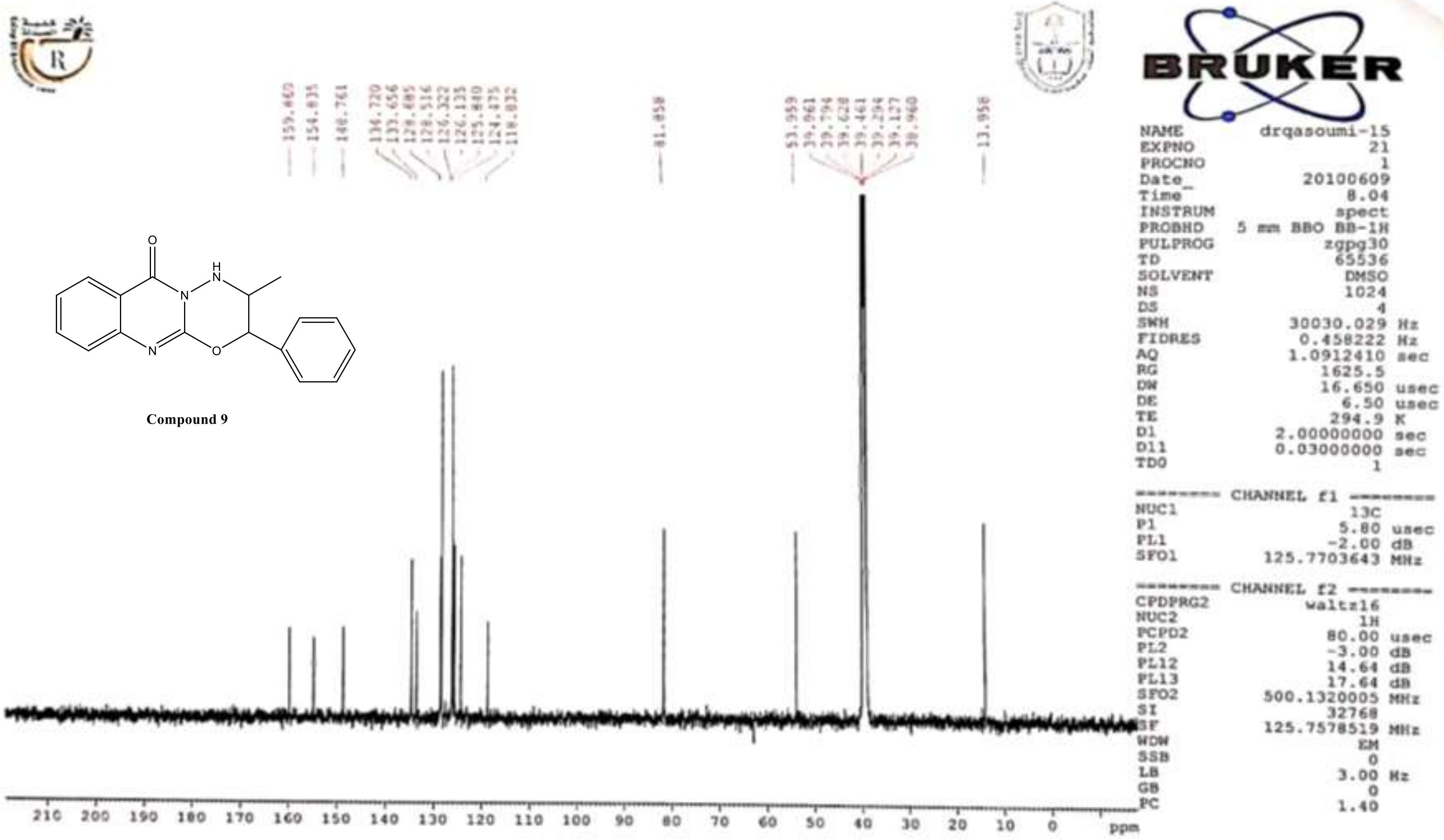

Compound 9

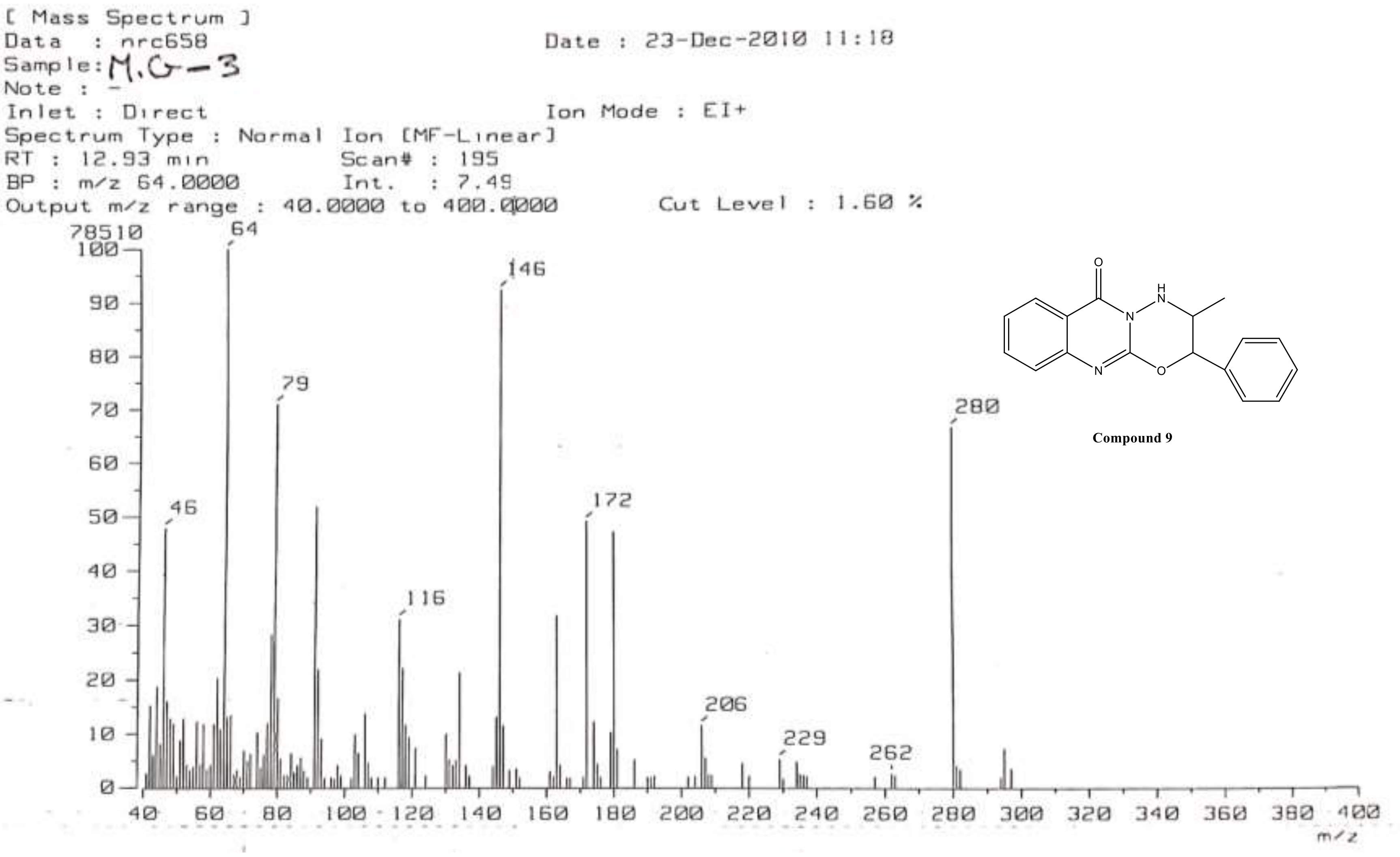
[Mass Spectrum]
Data : nrc658
Date : 23-Dec-2010 11:18
Sample: M.G—3
Note : -
Inlet : Direct
Ion Mode : EI+
Spectrum Type : Normal Ion [MF-Linear]
RT : 12.93 min
Scan# : 195
BP : m/z 64.0000
Int. : 7.49
Output m/z range : 40.0000 to 400.0000
Cut Level : 1.60 %
78510
64
146
79
280
46
172
116
206
229
262
m/z
O
H
N
N
N
O

Compound 9

-BBO DMSO D:\\ m

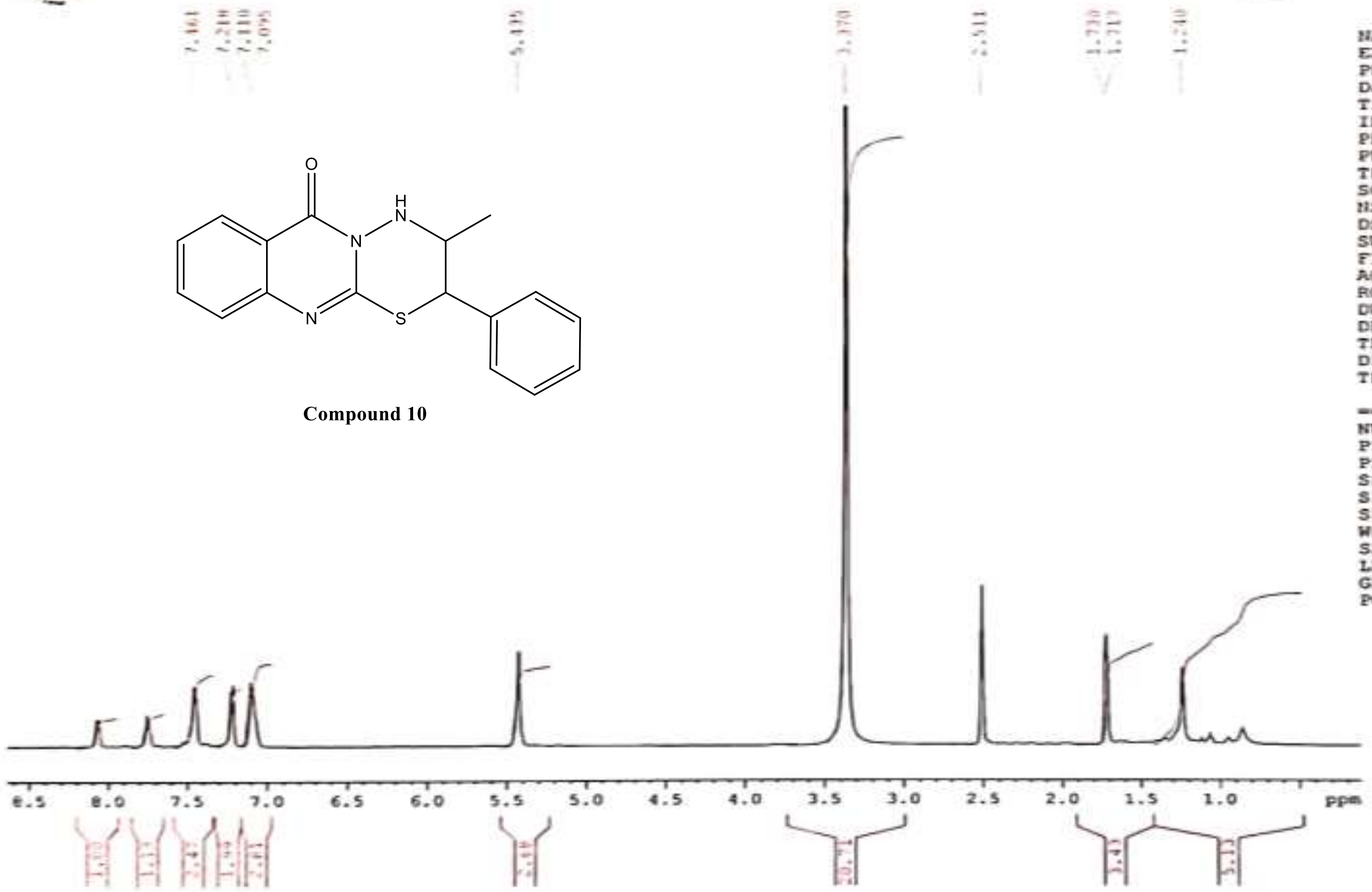

Compound 10

3BO DMSO D:\\ mmj

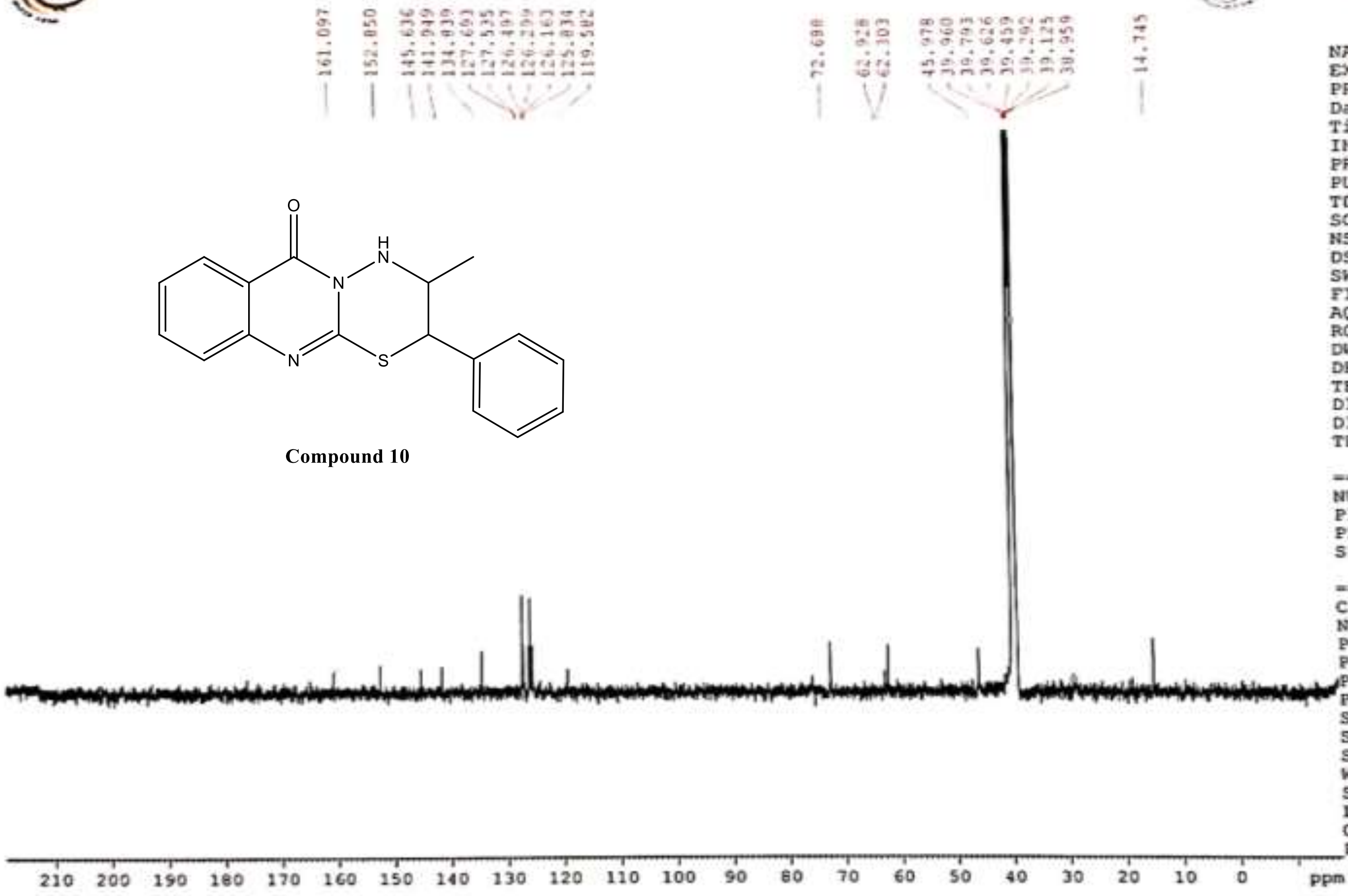

Compound 10

BRUKER

NAME	drqasoumi-18
EXPNO	21
PROCNO	1
Date_	20100609
Time	11.04
INSTRUM	spect
PROBHD	5 mm BBO BB-1H
PULPROG	zgpg30
TD	65536
SOLVENT	DMSO
NS	1024
DS	4
SWH	30030.029 Hz
FIDRES	0.458222 Hz
AQ	1.0912410 sec
RG	1625.5
DW	16.650 usec
DE	6.50 usec
TE	295.7 K
D1	2.00000000 sec
D11	0.03000000 sec
TD0	1

======== CHANNEL f1 ========

NUC1	13C
P1	5.80 usec
PL1	-2.00 dB
SFO1	125.7703643 MHz

======== CHANNEL f2 ========

CPDPRG2	waltz16
NUC2	1H
PCPD2	80.00 usec
PL2	-3.00 dB
PL12	14.64 dB
PL13	17.64 dB
SFO2	500.1320005 MHz
SI	32768
SF	125.7578519 MHz
WDW	EM
SSB	0
LB	3.00 Hz
GB	0
PC	1.40

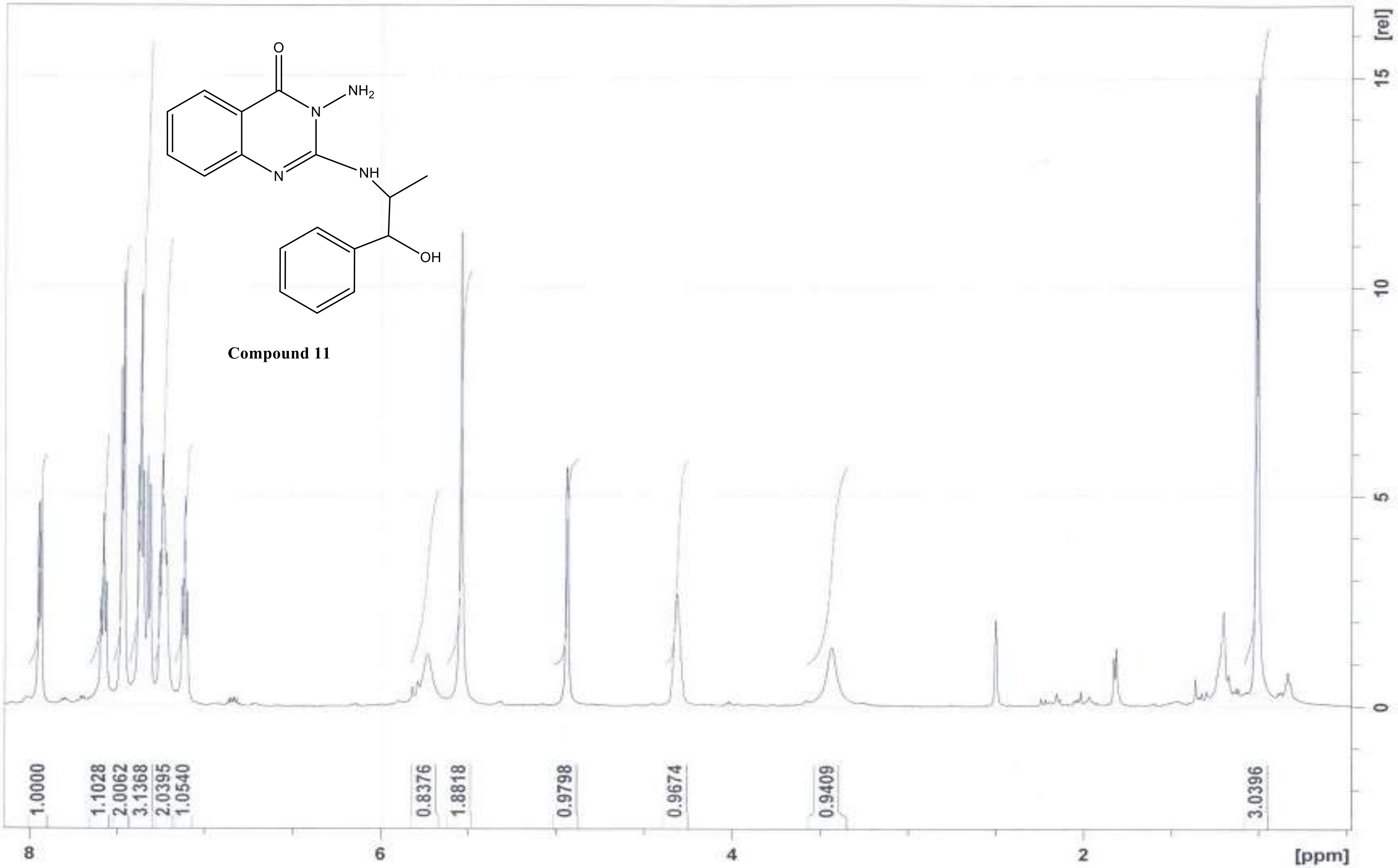
O
NH2
N
N
NH
OH
Compound 11
[rel]
15
10
5
0
1.0000
1.1028
2.0062
3.1368
2.0395
1.0540
0.8376
1.8818
0.9798
0.9674
0.9409
3.0396
8
6
4
2
[ppm]

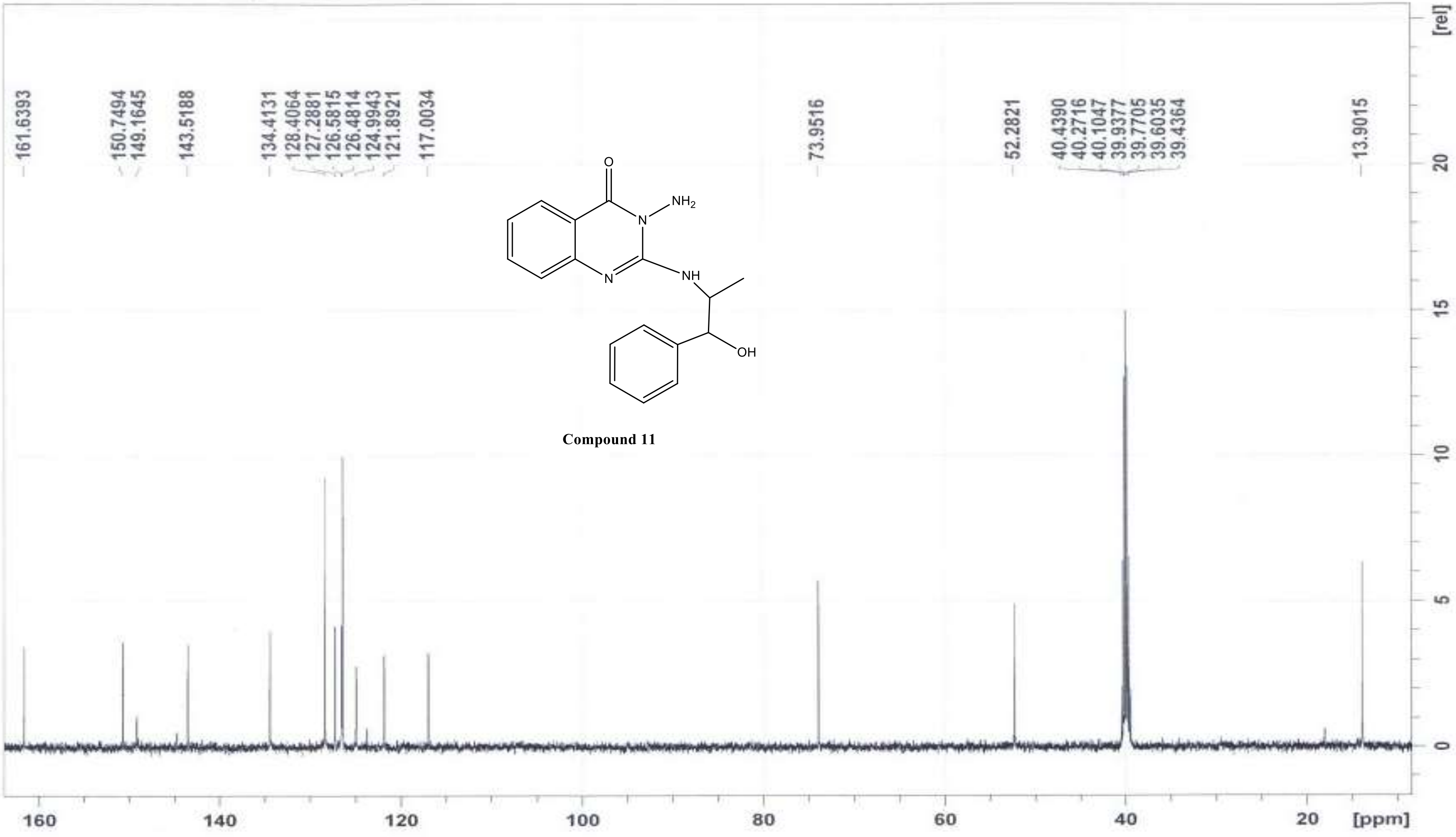
161.6393
150.7494
149.1645
143.5188
134.4131
128.4064
127.2881
126.5815
126.4814
124.9943
121.8921
117.0034
73.9516
52.2821
40.4390
40.2716
40.1047
39.9377
39.7705
39.6035
39.4364
13.9015
[rel]
[ppm]
O
NH2
N
N
NH
OH

Compound 11

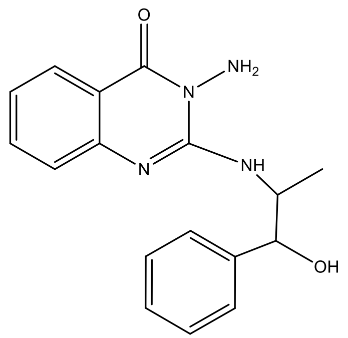

Compound 11

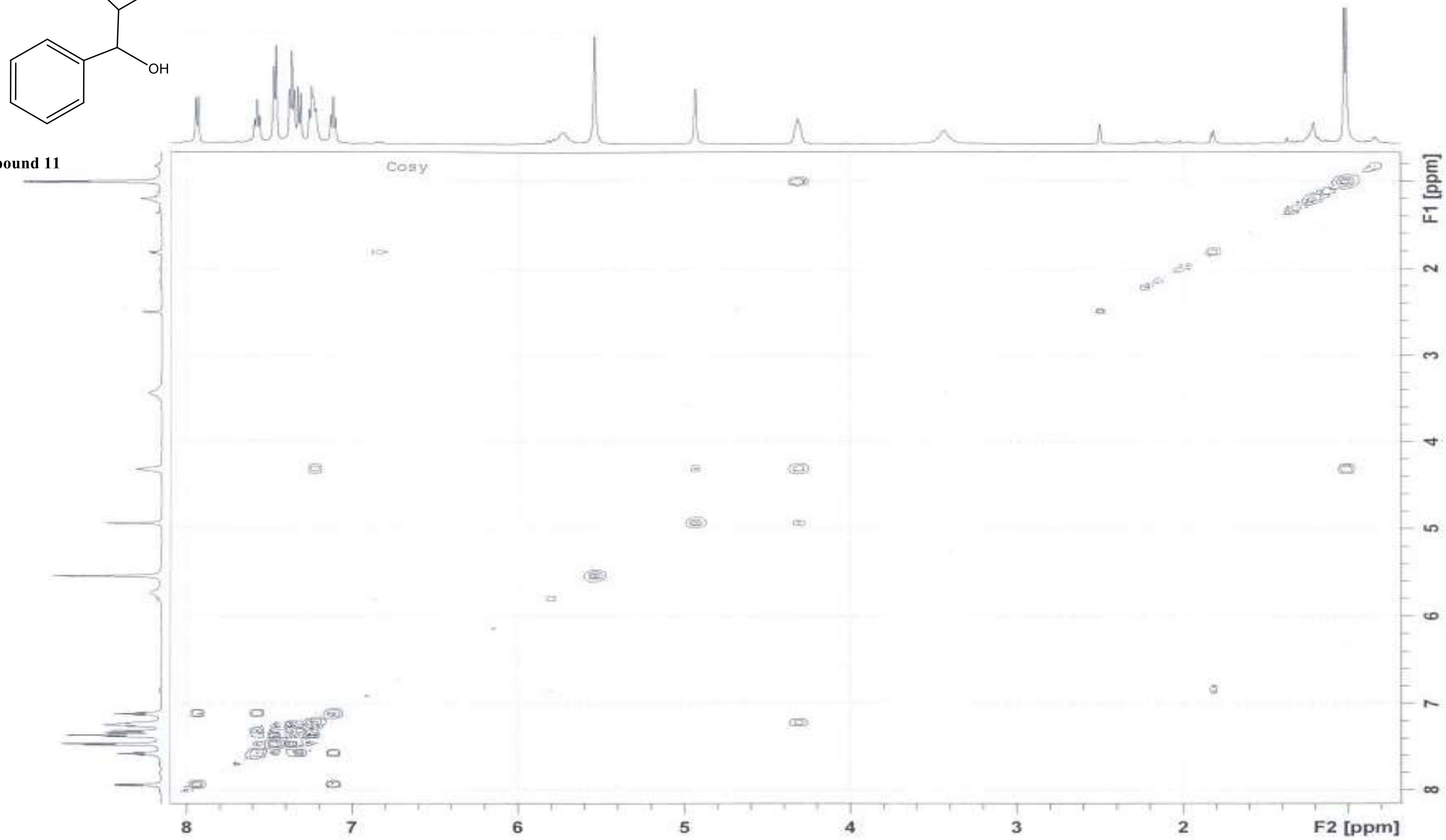

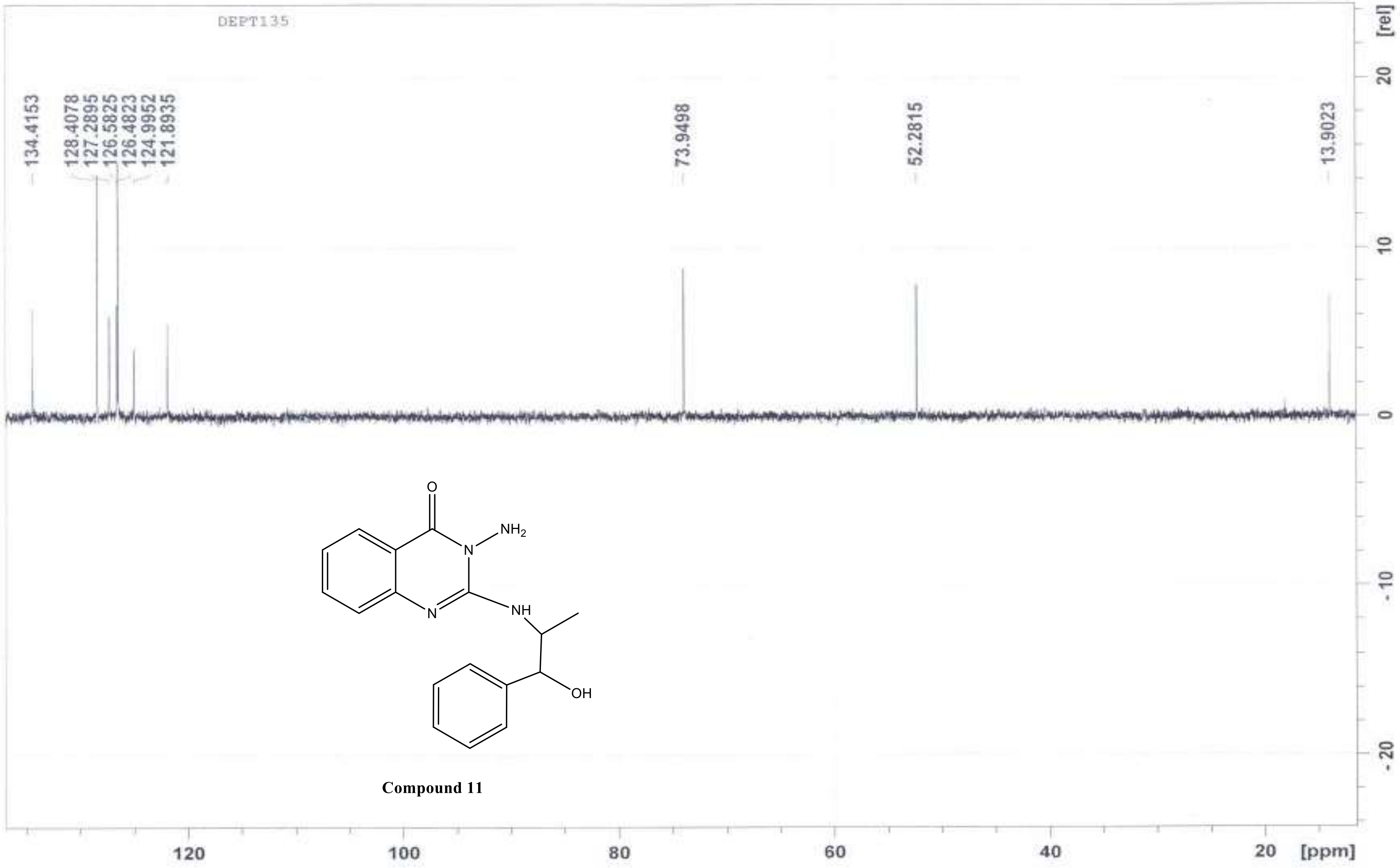
DEPT135
134.4153
128.4078
127.2895
126.5825
126.4823
124.9952
121.8935
73.9498
52.2815
13.9023
[rel]
20
10
0
-10
-20
120
100
80
60
40
20
[ppm]
O
NH2
N
N
NH
OH

Compound 11

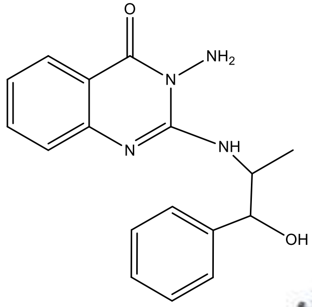

Compound 11

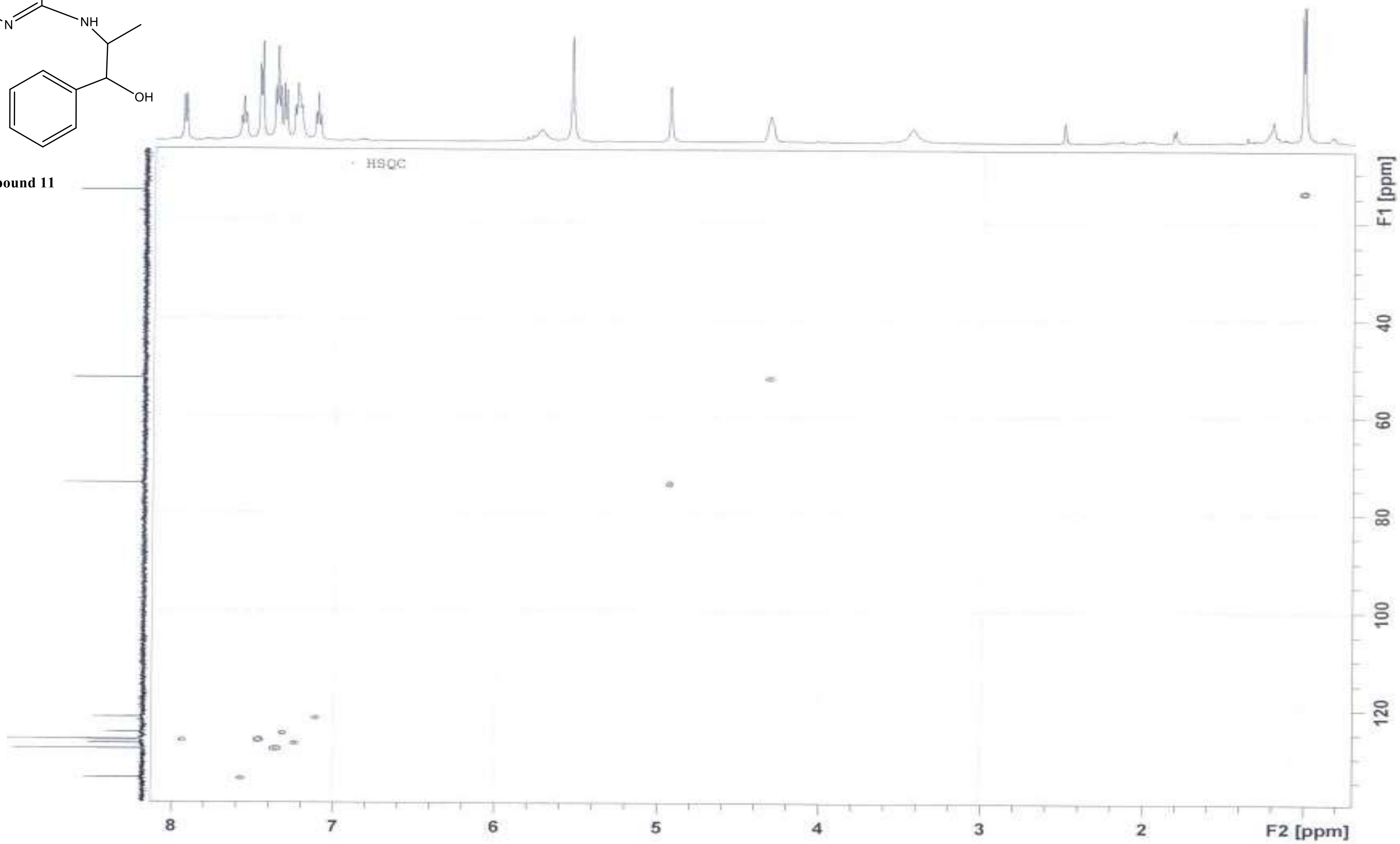

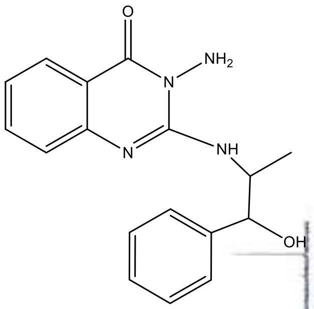

Compound 11

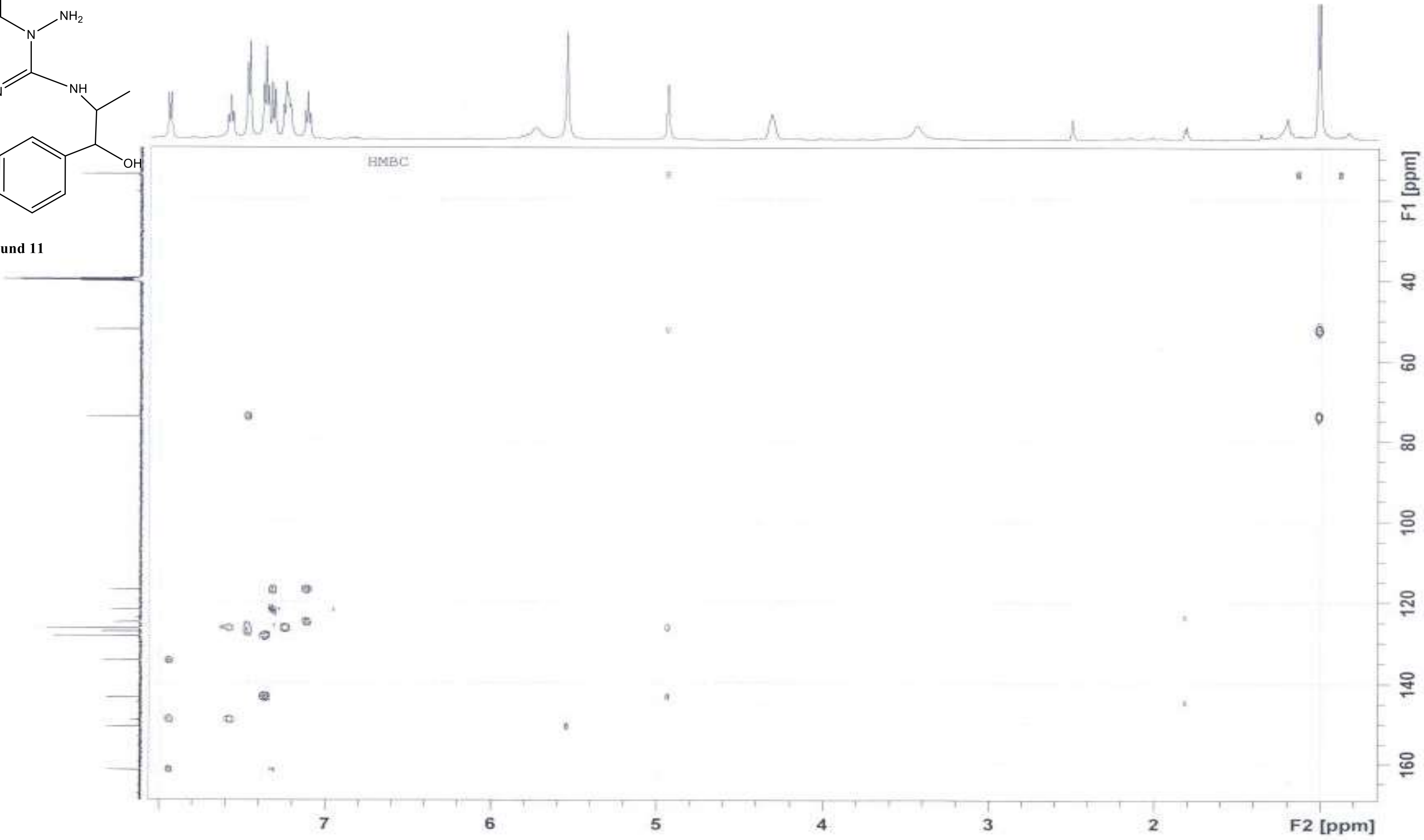

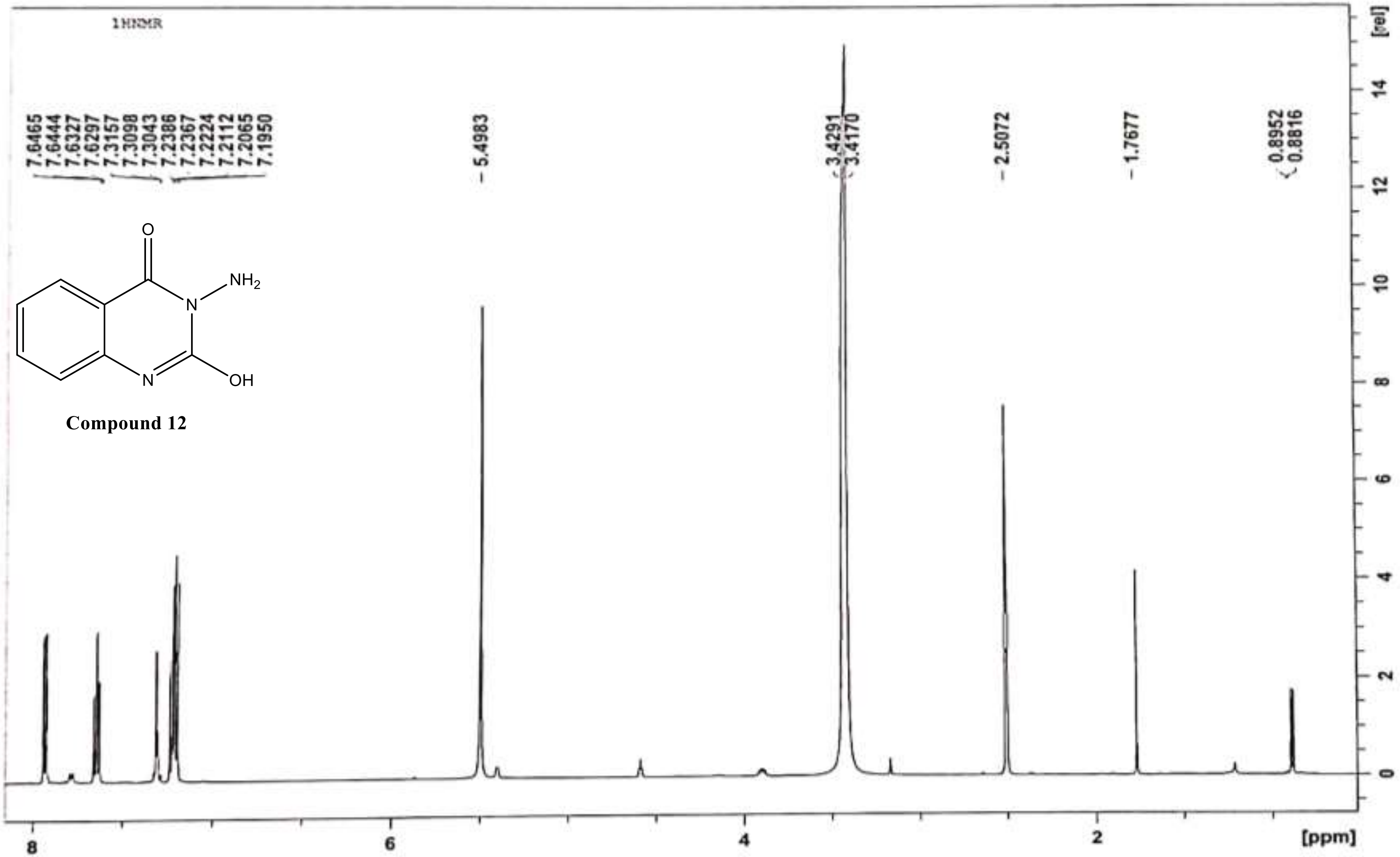
1HNMR
7.6465
7.6444
7.6327
7.6297
7.3157
7.3098
7.3043
7.2386
7.2367
7.2224
7.2112
7.2065
7.1950
5.4983
3.4291
3.4170
2.5072
1.7677
0.8952
0.8816
O
NH2
N
N
OH
[rel]
[ppm]
8
6
4
2
0
2
4
6
8
10
12
14

Compound 12

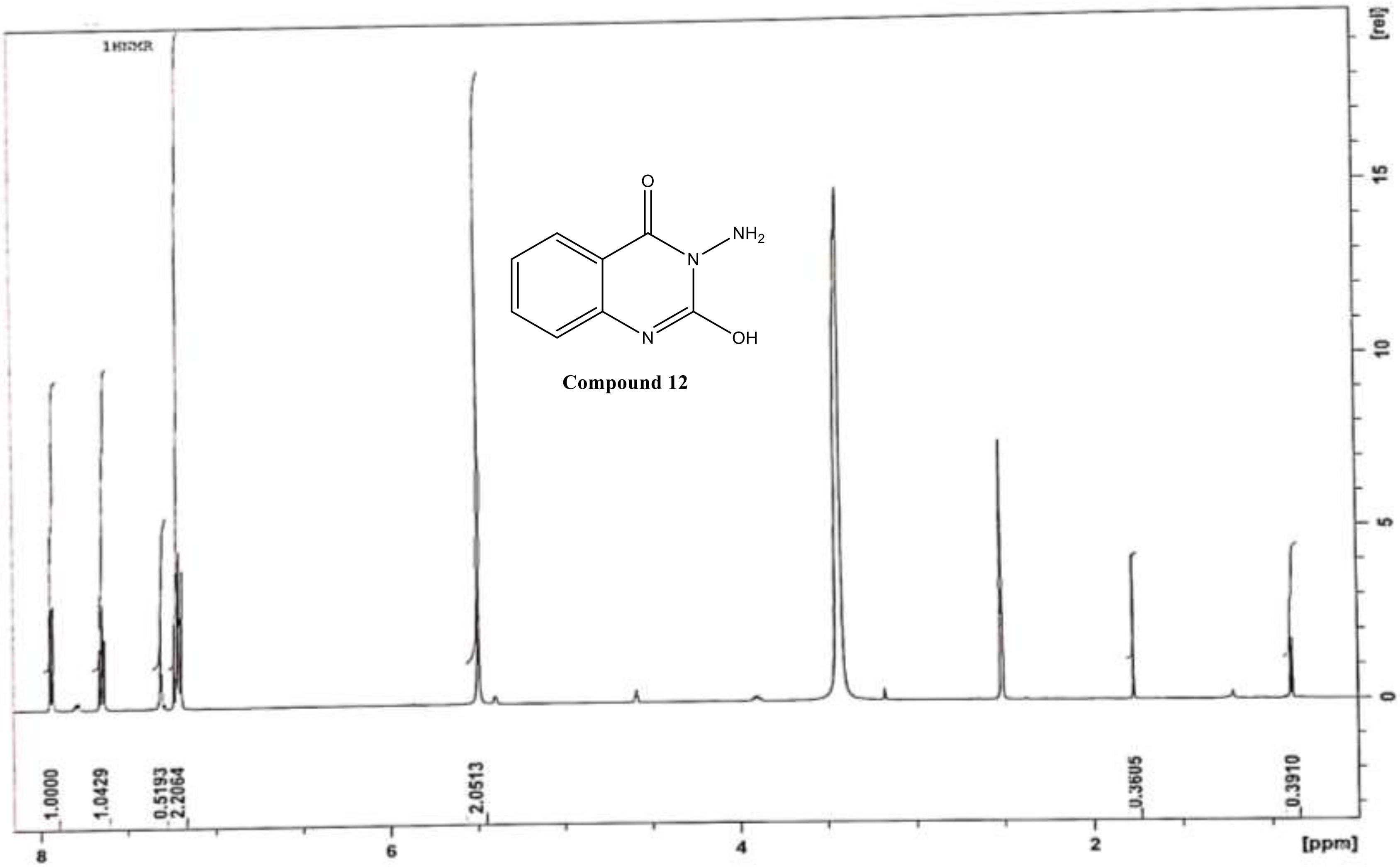
1HNMR
O
NH2
N
N
OH
Compound 12
1.0000
1.0429
0.5193
2.2064
2.0513
0.3605
0.3910
8
6
4
2
[ppm]
[rel]
15
10
5
0

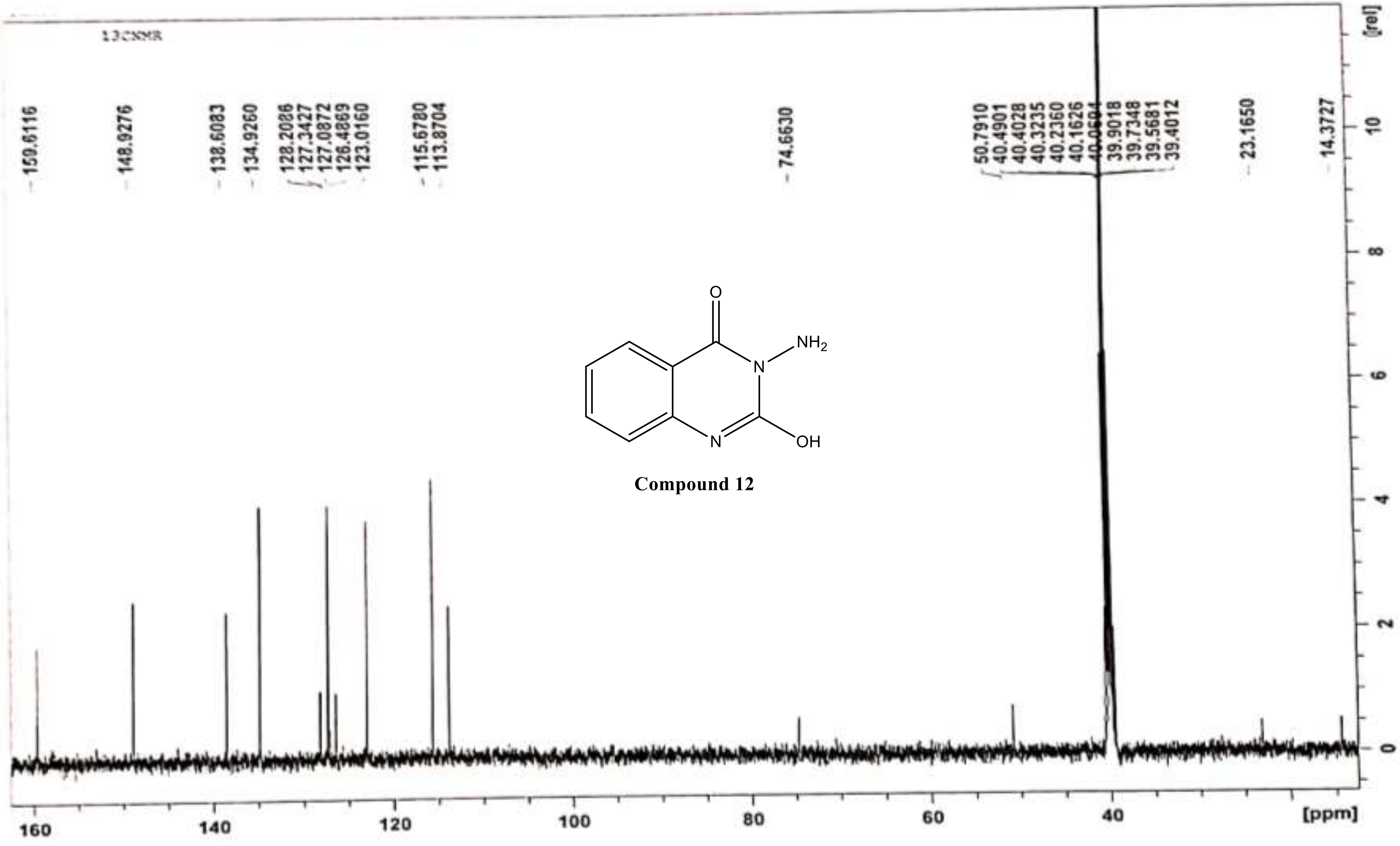
13CNMR
159.6116
148.9276
138.6083
134.9260
128.2086
127.3427
127.0872
126.4869
123.0160
115.6780
113.8704
74.6630
50.7910
40.4901
40.4028
40.3235
40.2360
40.1626
40.0691
39.9018
39.7348
39.5681
39.4012
23.1650
14.3727
O
NH2
N
N
OH
[rel]
[ppm]

Compound 12

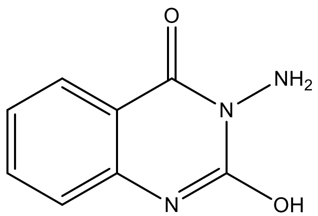

Compound 12

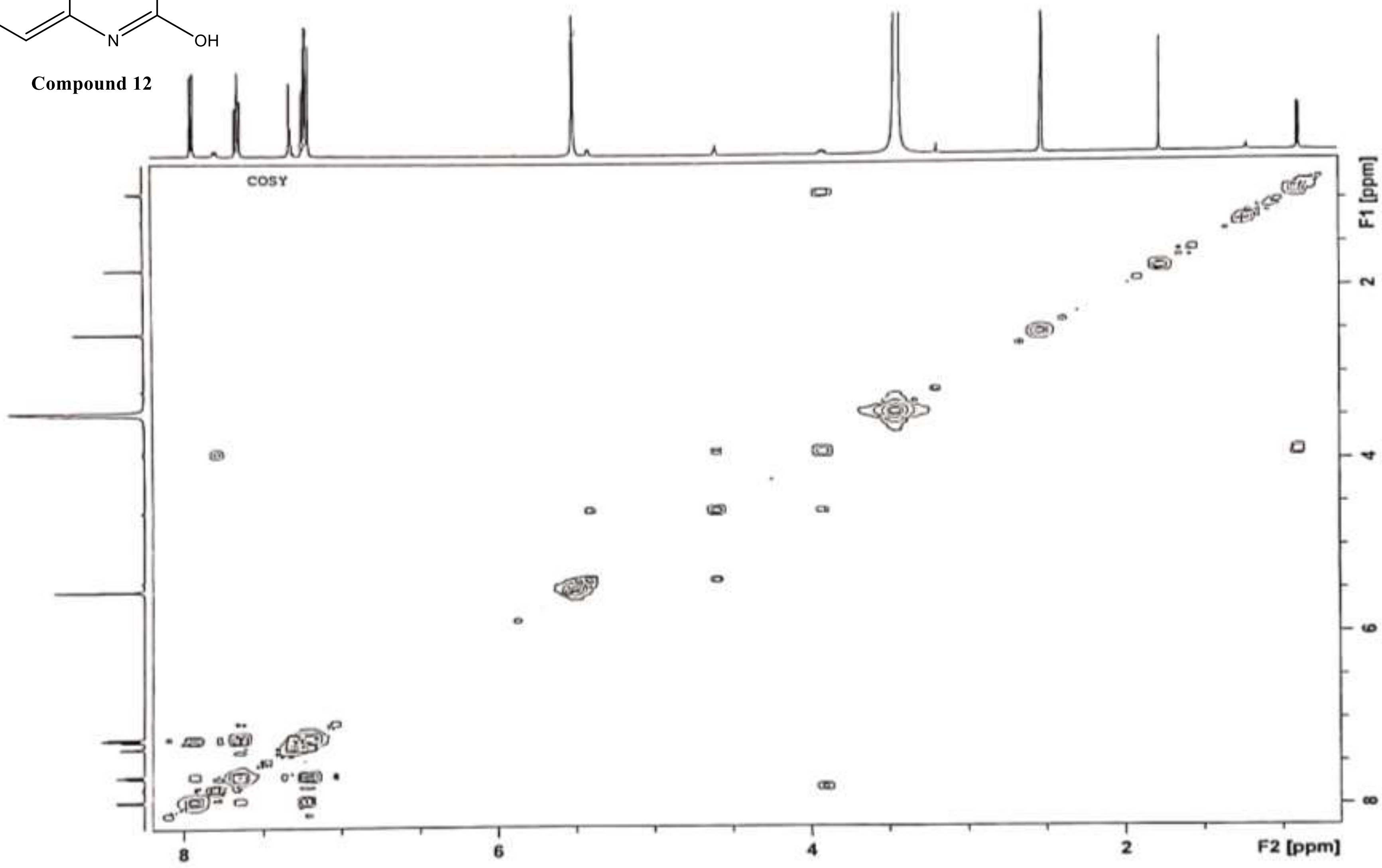

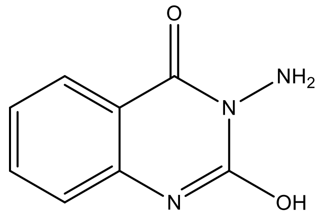

Compound 12

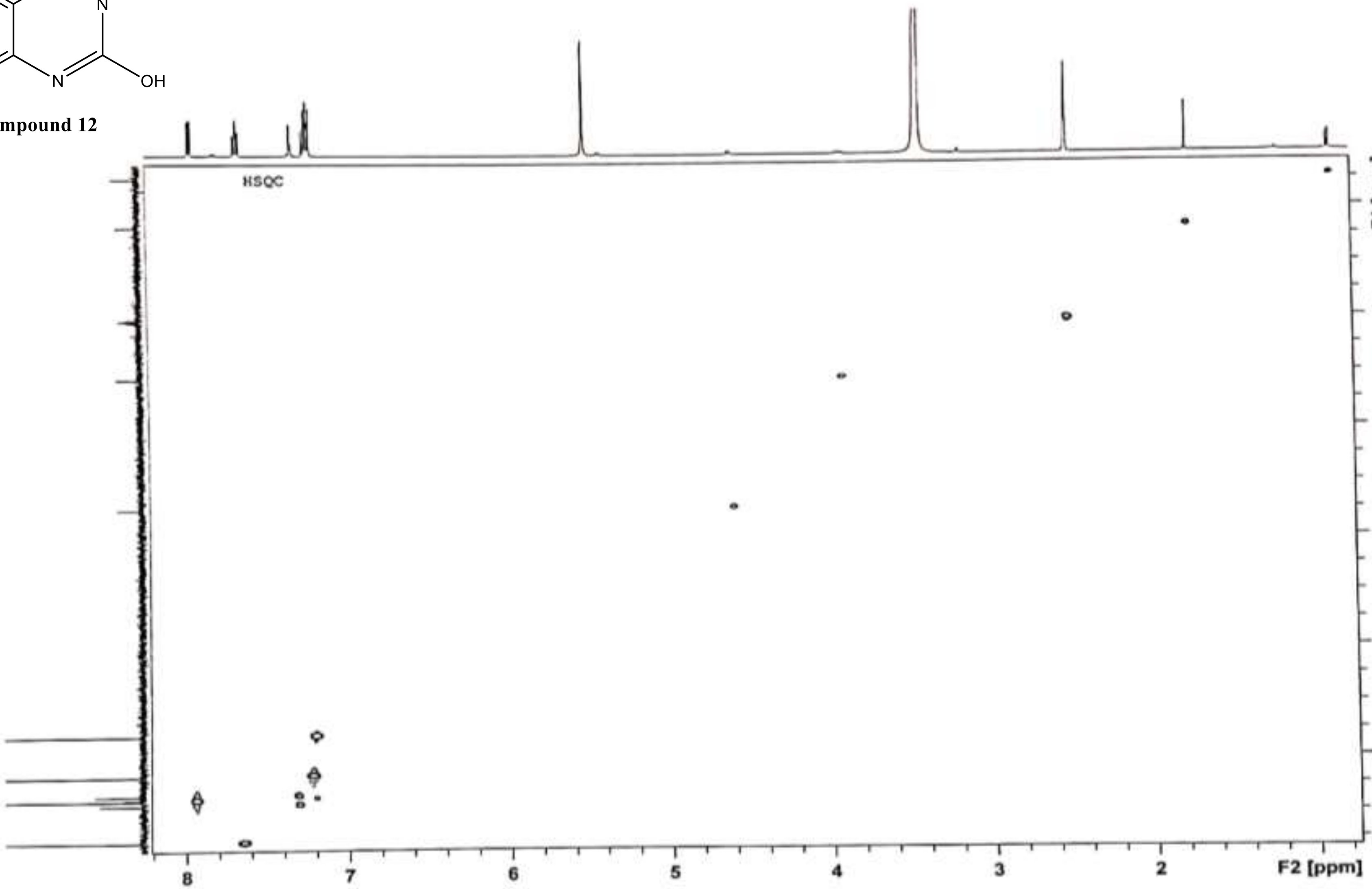

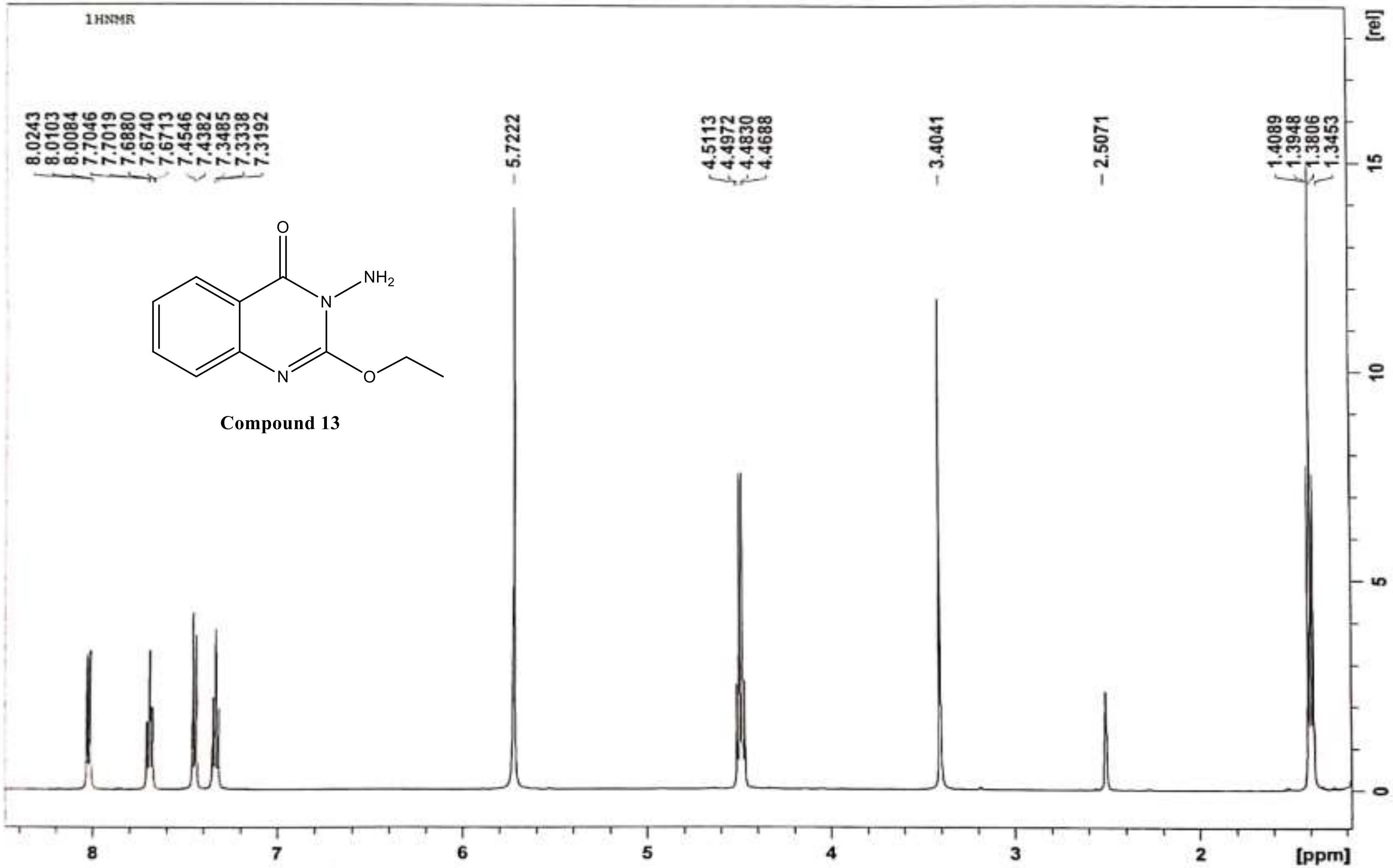
1HNMR
8.0243
8.0103
8.0084
7.7046
7.7019
7.6880
7.6740
7.6713
7.4546
7.4382
7.3485
7.3338
7.3192
5.7222
4.5113
4.4972
4.4830
4.4688
3.4041
2.5071
1.4089
1.3948
1.3806
1.3453
O
NH2
N
N
O
[rel]
15
10
5
0
8
7
6
5
4
3
2
[ppm]

Compound 13

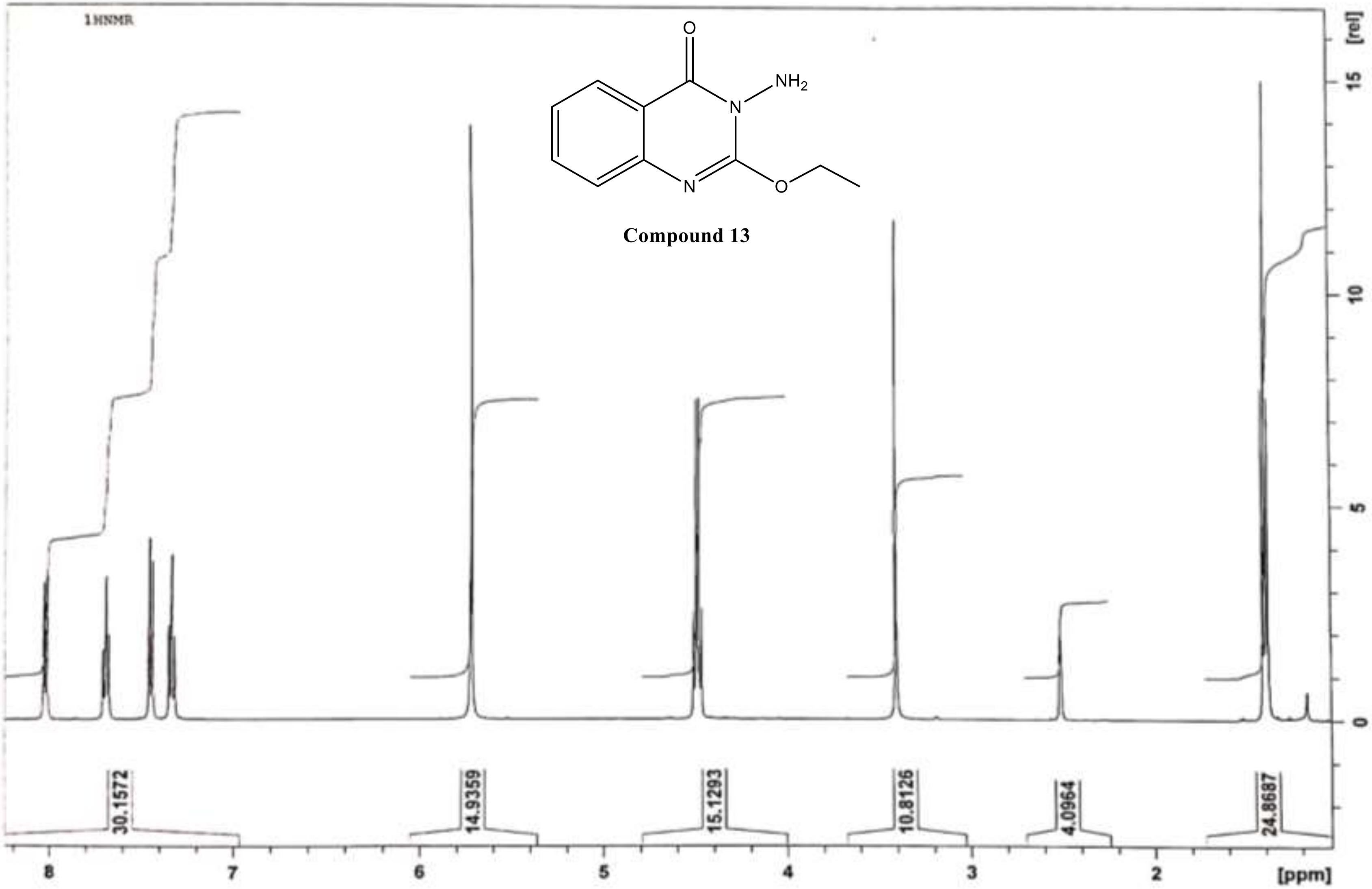
1HNMR
NH2
N
O
[rel]
[ppm]
30.1572
14.9359
15.1293
10.8126
4.0964
24.8687

Compound 13

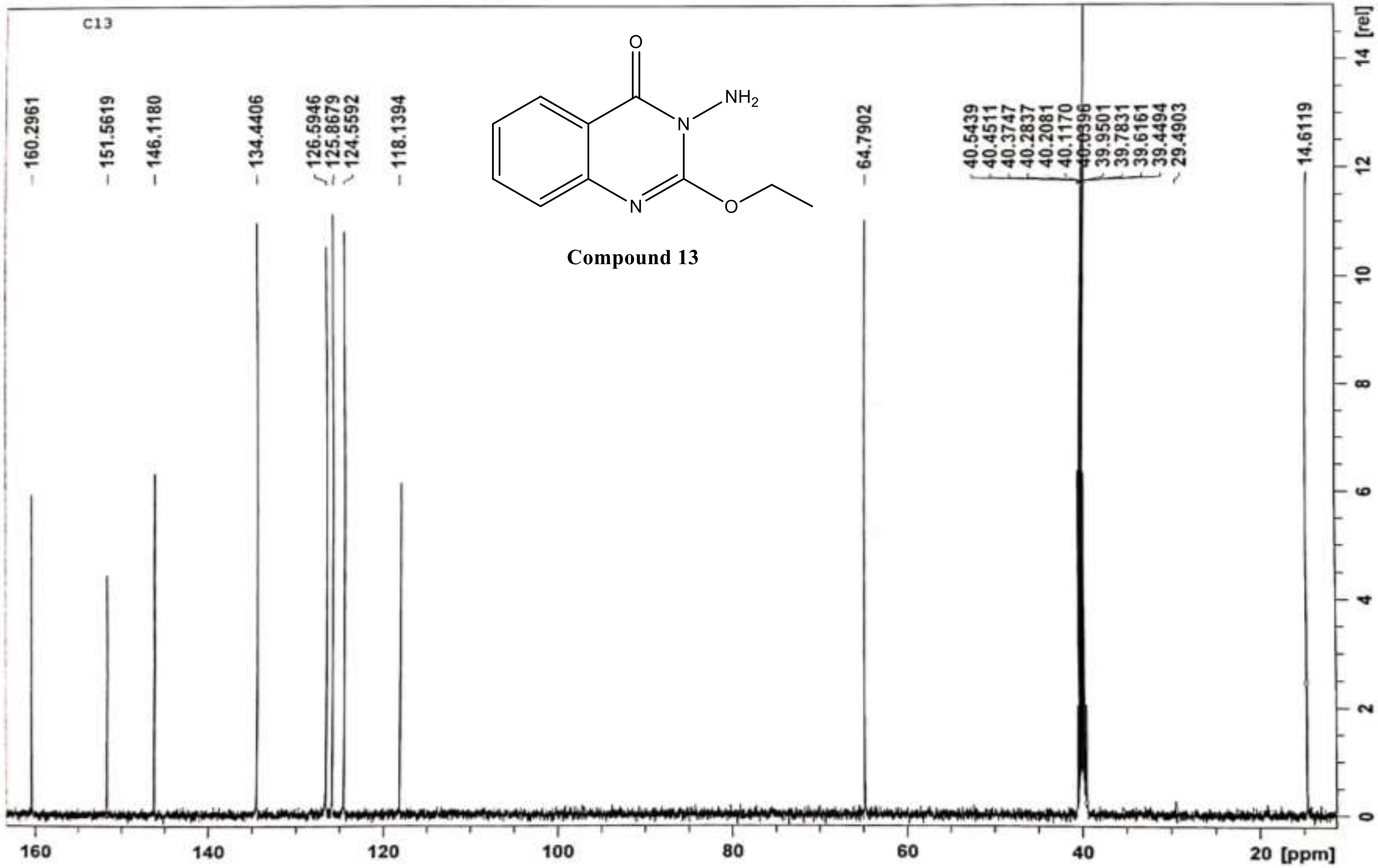
C13
160.2961
151.5619
146.1180
134.4406
126.5946
125.8679
124.5592
118.1394
64.7902
40.5439
40.4511
40.3747
40.2837
40.2081
40.1170
40.0396
39.9501
39.7831
39.6161
39.4494
29.4903
14.6119
O
N
NH2
N
O
[rel]
[ppm]

Compound 13

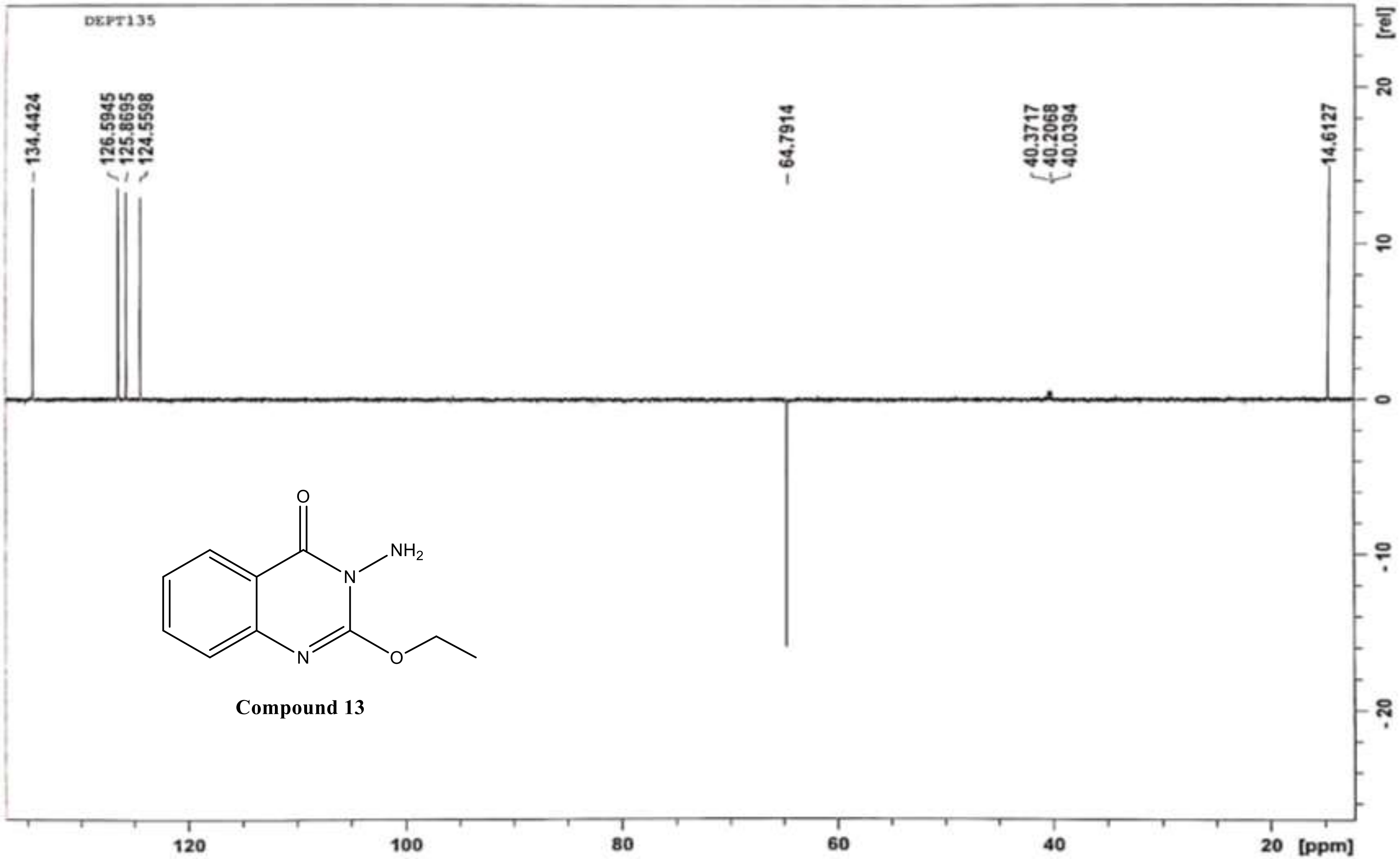
DEPT135
134.4424
126.5945
125.8695
124.5598
64.7914
40.3717
40.2068
40.0394
14.6127
[rel]
20
10
0
-10
-20
120
100
80
60
40
20 [ppm]
O
NH2
N
N
O
Compound 13

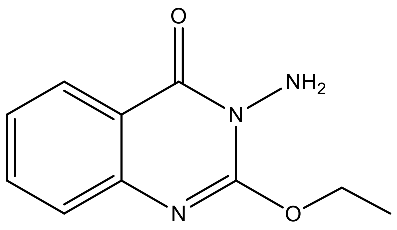

Compound 13

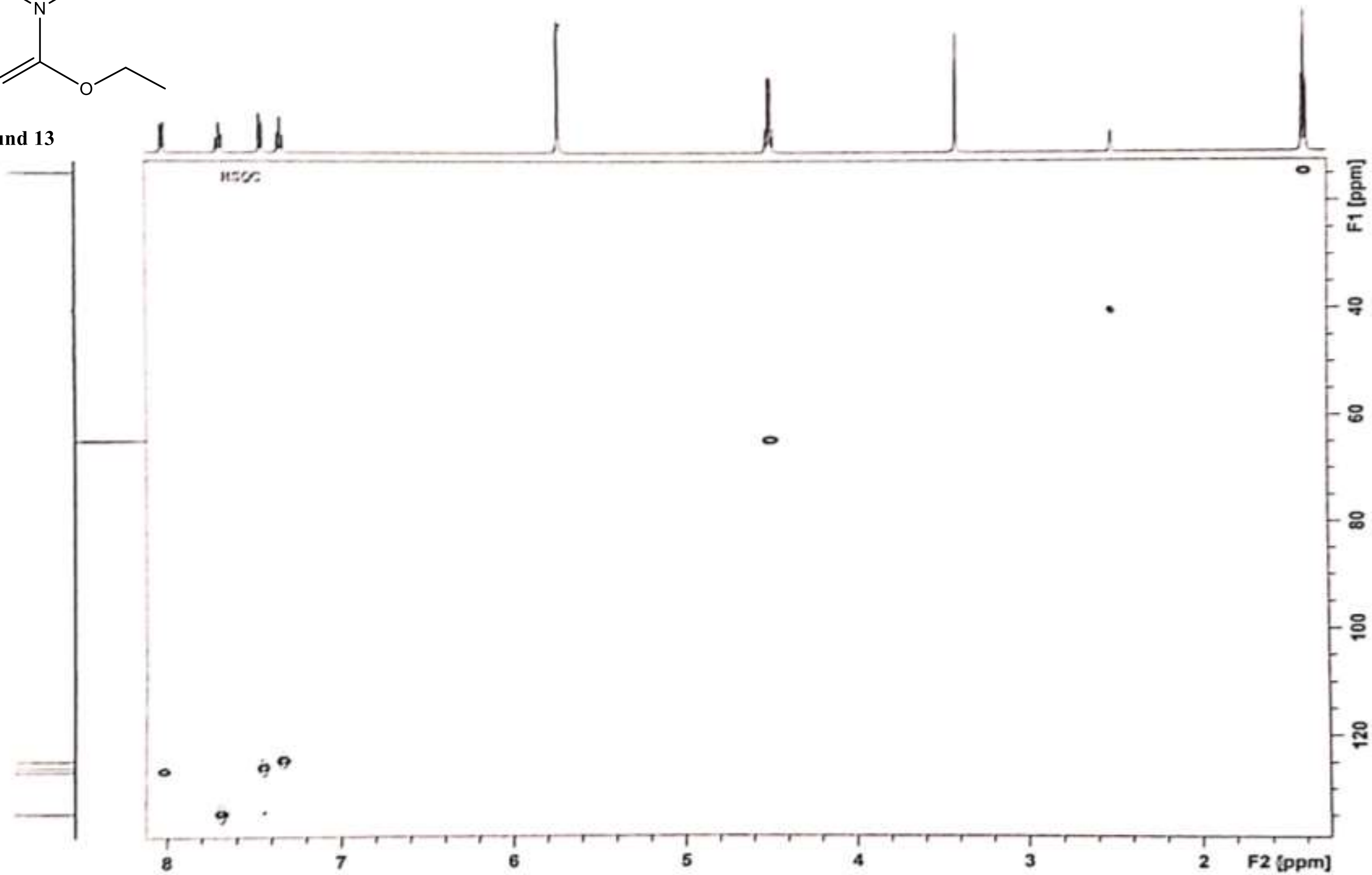

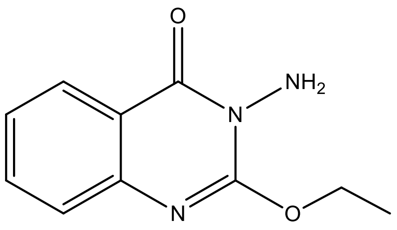

Compound 13

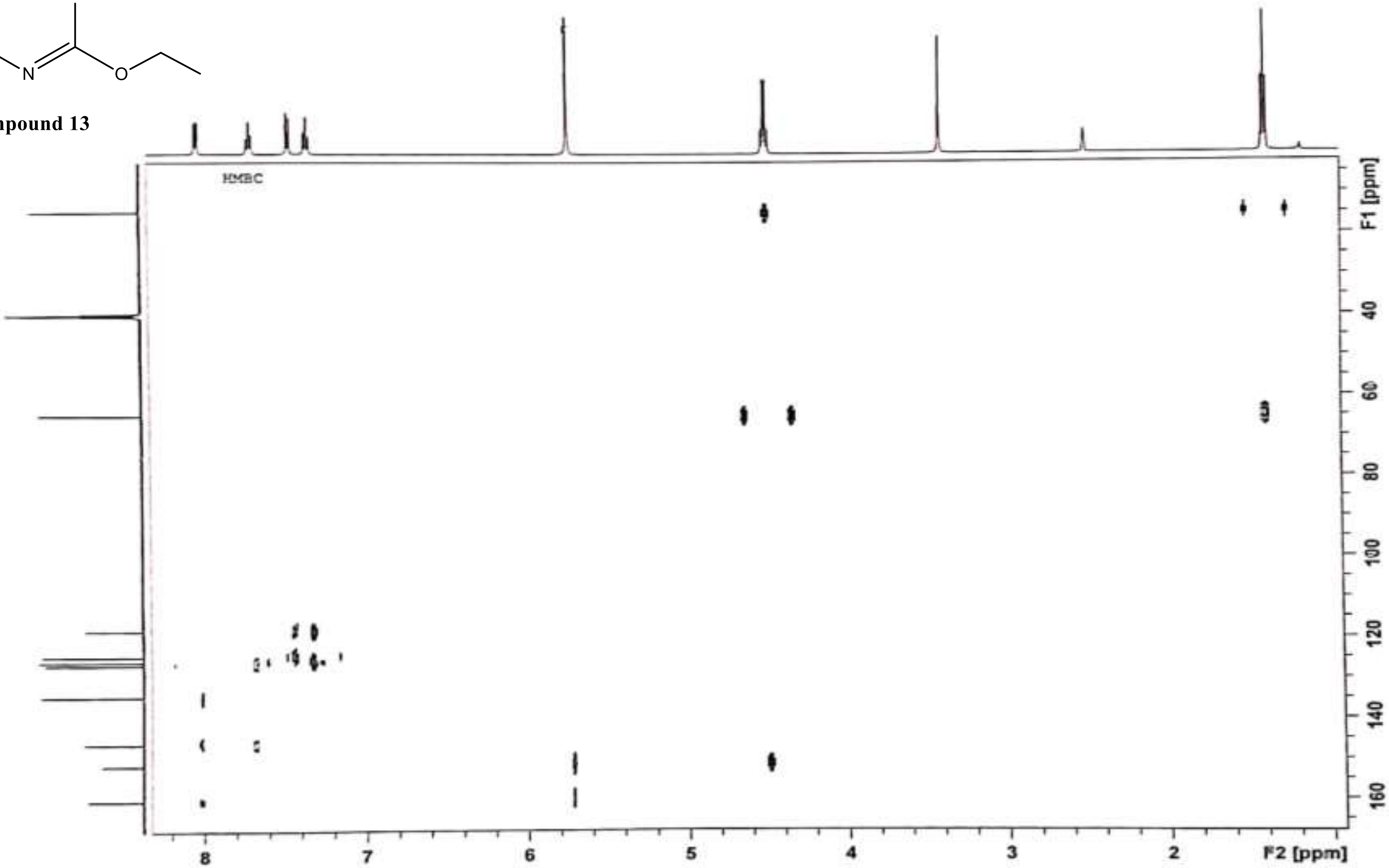

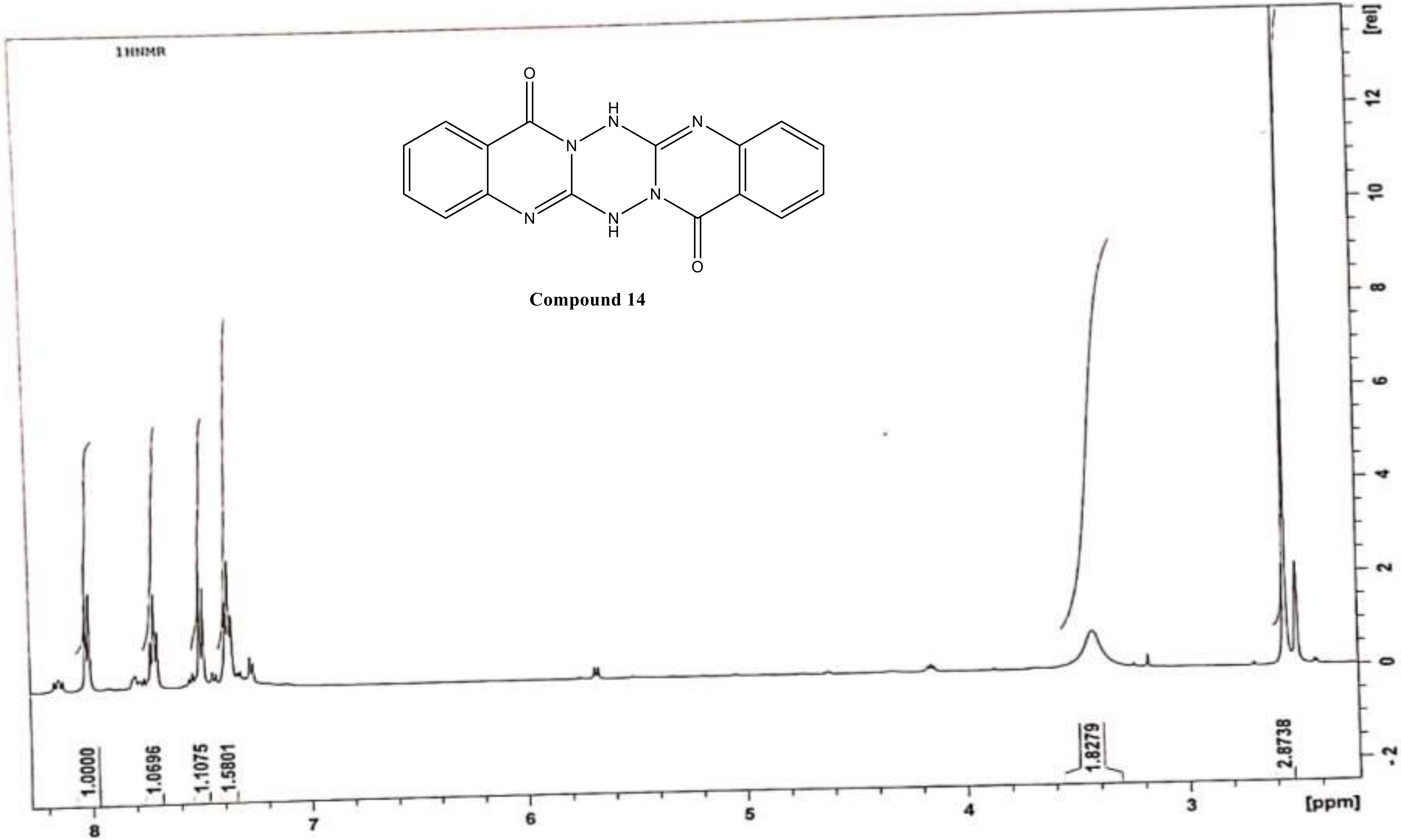
1HNMR
Compound 14
1.0000
1.0696
1.1075
1.5801
1.8279
2.8738
[ppm]
[rel]

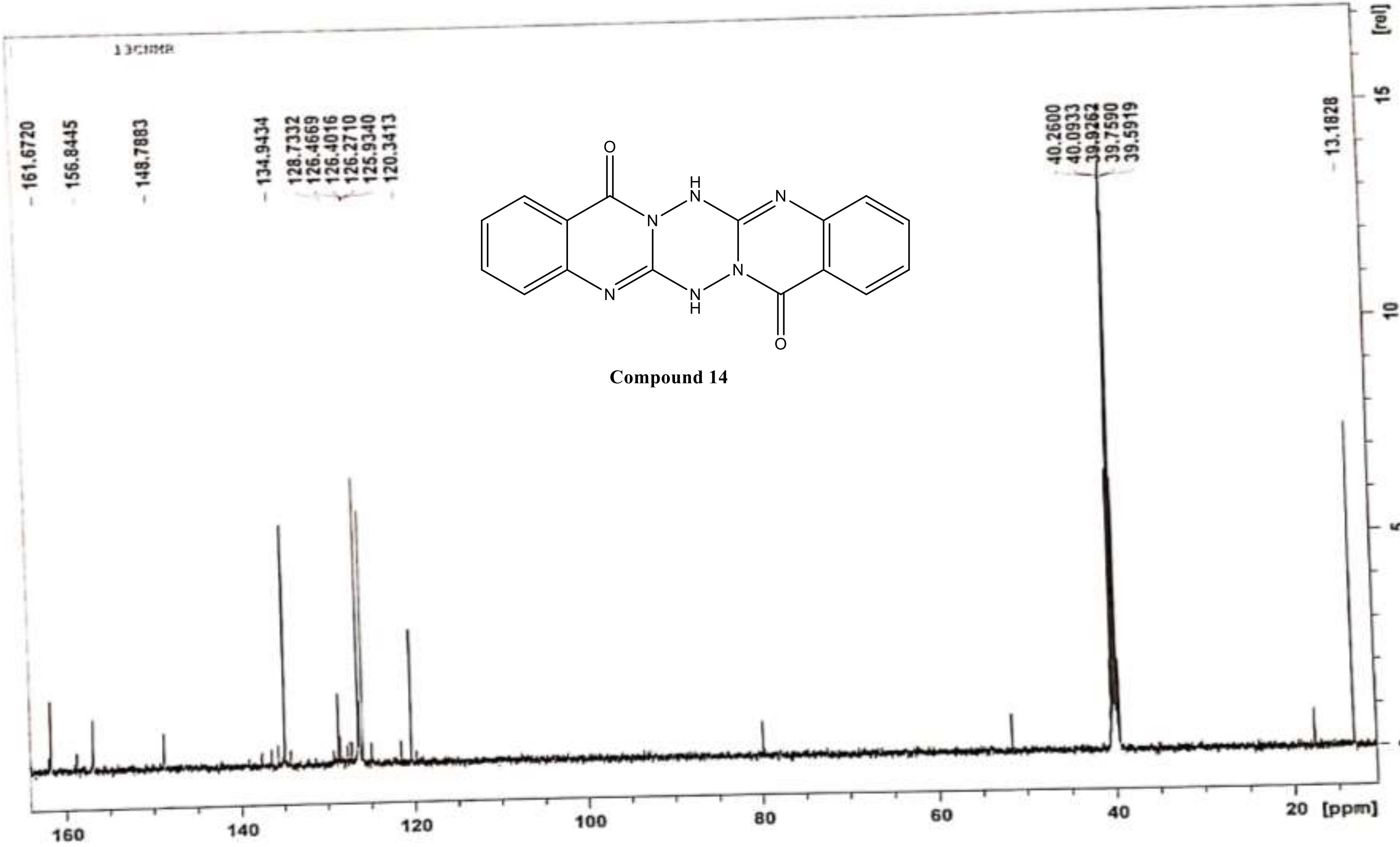

13CNMR
161.6720
156.8445
148.7883
134.9434
128.7332
126.4669
126.4016
126.2710
125.9340
120.3413
40.2600
40.0933
39.9262
39.7590
39.5919
13.1828
[rel]
[ppm]

Compound 14

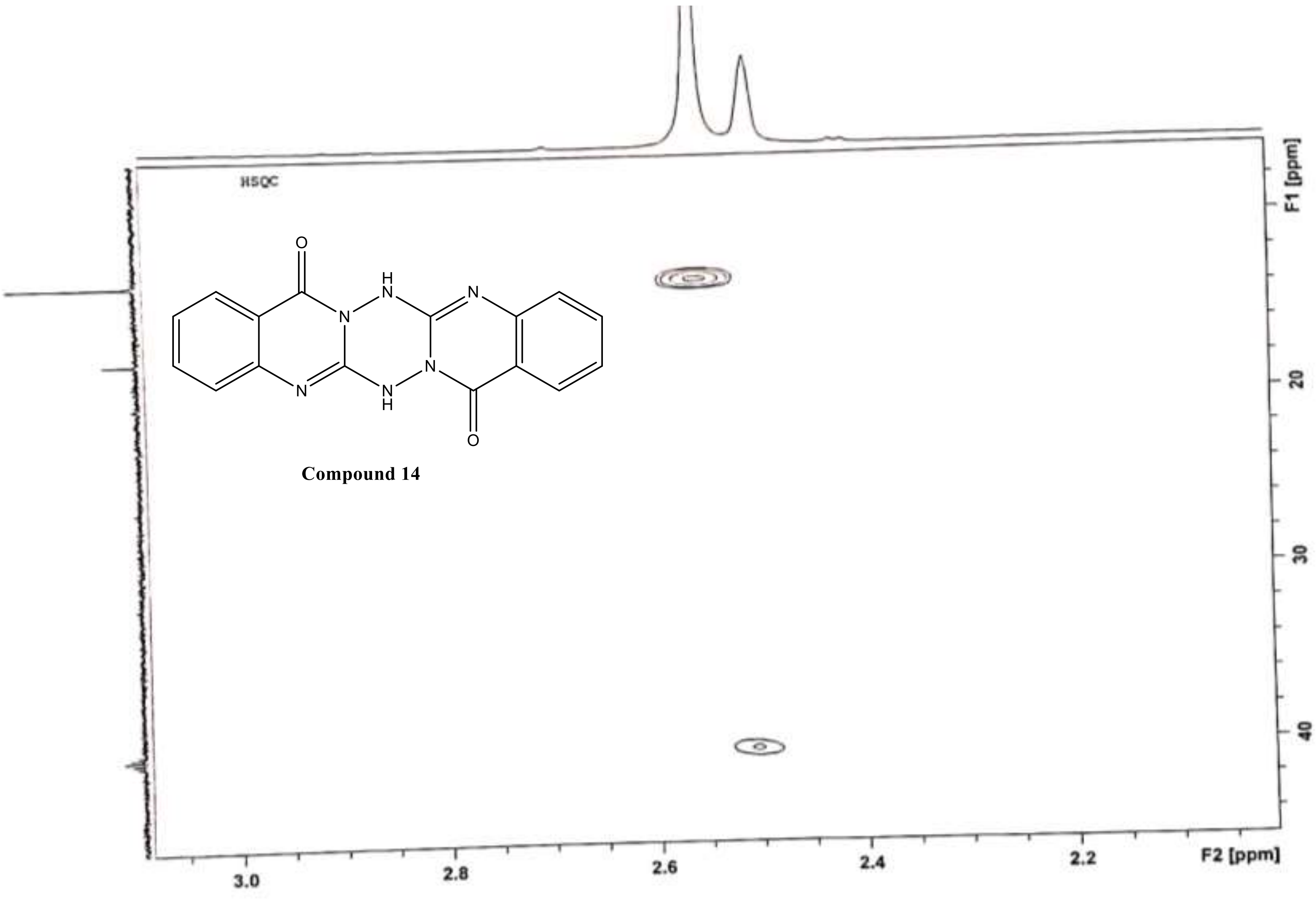

Compound 14

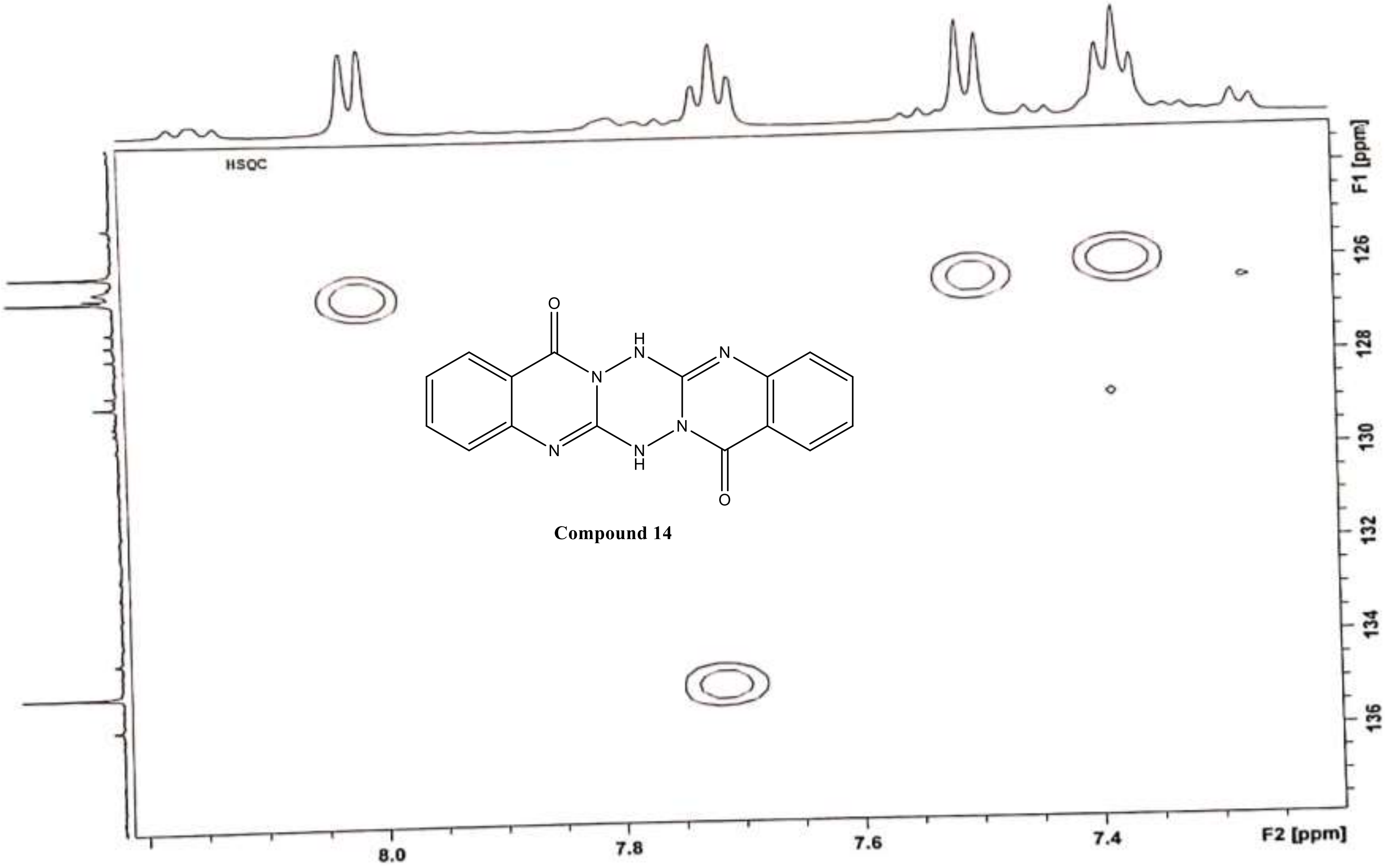
HSQC
F1 [ppm]
126
128
130
132
134
136
8.0
7.8
7.6
7.4
F2 [ppm]
O
H
N
N
N
N
N
N
H
O

Compound 14

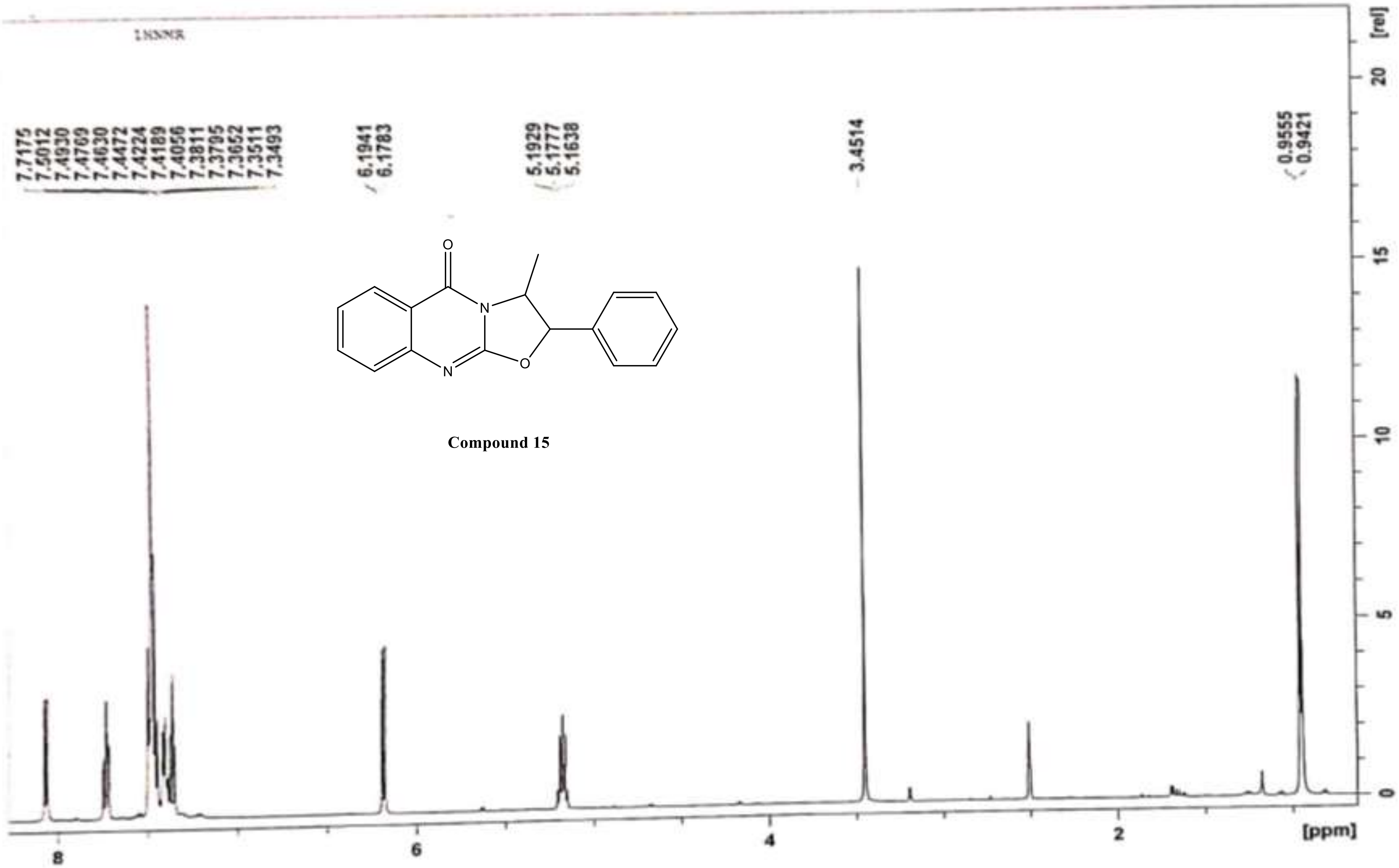

7.7175
7.5012
7.4930
7.4769
7.4630
7.4472
7.4224
7.4189
7.4056
7.3811
7.3795
7.3652
7.3511
7.3493
6.1941
6.1783
5.1929
5.1777
5.1638
3.4514
0.9555
0.9421
[rel]
[ppm]

Compound 15

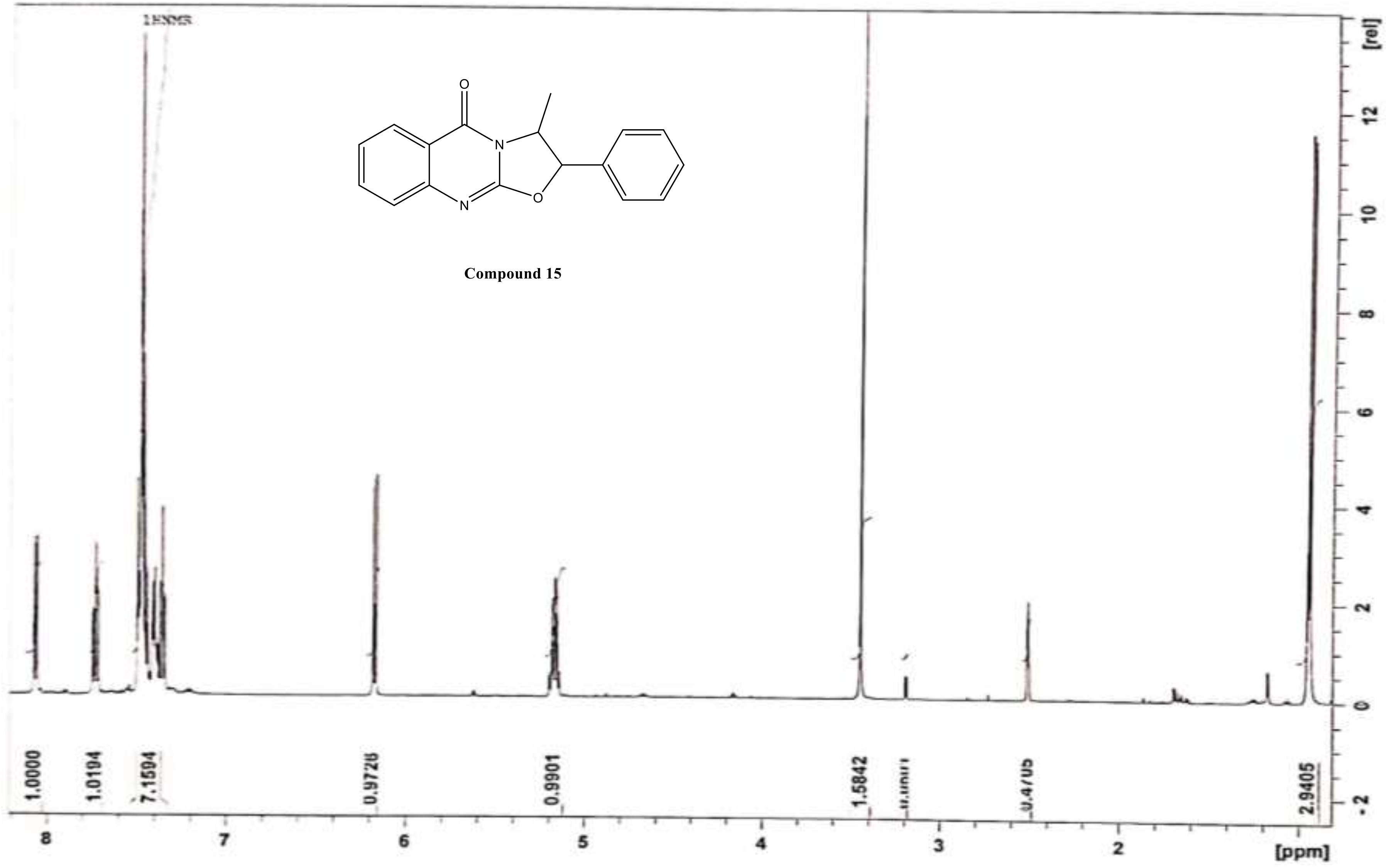
Compound 15
[rel]
1.0000
1.0194
7.1594
0.9726
0.9901
1.5842
2.9405
[ppm]

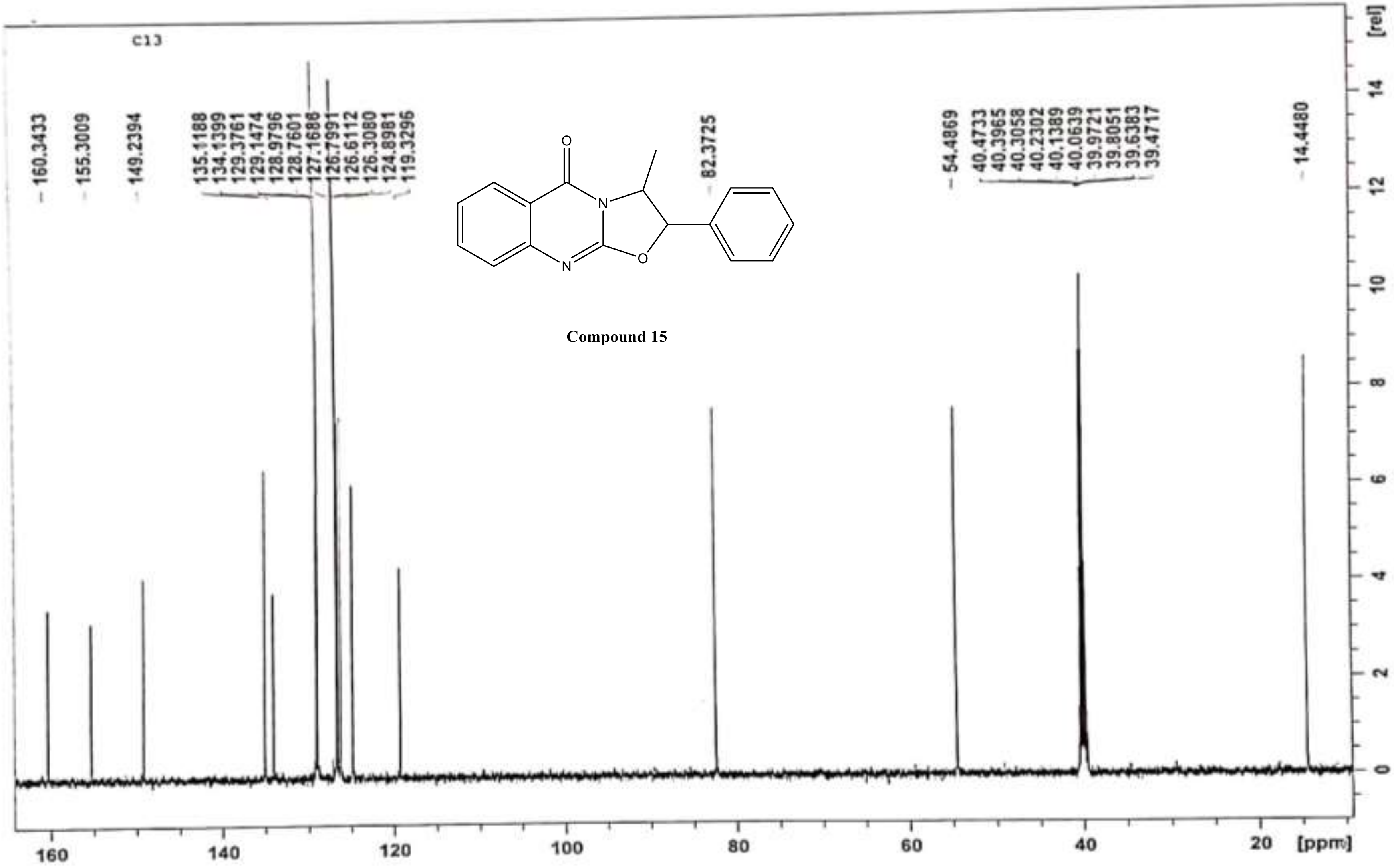
C13
160.3433
155.3009
149.2394
135.1188
134.1399
129.3761
129.1474
128.9796
128.7601
127.1686
126.7991
126.6112
126.3080
124.8981
119.3296
82.3725
54.4869
40.4733
40.3965
40.3058
40.2302
40.1389
40.0639
39.9721
39.8051
39.6383
39.4717
14.4480
O
N
N
O
[rel]
[ppm]

Compound 15

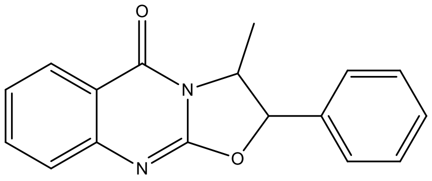

Compound 15

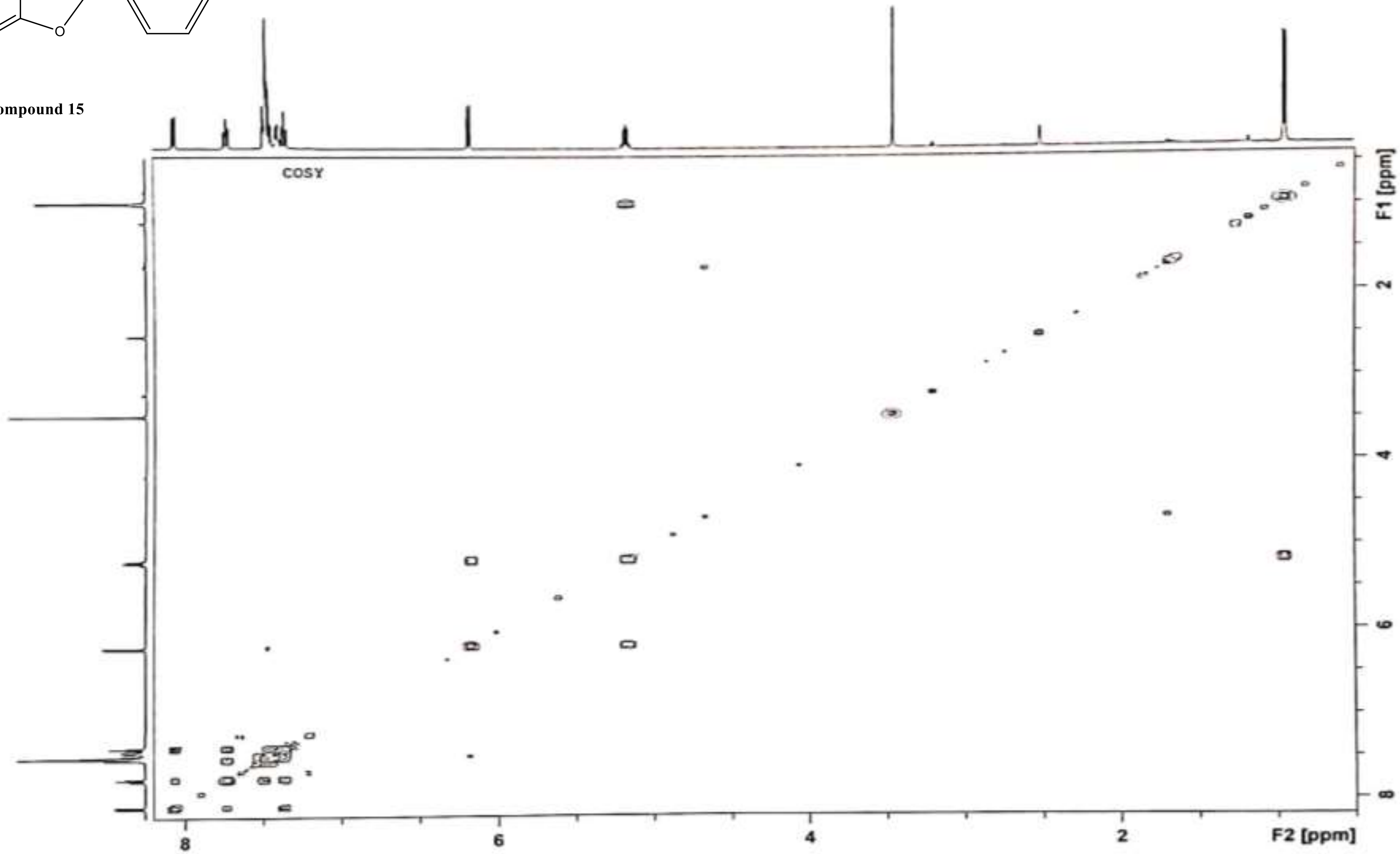

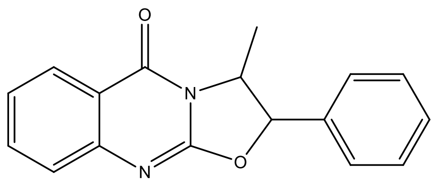

Compound 15

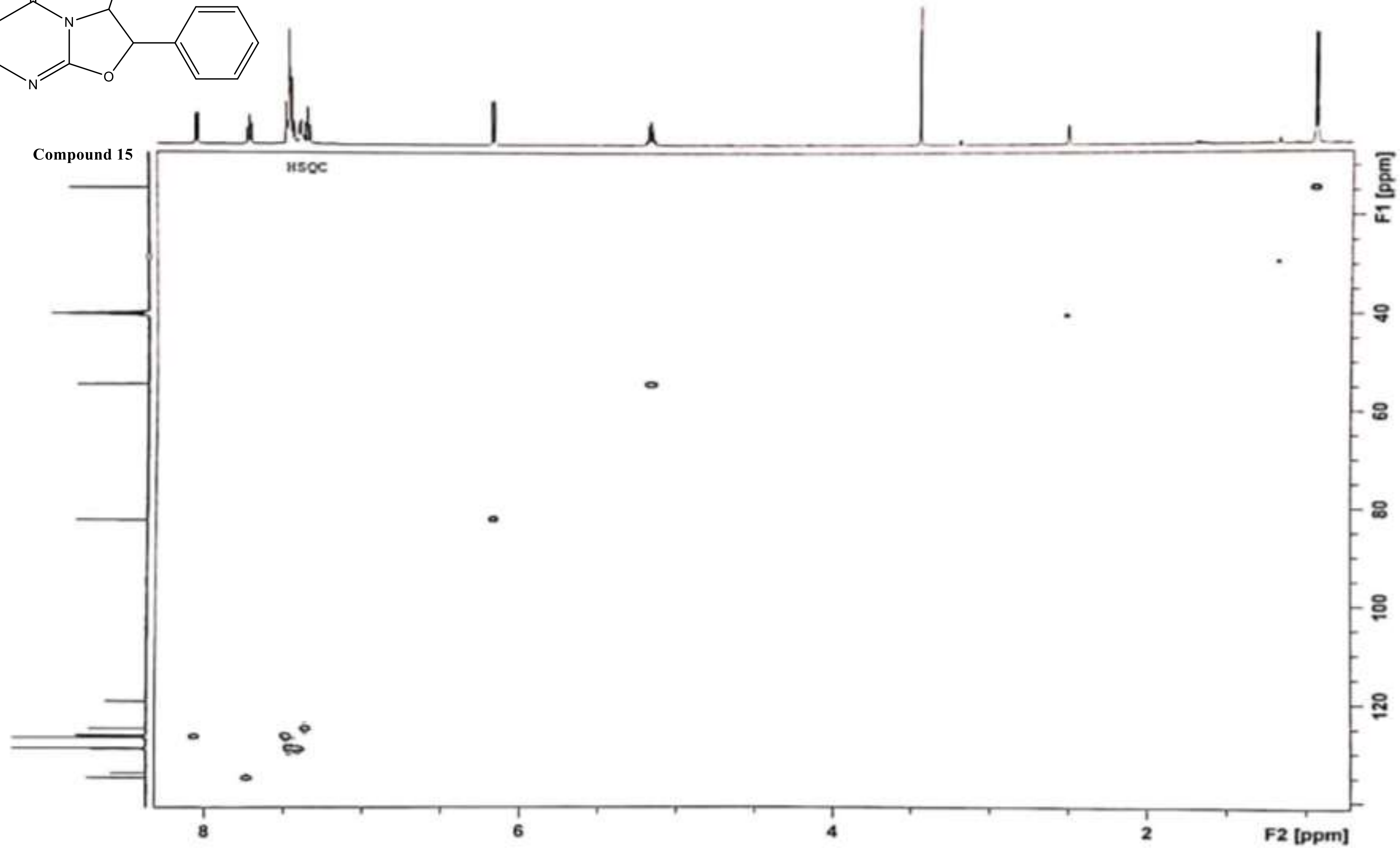

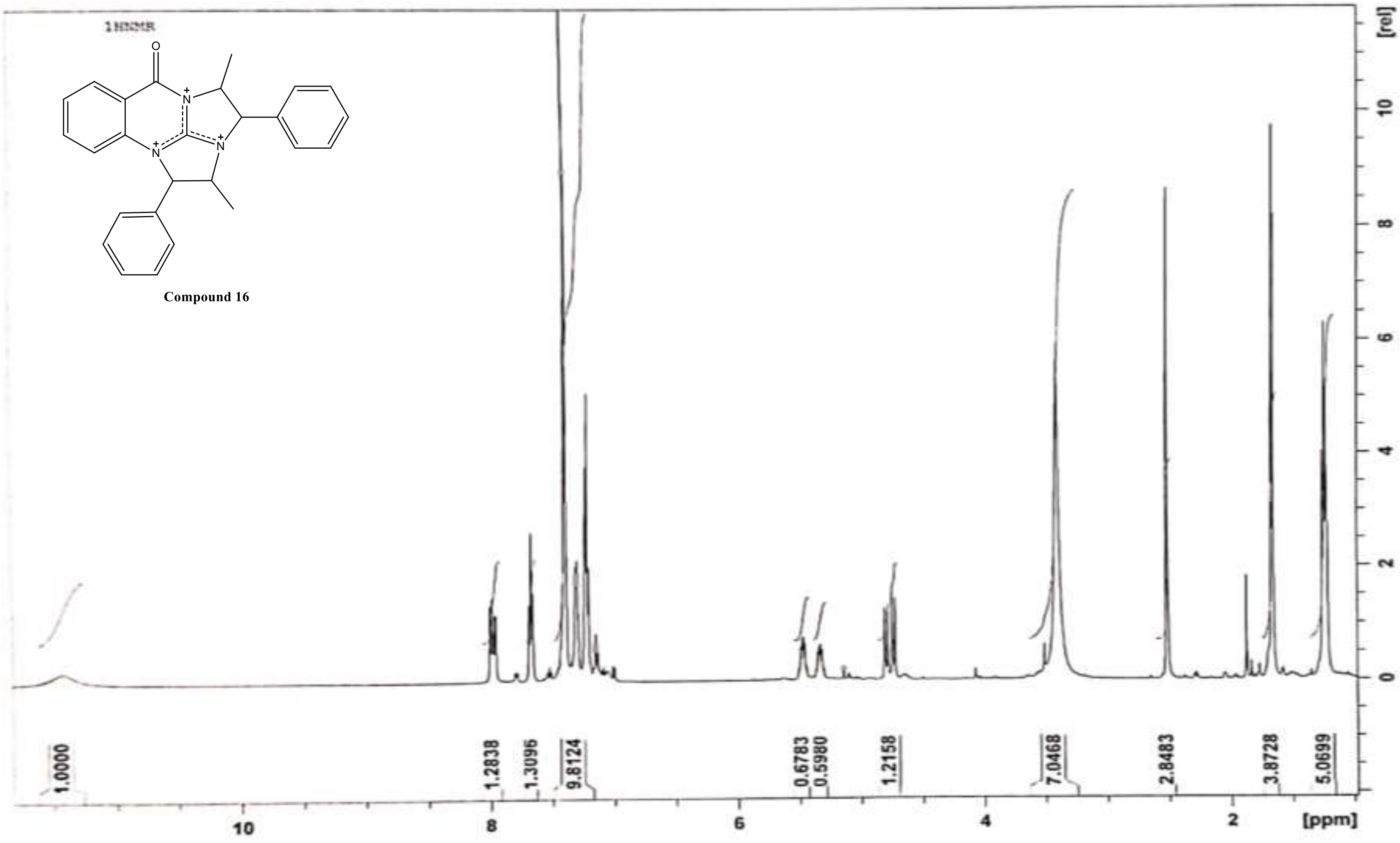
1HNMR
Compound 16
[rel]
10
8
6
4
2
0
1.0000
1.2838
1.3096
9.8124
0.6783
0.5980
1.2158
7.0468
2.8483
3.8728
5.0699
10
8
6
4
2
[ppm]

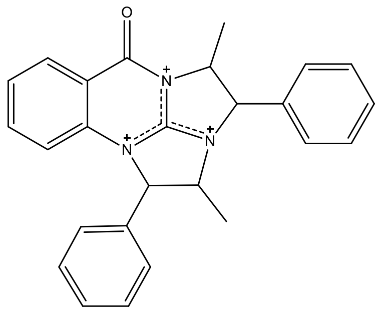

Compound 16

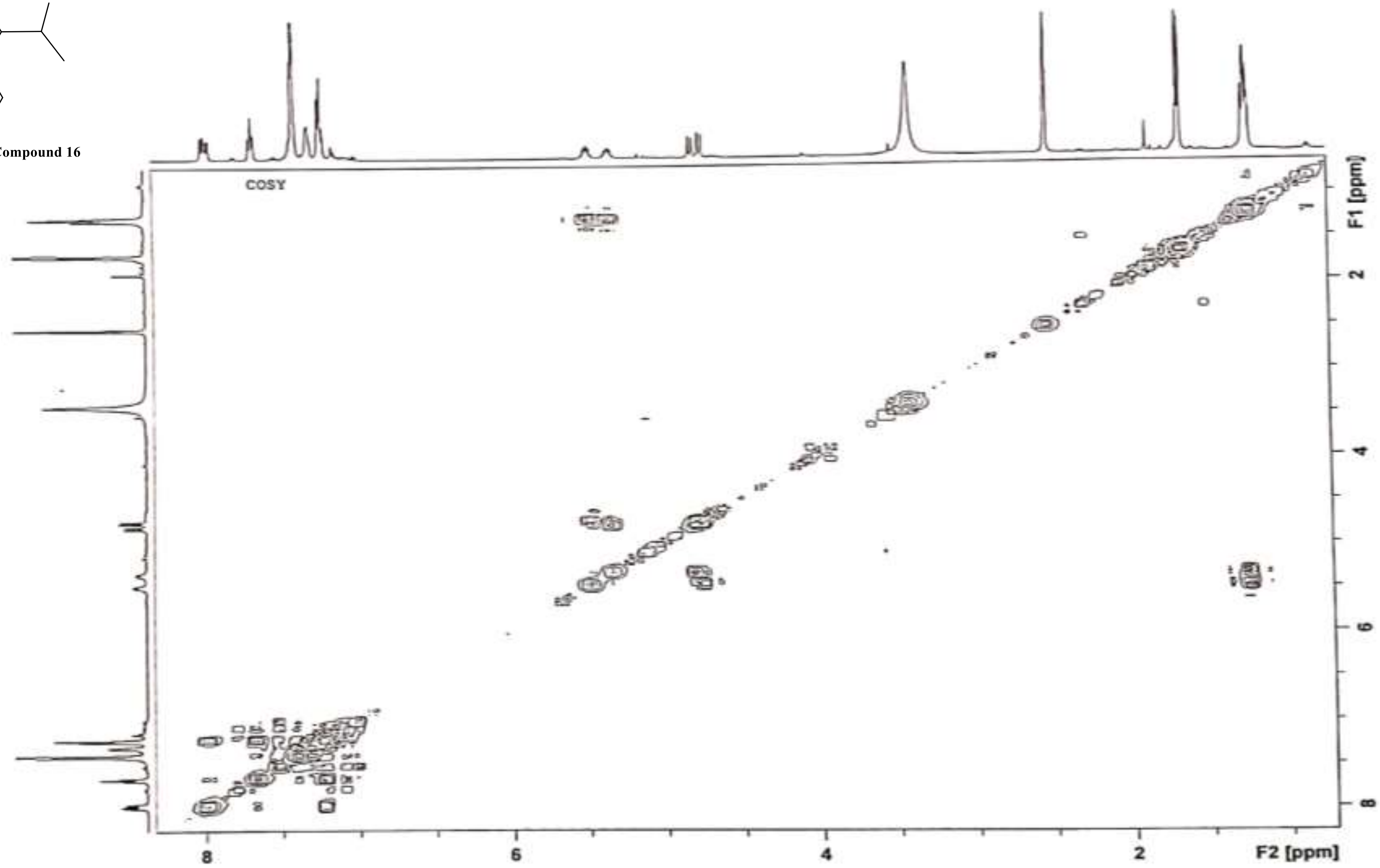

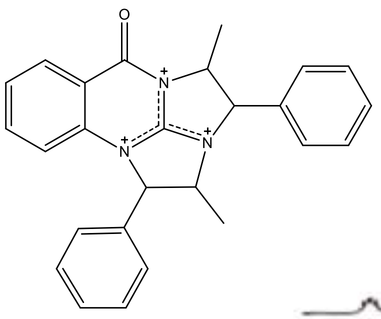

Compound 16

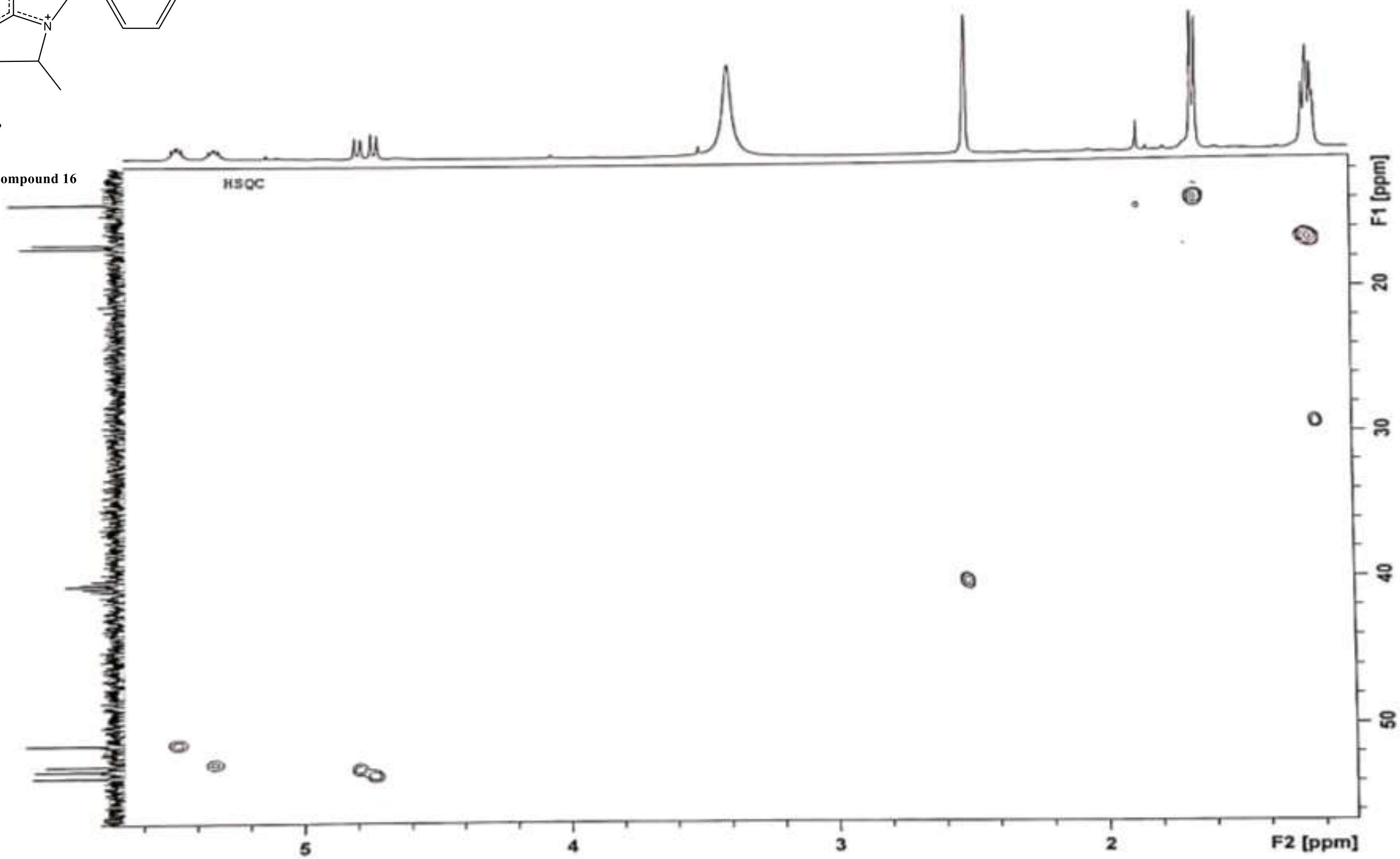

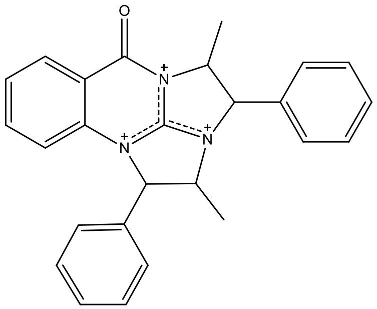

Compound 16

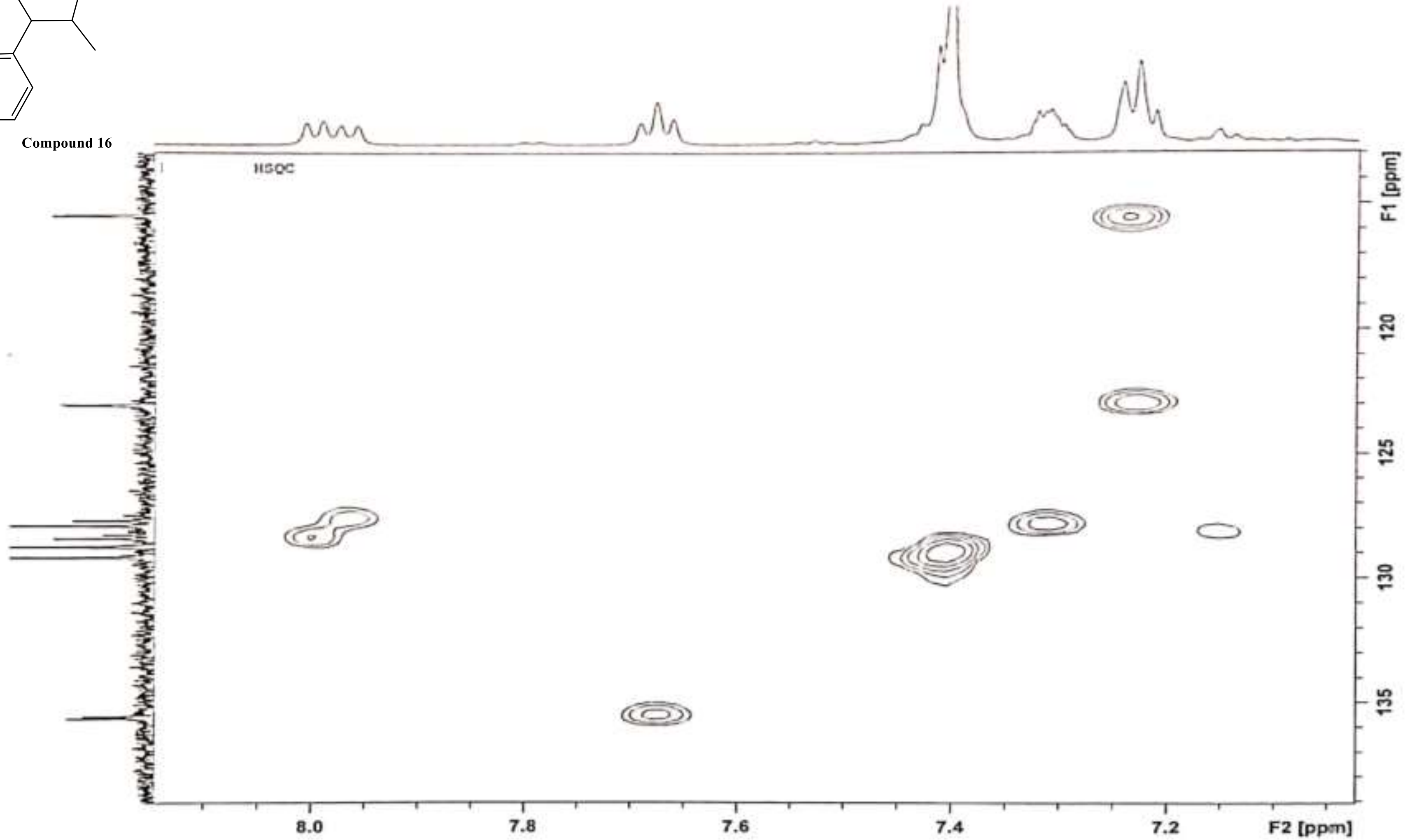

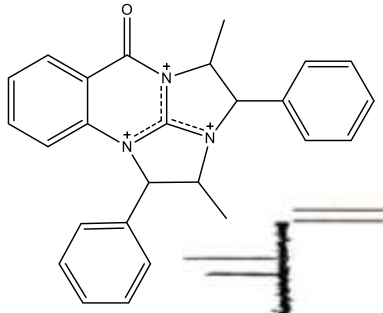

Compound 16

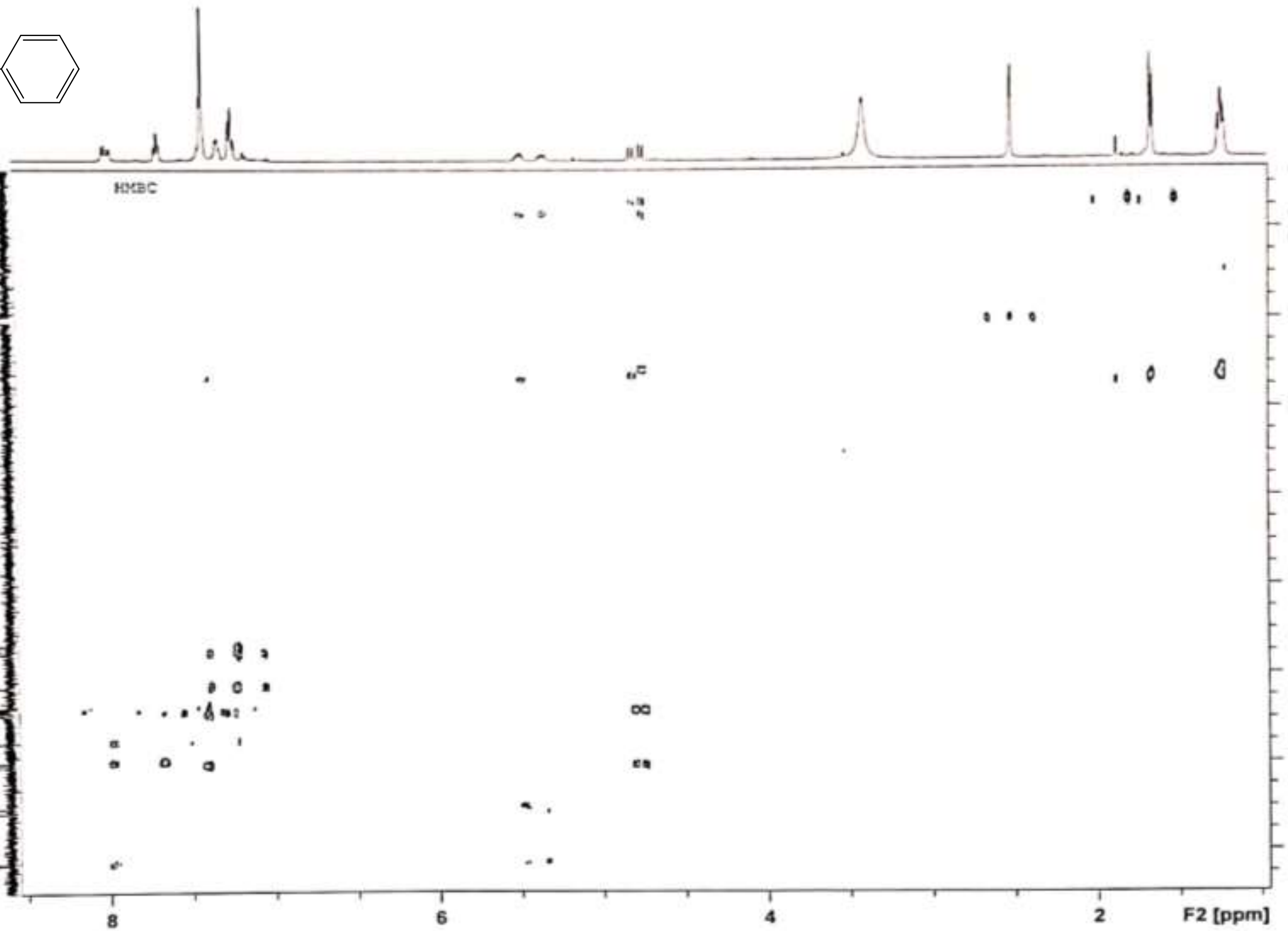

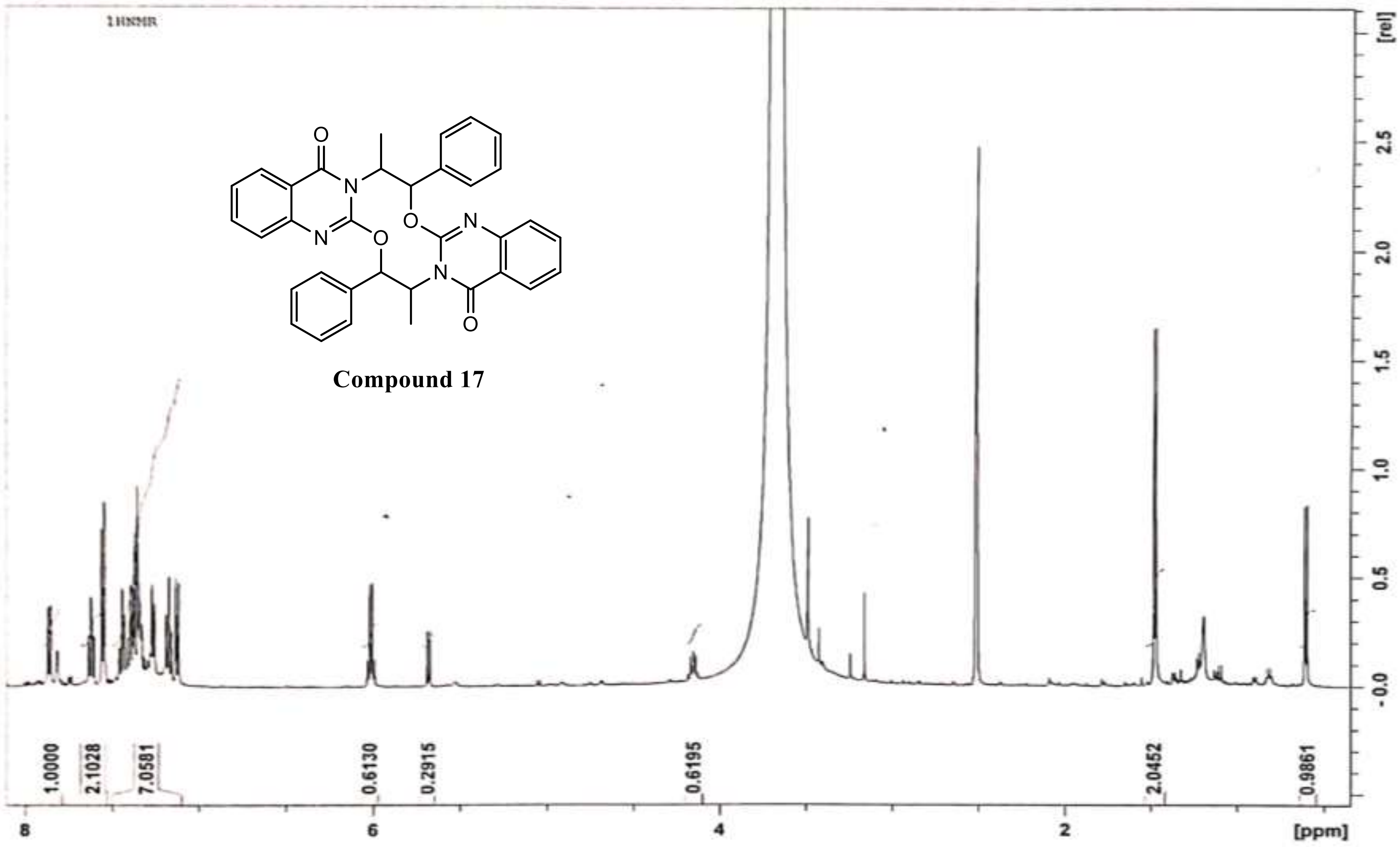
1HNMR
Compound 17
[rel]
2.5
2.0
1.5
1.0
0.5
-0.0
1.0000
2.1028
7.0581
0.6130
0.2915
0.6195
2.0452
0.9861
8
6
4
2
[ppm]

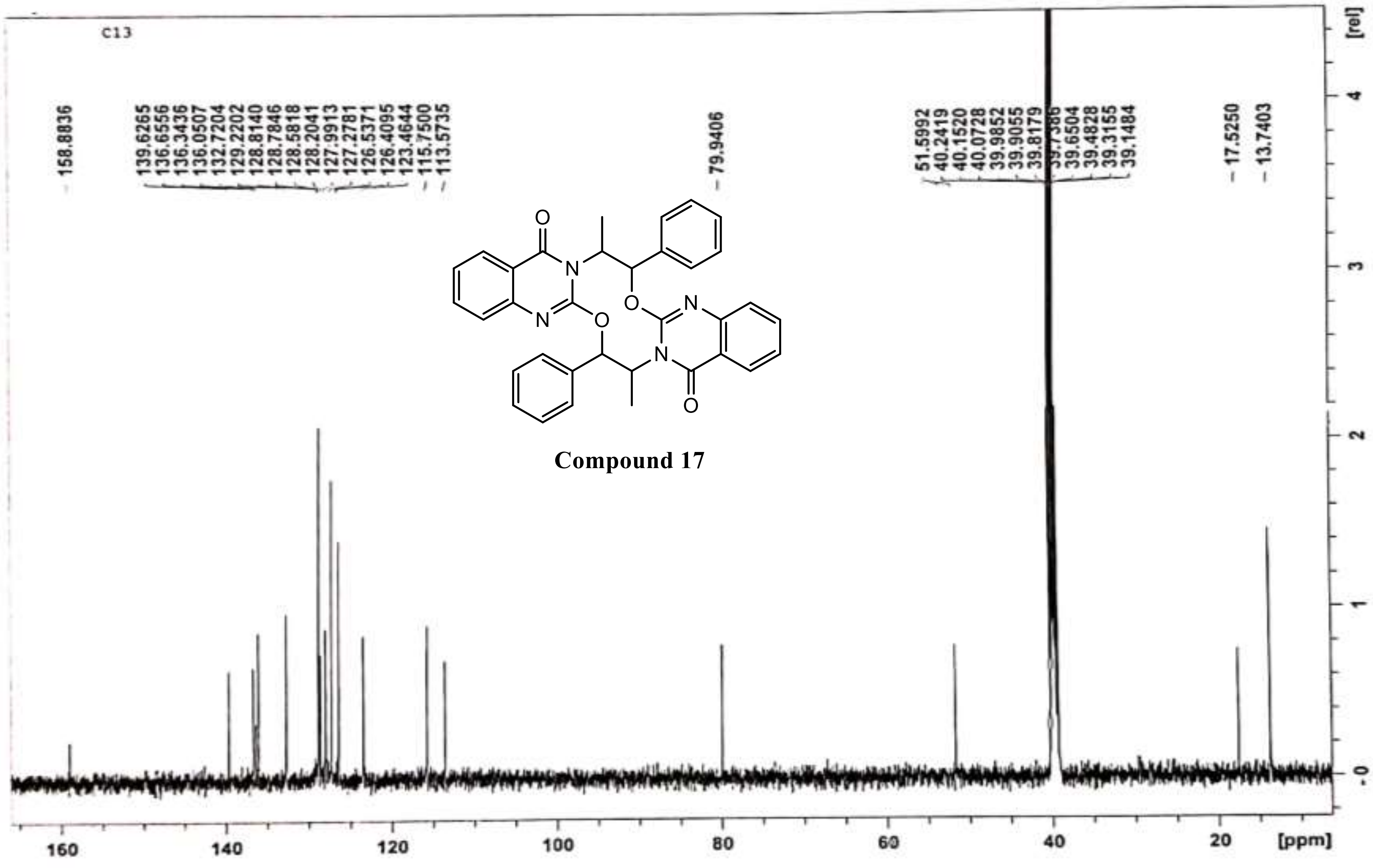
C13
158.8836
139.6265
136.6556
136.3436
136.0507
132.7204
129.2202
128.8140
128.7846
128.5818
128.2041
127.9913
127.2781
126.5371
126.4095
123.4644
115.7500
113.5735
79.9406
51.5992
40.2419
40.1520
40.0728
39.9852
39.9055
39.8179
39.6504
39.4828
39.3155
39.1484
17.5250
13.7403
[rel]
160
140
120
100
80
60
40
20
[ppm]

Compound 17

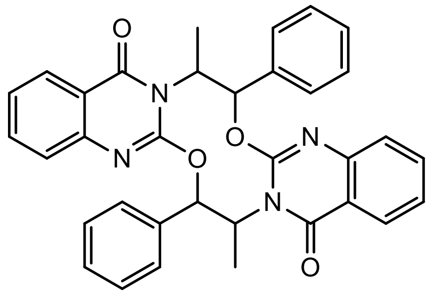

Compound 17

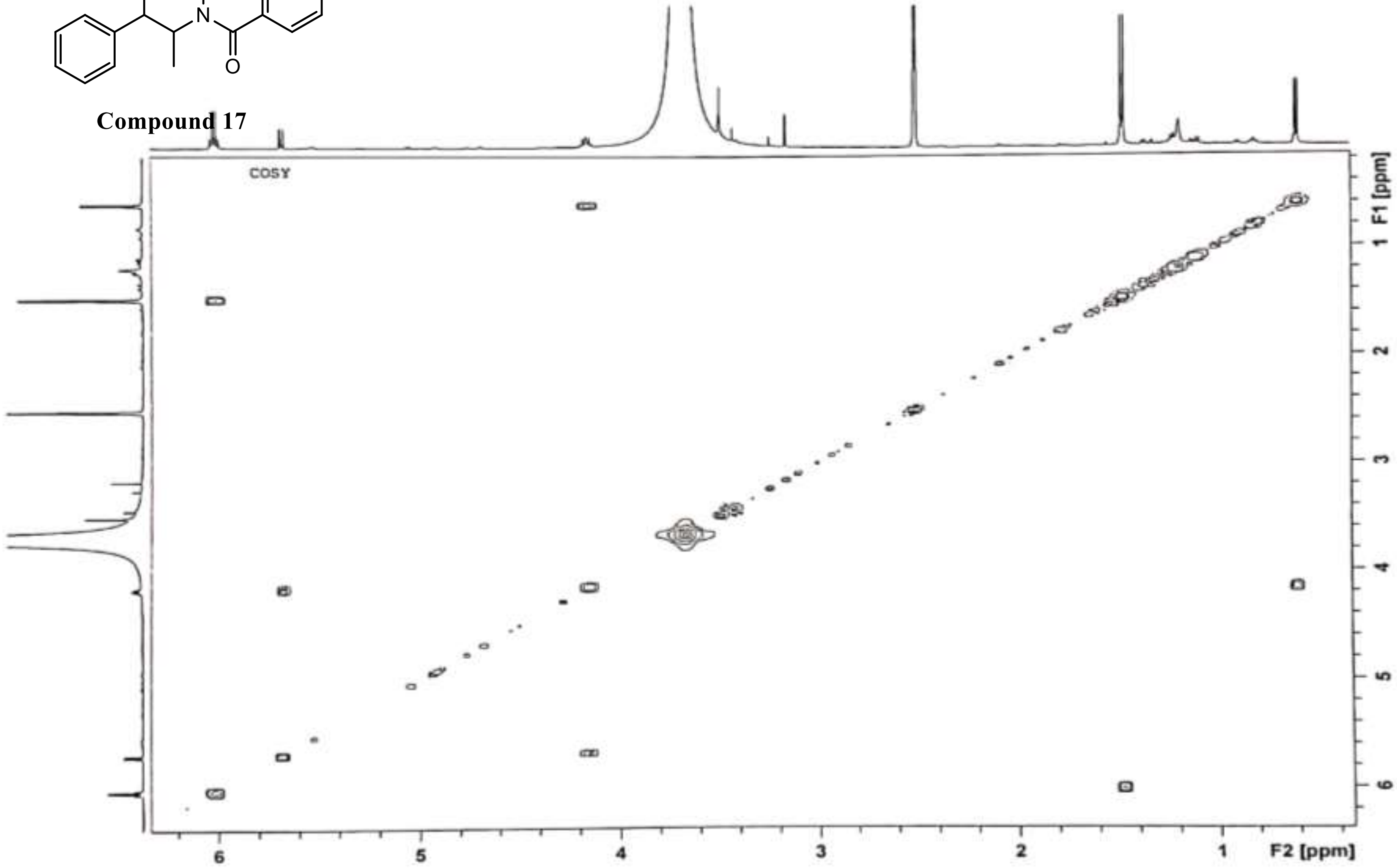

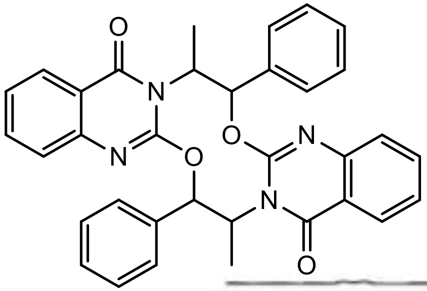

Compound 17

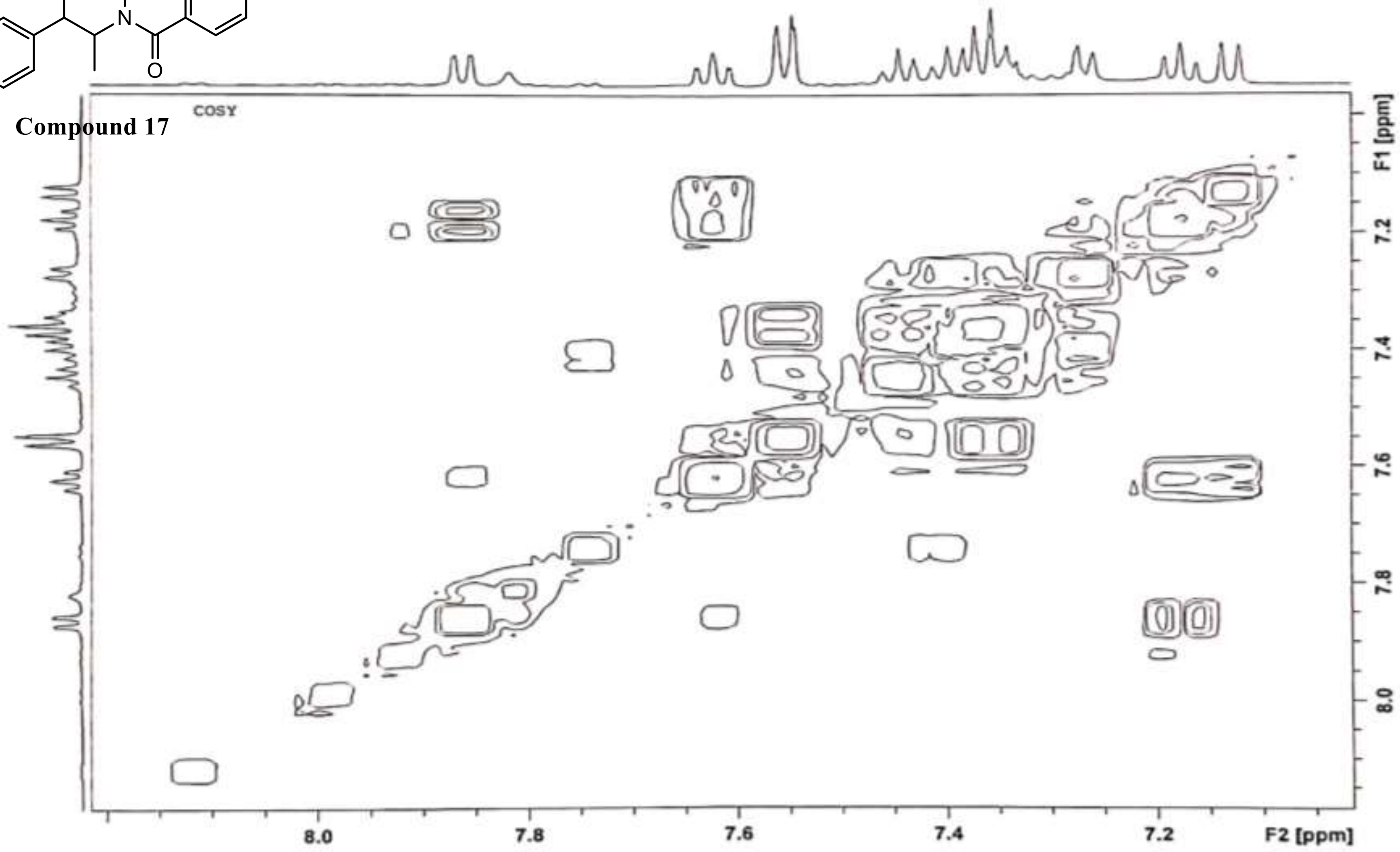

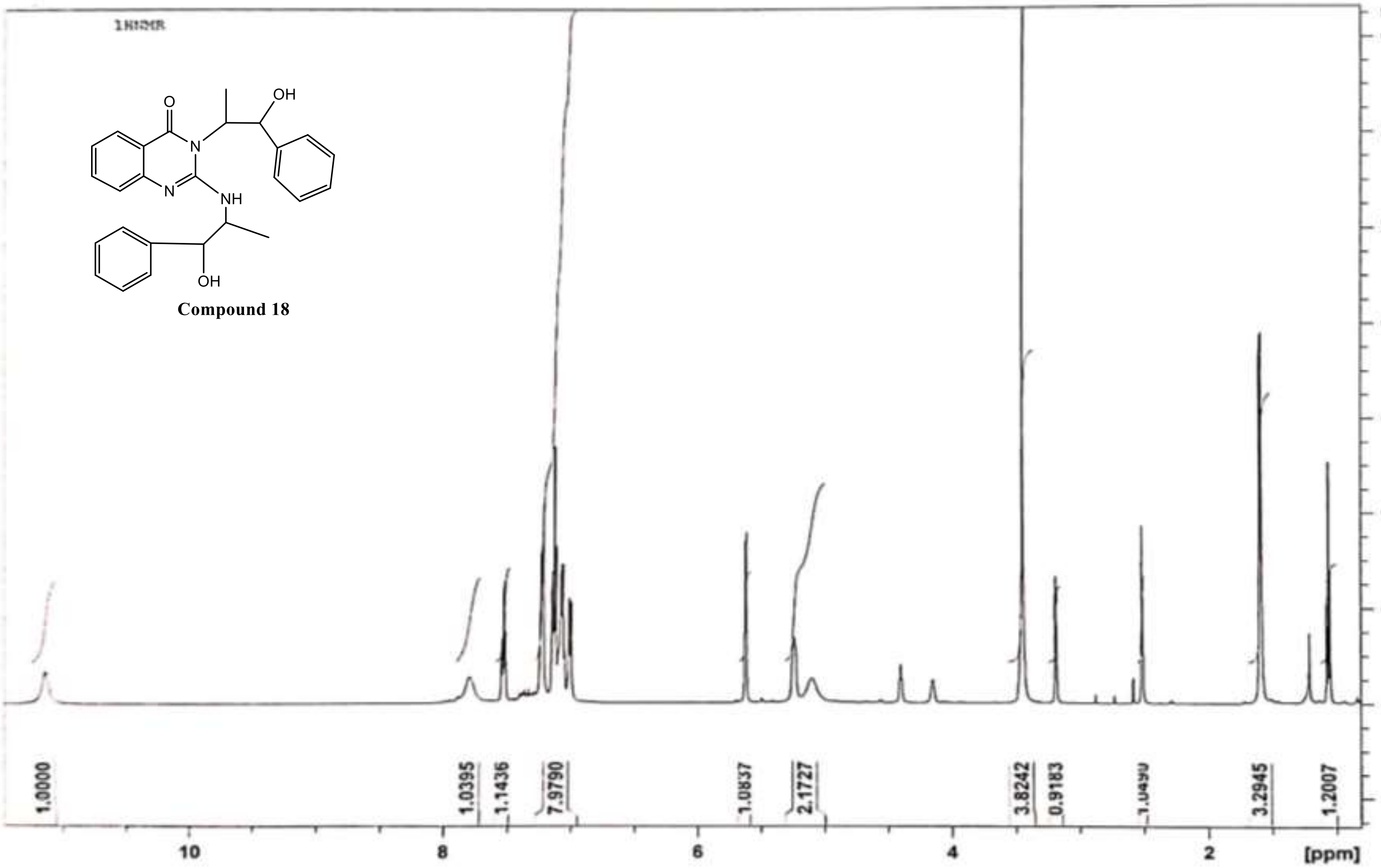
1HNMR
O
OH
N
N
NH
OH
[rel]
1.0000
1.0395
1.1436
7.9790
1.0837
2.1727
3.8242
0.9183
3.2945
1.2007
10
8
6
4
2
[ppm]

Compound 18

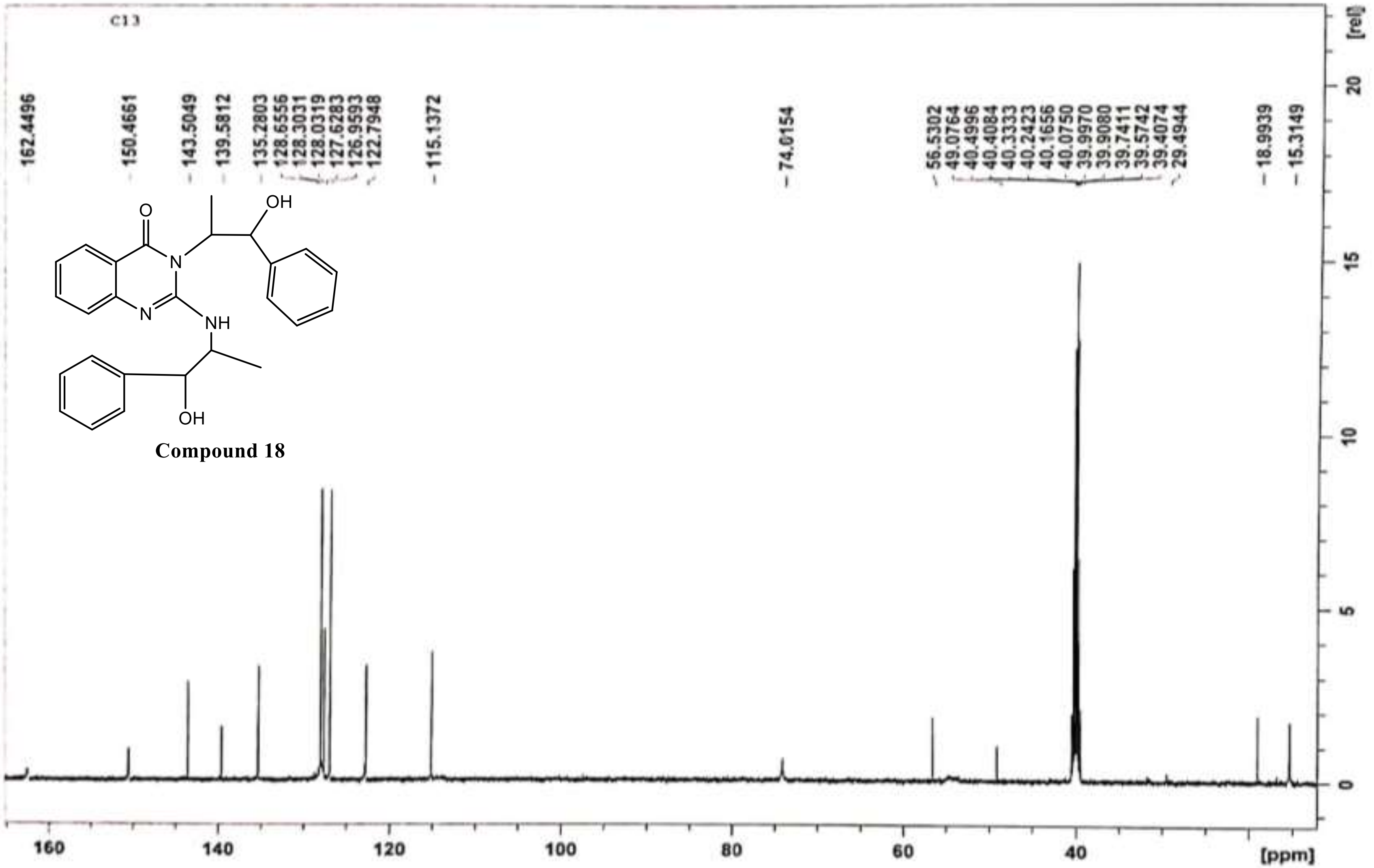
C13
162.4496
150.4661
143.5049
139.5812
135.2803
128.6556
128.3031
128.0319
127.6283
126.9593
122.7948
115.1372
74.0154
56.5302
49.0764
40.4996
40.4084
40.3333
40.2423
40.1656
40.0750
39.9970
39.9080
39.7411
39.5742
39.4074
29.4944
18.9939
15.3149
O
N
N
NH
OH
OH
Compound 18
160
140
120
100
80
60
40
[ppm]
[rel]
0
5
10
15
20

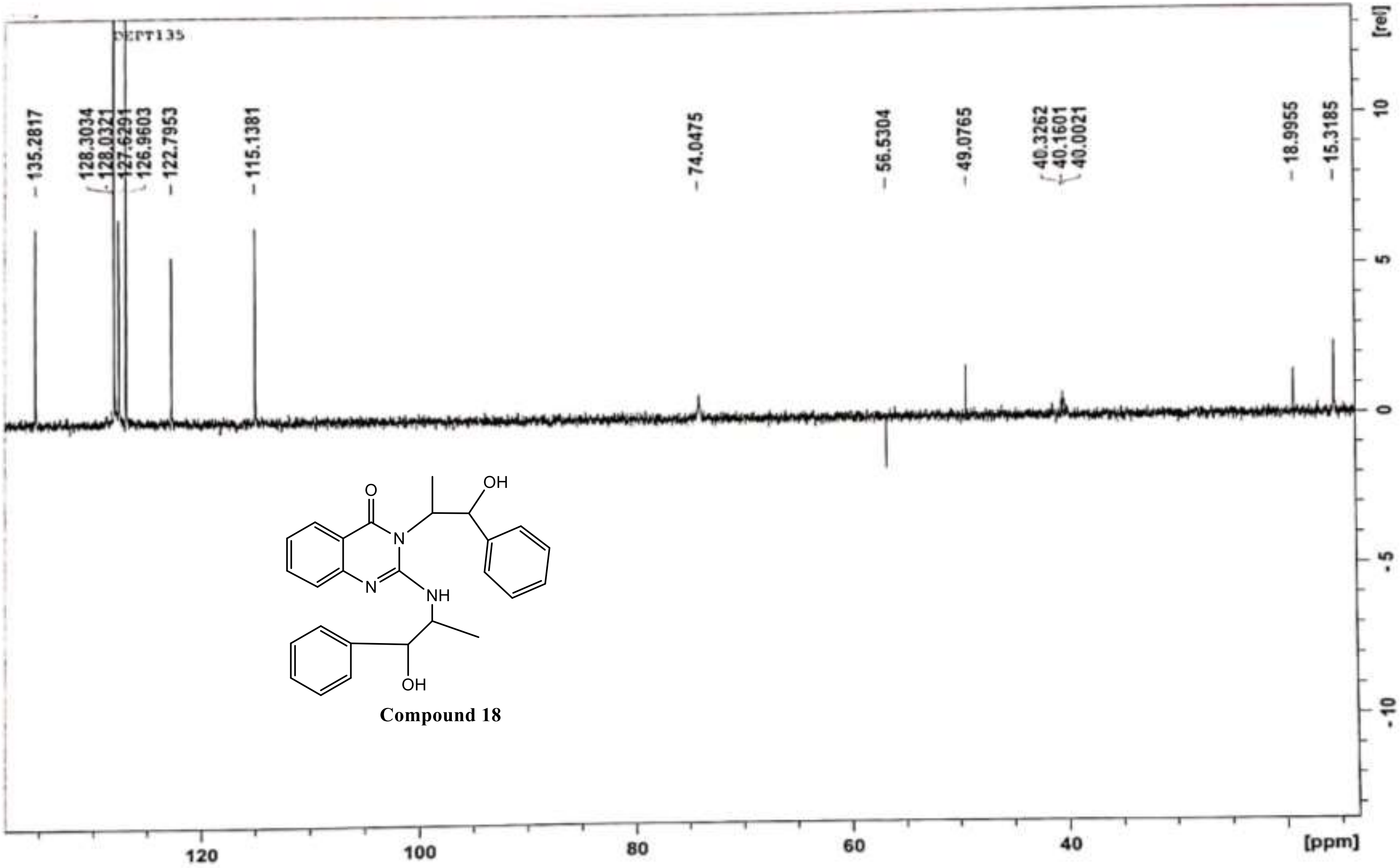
DEPT135
135.2817
128.3034
128.0321
127.6291
126.9603
122.7953
115.1381
74.0475
56.5304
49.0765
40.3262
40.1601
40.0021
18.9955
15.3185
[rel]
10
5
0
-5
-10
120
100
80
60
40
[ppm]
O
OH
N
NH
OH

Compound 18

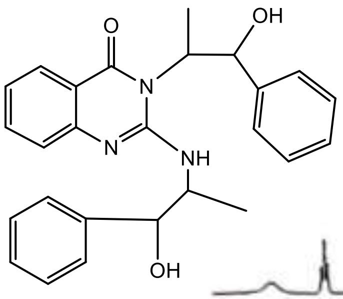

Compound 18

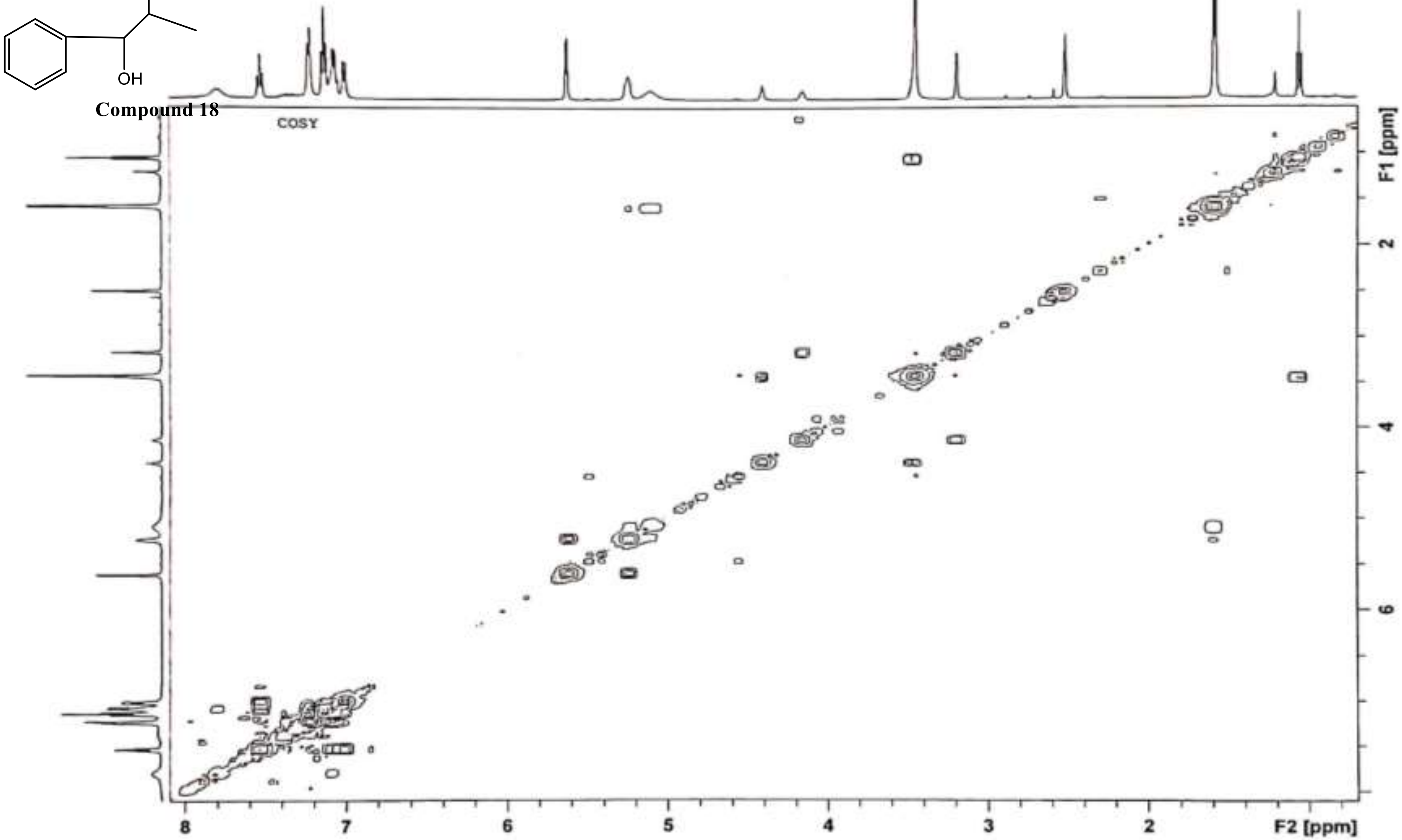

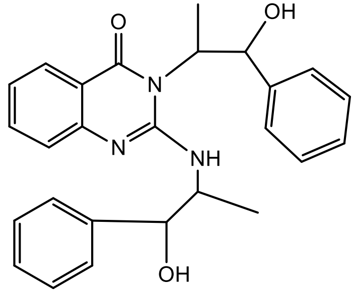

Compound 18

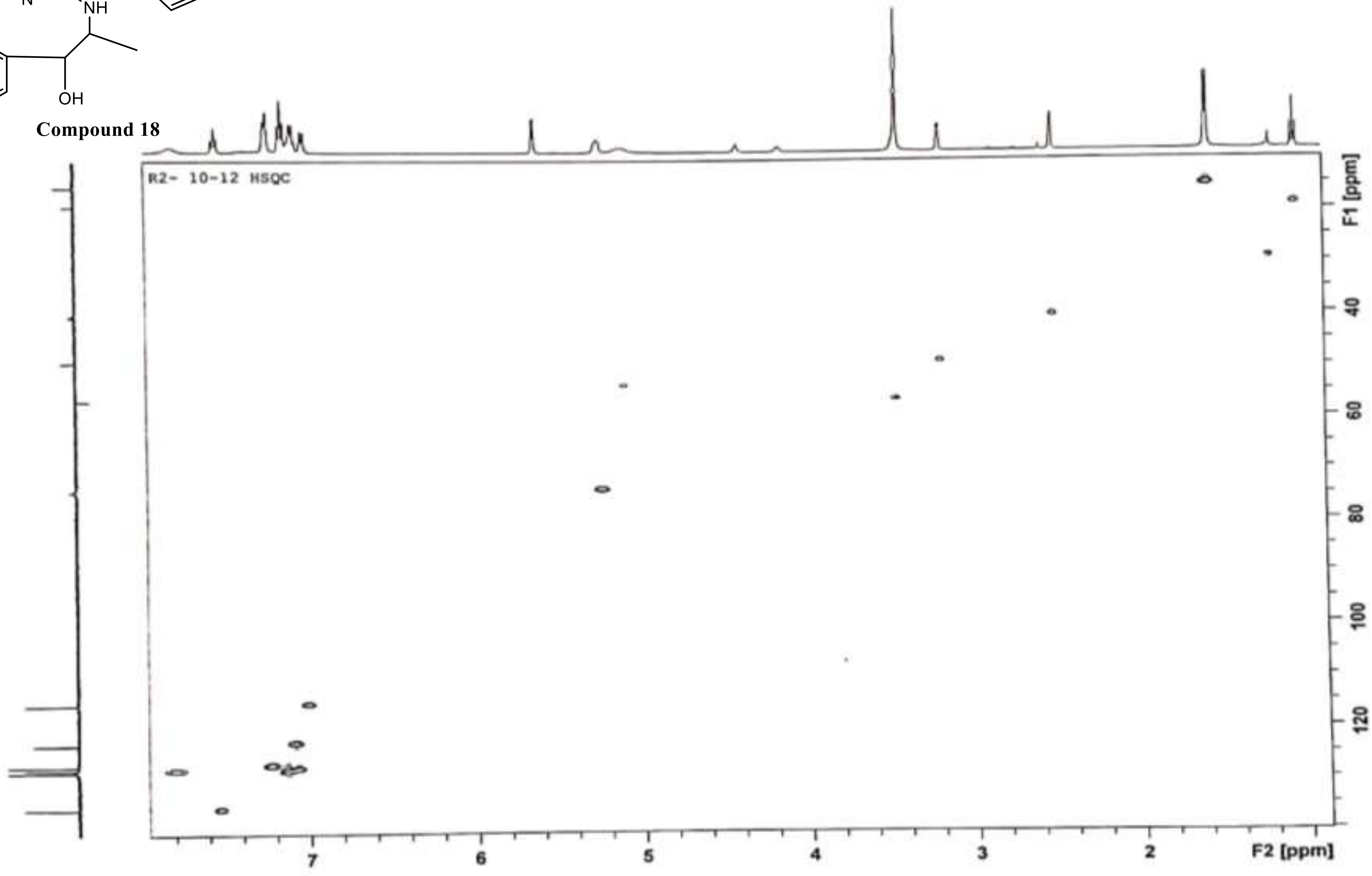

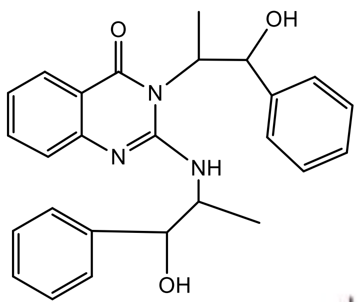

Compound 18

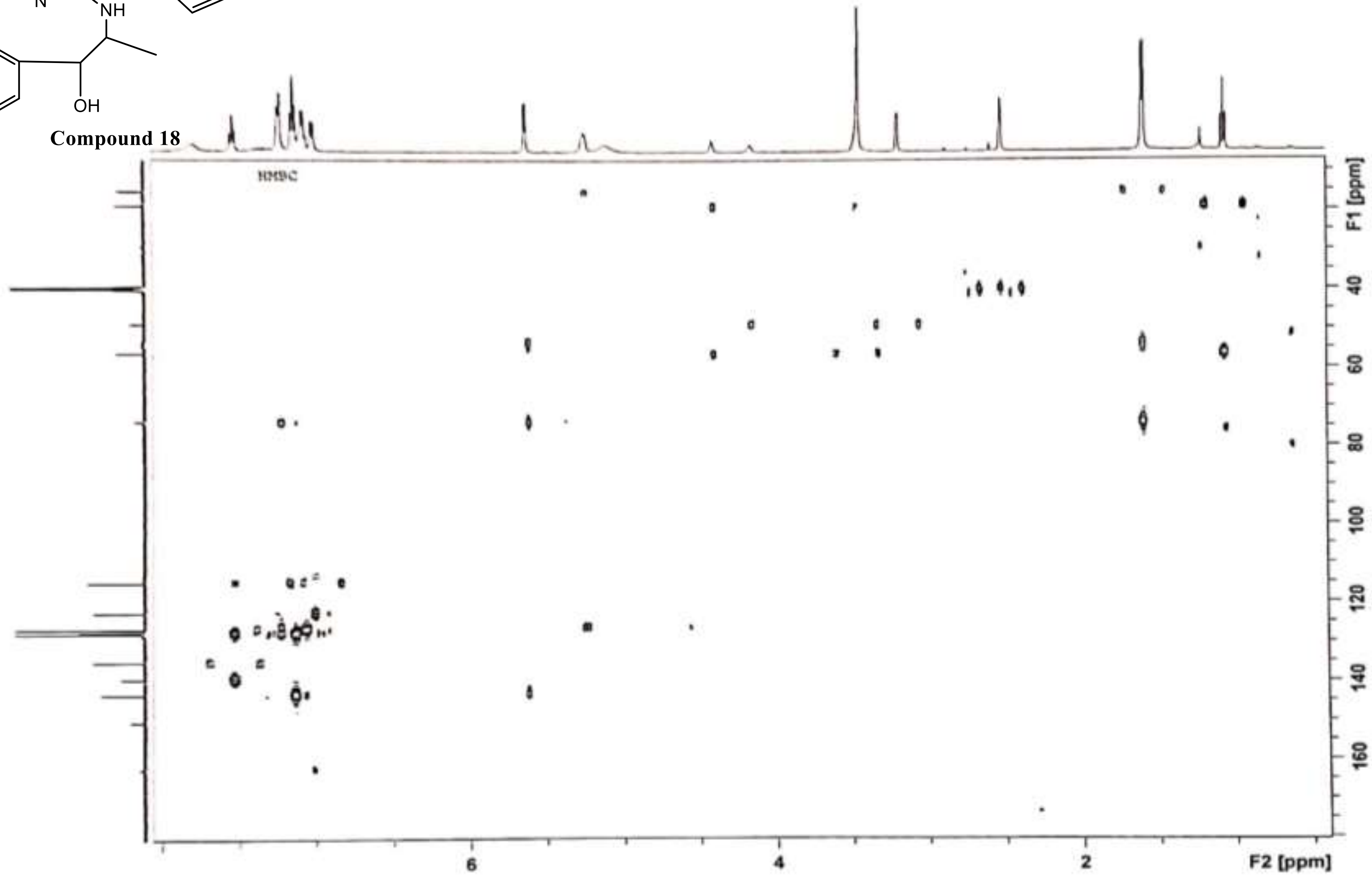

Supplement: Supplemental Material [file IENZ_A_1854243_SM5256.zip › Supplementary data 2.pdf]
