# Supplementary material for: Synthesis of some quinazolinones inspired from the natural alkaloid L-norephedrine as EGFR inhibitors and radiosensitizers
Source: J Enzyme Inhib Med Chem. 2020 Dec 28;36(1):218–38. doi: 10.1080/14756366.2020.1854243 (PMC7781899; doi:10.1080/14756366.2020.1854243)
Supplement: Supplemental Material [file IENZ_A_1854243_SM5256.zip › Supplementary data1.pdf]

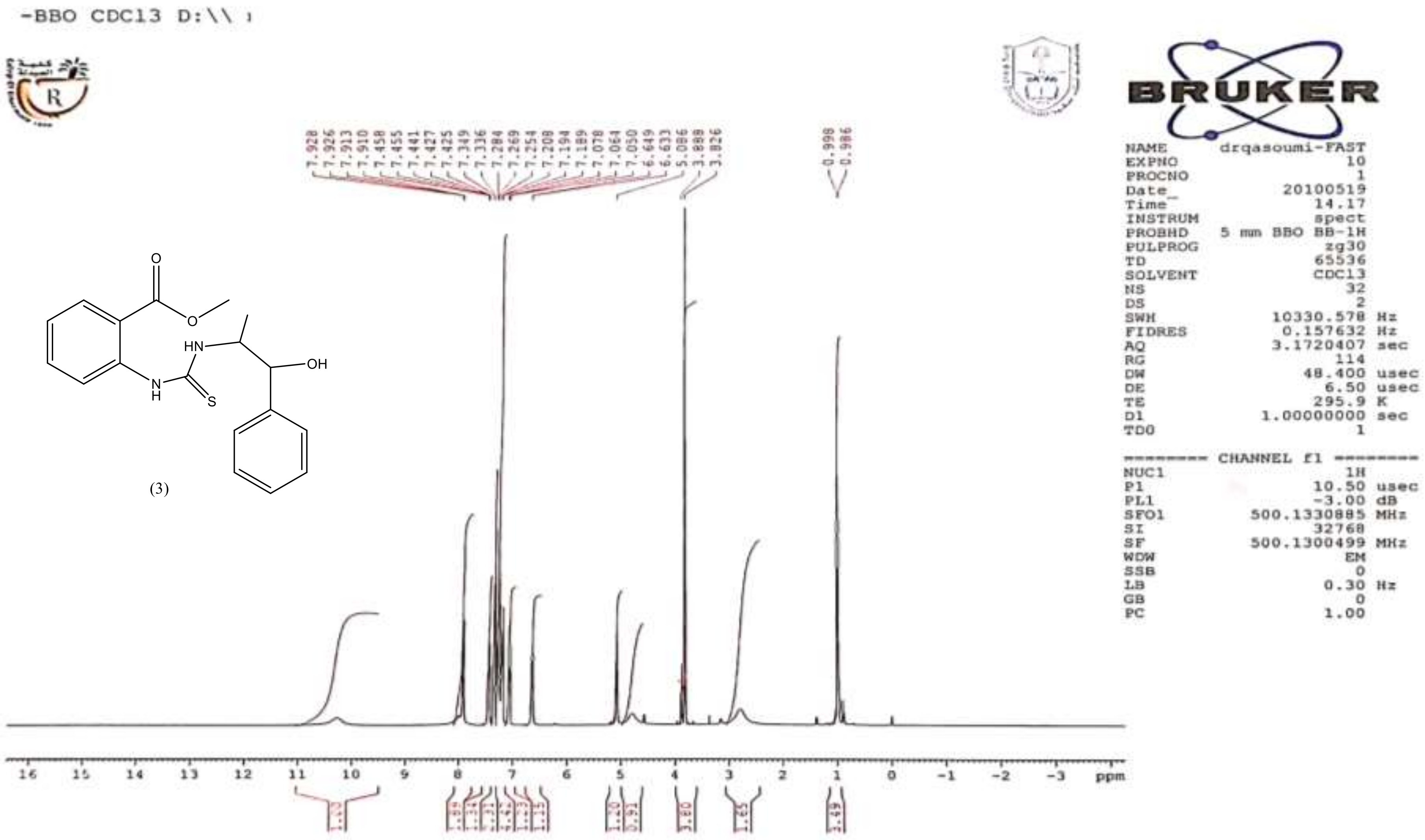
-BBO CDCl3 D:\\ :
7.928 7.926 7.913 7.910 7.458 7.455 7.441 7.427 7.425 7.349 7.336 7.284 7.269 7.254 7.208 7.194 7.189 7.078 7.064 7.050 6.649 6.633 5.086 3.889 3.826
0.998 0.986
O
O
HN
OH
N
H
S
(3)
BRUKER
NAME drqasoumi-FAST
EXPNO 10
PROCNO 1
Date_ 20100519
Time_ 14.17
INSTRUM spect
PROBHD 5 mm BBO BB-1H
PULPROG zg30
TD 65536
SOLVENT CDCl3
NS 32
DS 2
SWH 10330.578 Hz
FIDRES 0.157632 Hz
AQ 3.1720407 sec
RG 114
DW 48.400 usec
DE 6.50 usec
TE 295.9 K
D1 1.00000000 sec
TD0 1
======== CHANNEL f1 ========
NUC1 1H
P1 10.50 usec
PL1 -3.00 dB
SFO1 500.1330885 MHz
SI 32768
SF 500.1300499 MHz
WDW EM
SSB 0
LB 0.30 Hz
GB 0
PC 1.00
16 15 14 13 12 11 10 9 8 7 6 5 4 3 2 1 0 -1 -2 -3 ppm

-BBO CDCl3 D:\\ :

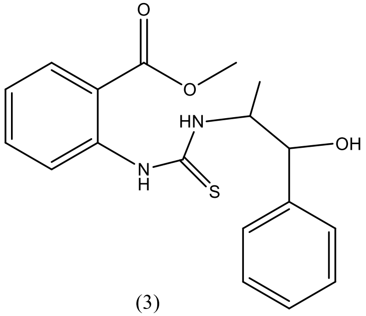

(3)

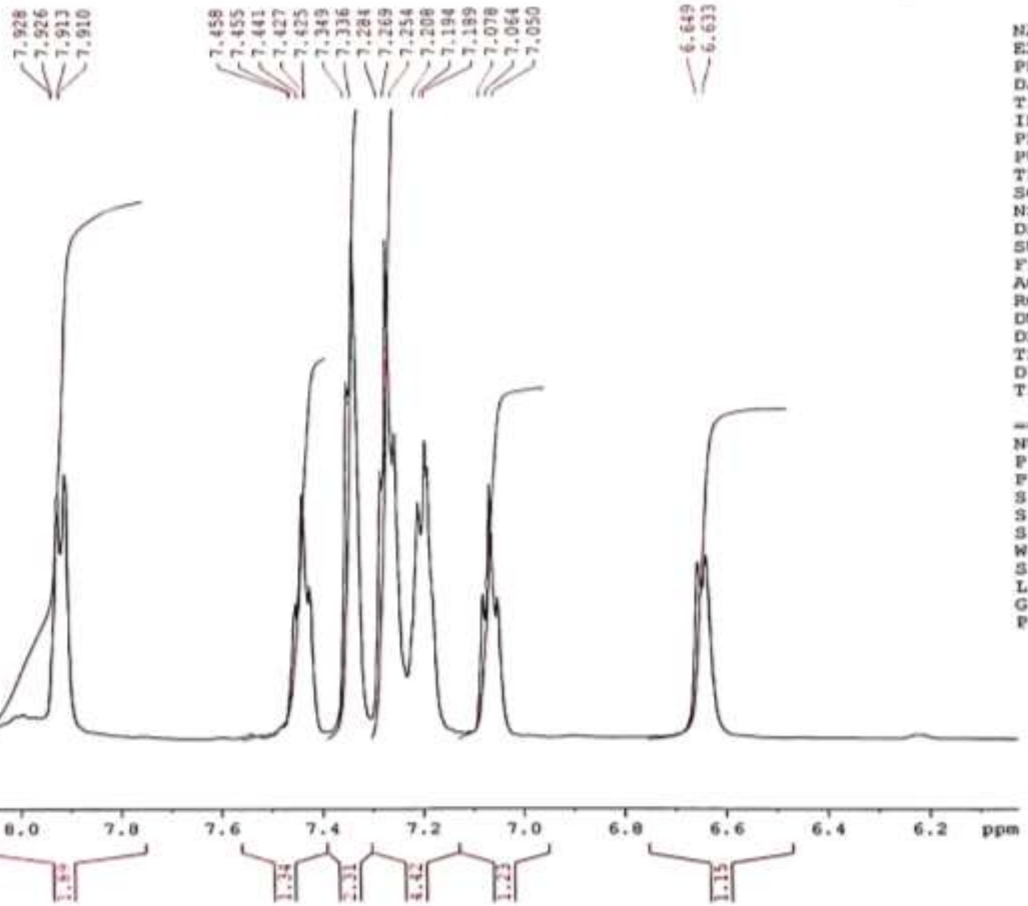

BRUKER

NAME	drqasoumi-FAST
EXPNO	10
PROCNO	1
Date_	20100519
Time	14.17
INSTRUM	spect
PROBHD	5 mm BBO BB-1H
PULPROG	zg30
TD	65536
SOLVENT	CDCl3
NS	32
DS	2
SWH	10330.578 Hz
FIDRES	0.157632 Hz
AQ	3.1720407 sec
RG	114
DW	48.400 usec
DE	6.50 usec
TE	295.9 K
D1	1.00000000 sec
TD0	1

======== CHANNEL f1 ========

NUC1	1H
P1	10.50 usec
PL1	-3.00 dB
SFO1	500.1330885 MHz
SI	32768
SF	500.1300499 MHz
WDW	EM
SSB	0
LB	0.30 Hz
GB	0
PC	1.00

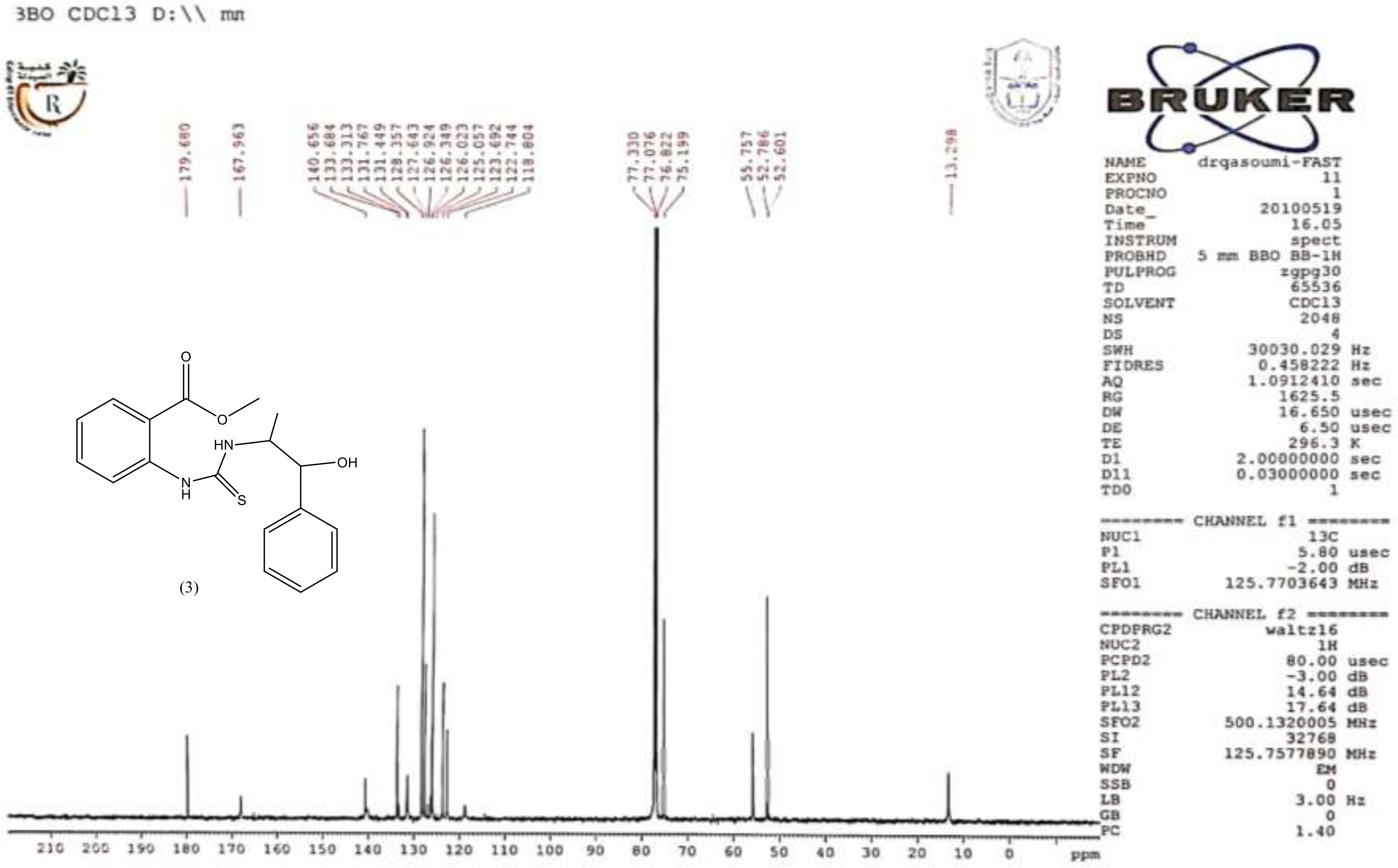
3BO CDCl3 D:\\ mn
179.680
167.963
140.656 133.684 133.313 131.767 131.449 128.357 127.643 126.924 126.349 126.023 125.057 123.692 122.744 118.804
77.330 77.076 76.822 75.199
55.757 52.786 52.601
13.298
BRUKER
NAME drqasoumi-FAST
EXPNO 11
PROCNO 1
Date_ 20100519
Time 16.05
INSTRUM spect
PROBHD 5 mm BBO BB-1H
PULPROG zgpg30
TD 65536
SOLVENT CDCl3
NS 2048
DS 4
SWH 30030.029 Hz
FIDRES 0.458222 Hz
AQ 1.0912410 sec
RG 1625.5
DW 16.650 usec
DE 6.50 usec
TE 296.3 K
D1 2.00000000 sec
D11 0.03000000 sec
TD0 1
======== CHANNEL f1 ========
NUC1 13C
P1 5.80 usec
PL1 -2.00 dB
SFO1 125.7703643 MHz
======== CHANNEL f2 ========
CPDPRG2 waltz16
NUC2 1H
PCPD2 80.00 usec
PL2 -3.00 dB
PL12 14.64 dB
PL13 17.64 dB
SFO2 500.1320005 MHz
SI 32768
SF 125.7577890 MHz
WDW EM
SSB 0
LB 3.00 Hz
GB 0
PC 1.40
O
O
HN
OH
N
H
S
(3)
210 200 190 180 170 160 150 140 130 120 110 100 90 80 70 60 50 40 30 20 10 0 ppm

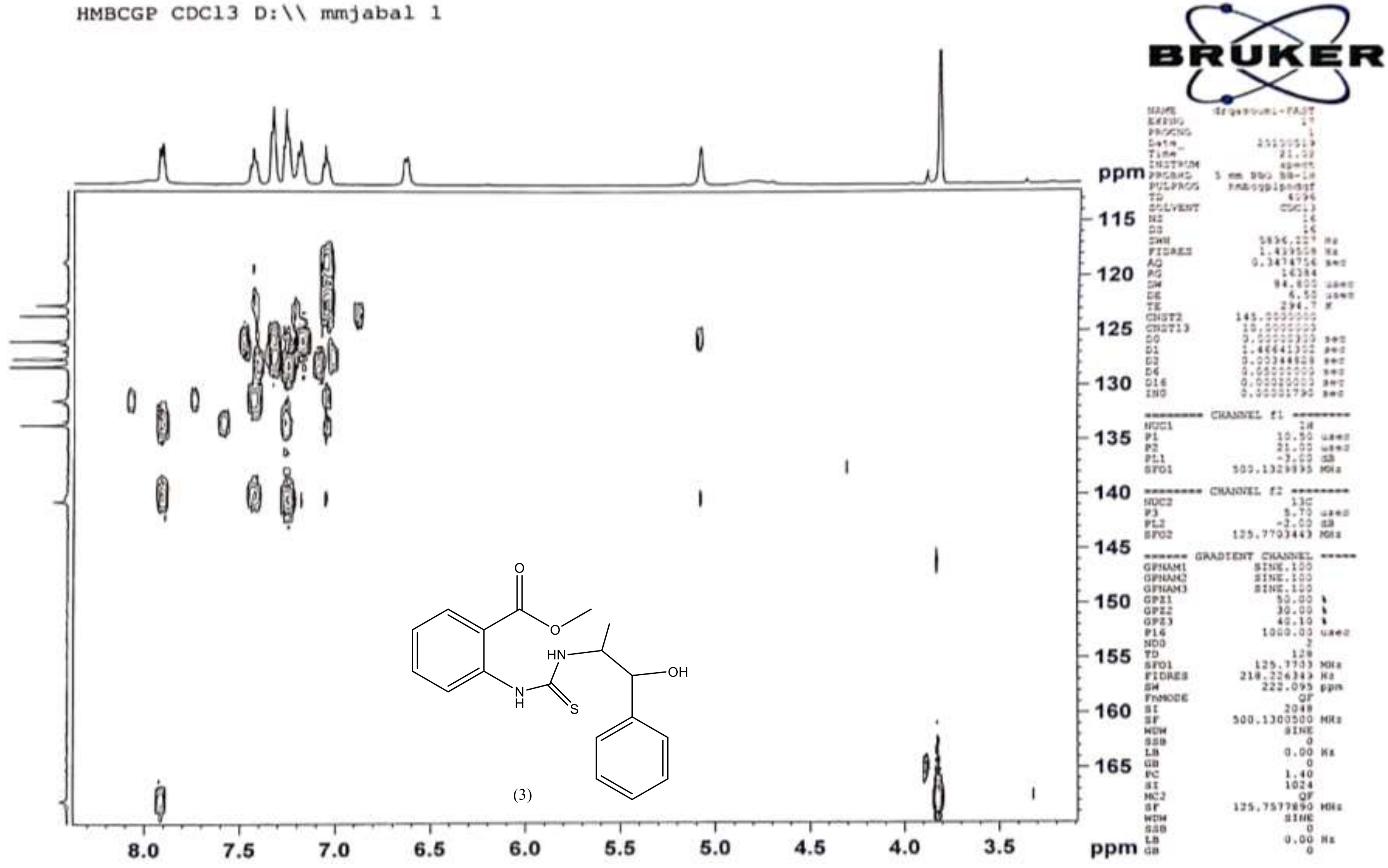
HMBCGP CDCl3 D:\\ mmjabal 1
BRUKER
ppm
115
120
125
130
135
140
145
150
155
160
165
8.0
7.5
7.0
6.5
6.0
5.5
5.0
4.5
4.0
3.5
ppm
O
O
HN
OH
N
H
S
(3)

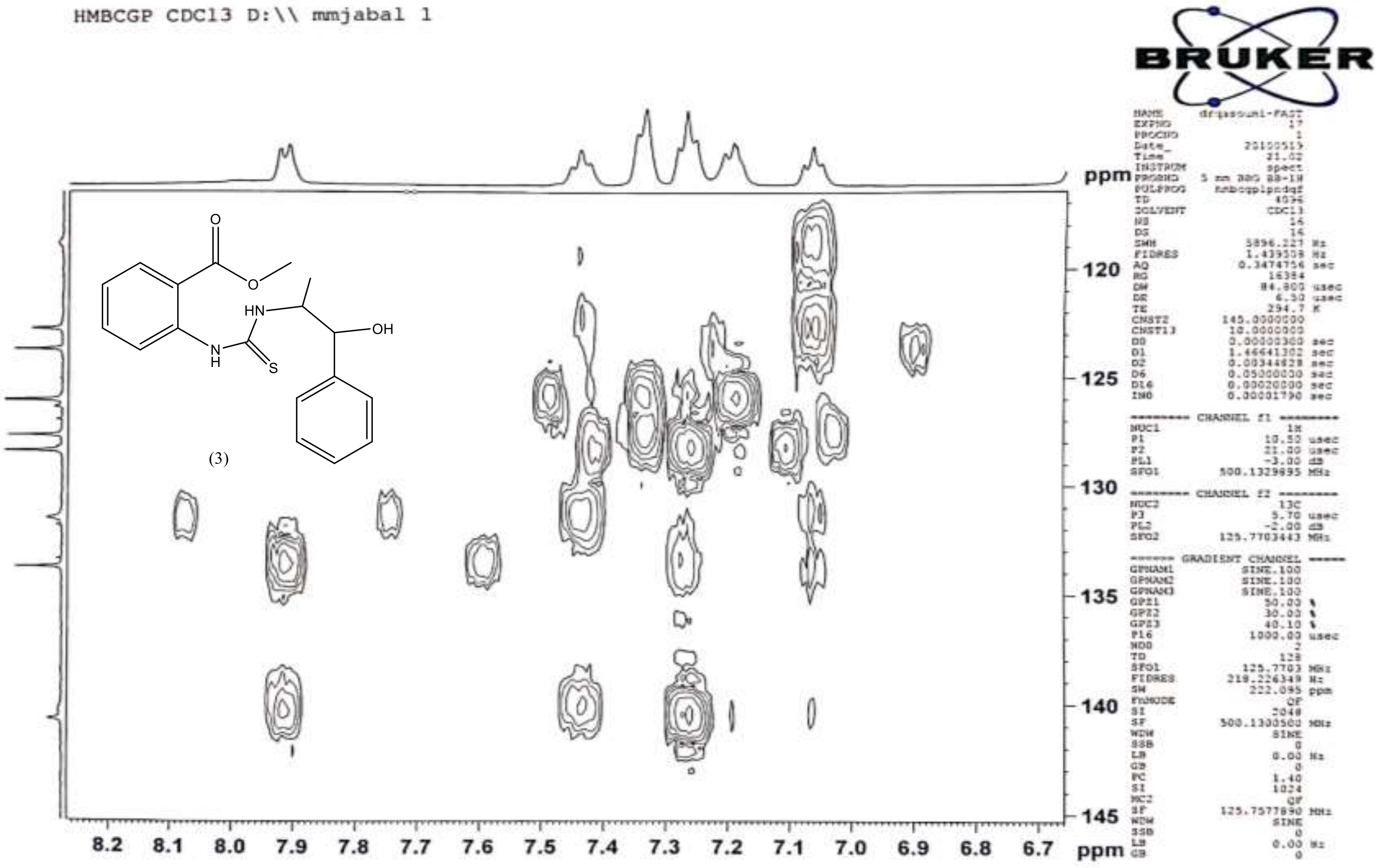
HMBCGP CDCl3 D:\\ mmjabal 1
BRUKER
O
O
HN
OH
N
H
S
(3)
ppm
120
125
130
135
140
145
8.2 8.1 8.0 7.9 7.8 7.7 7.6 7.5 7.4 7.3 7.2 7.1 7.0 6.9 6.8 6.7 ppm

[Mass Spectrum]
Data : nrc656
Sample: M.G-1
Note : -
Inlet : Direct   Ion Mode : EI+
Spectrum Type : Normal Ion [MF-Linear]
RT : 8.93 min   Scan# : 135
BP : m/z 179.0000   Int. : 18.94
Output m/z range : 40.0000 to 400.0000   Cut Level : 0.30 %

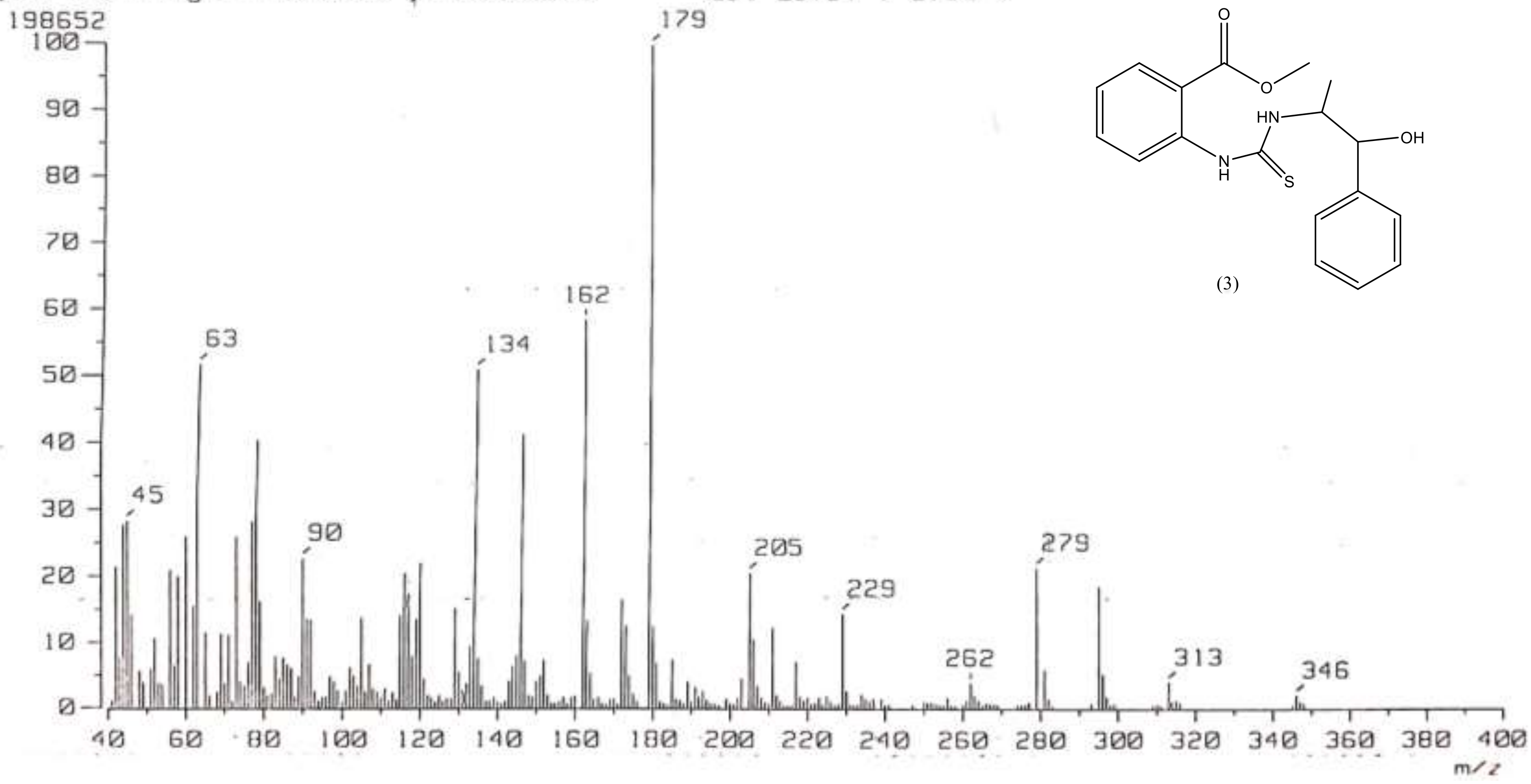

(3)

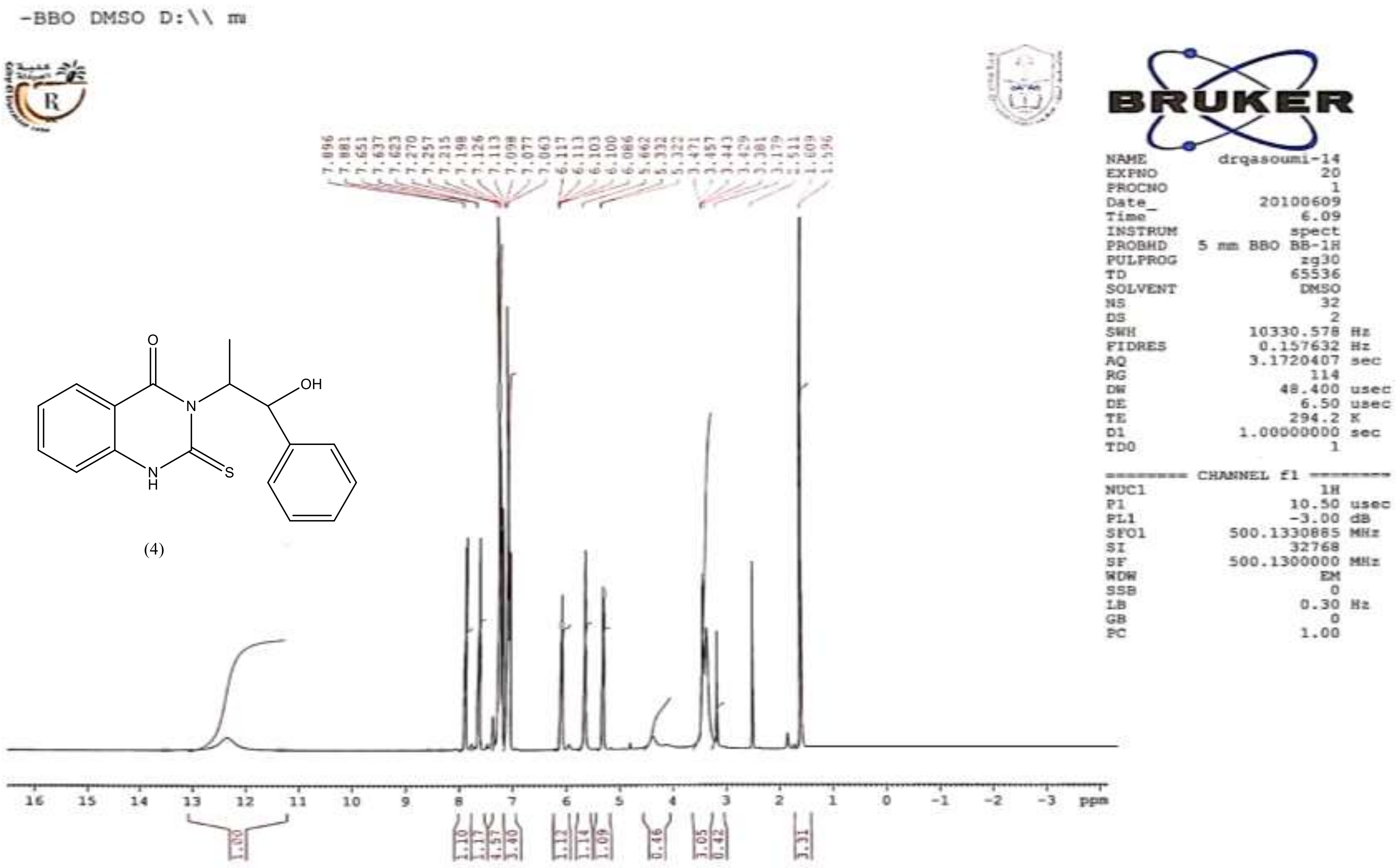
-BBO DMSO D:\\ m
BRUKER
NAME drqasoumi-14
EXPNO 20
PROCNO 1
Date_ 20100609
Time 6.09
INSTRUM spect
PROBHD 5 mm BBO BB-1H
PULPROG zg30
TD 65536
SOLVENT DMSO
NS 32
DS 2
SWH 10330.578 Hz
FIDRES 0.157632 Hz
AQ 3.1720407 sec
RG 114
DW 48.400 usec
DE 6.50 usec
TE 294.2 K
D1 1.00000000 sec
TD0 1
======== CHANNEL f1 ========
NUC1 1H
P1 10.50 usec
PL1 -3.00 dB
SFO1 500.1330885 MHz
SI 32768
SF 500.1300000 MHz
WDW EM
SSB 0
LB 0.30 Hz
GB 0
PC 1.00
ppm
O
N
OH
NH
S

(4)

-BBO DMSO D:\\ m

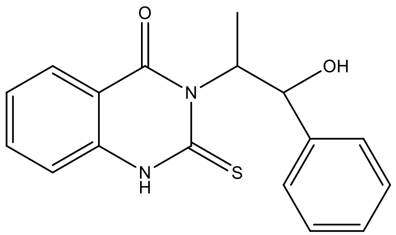

(4)

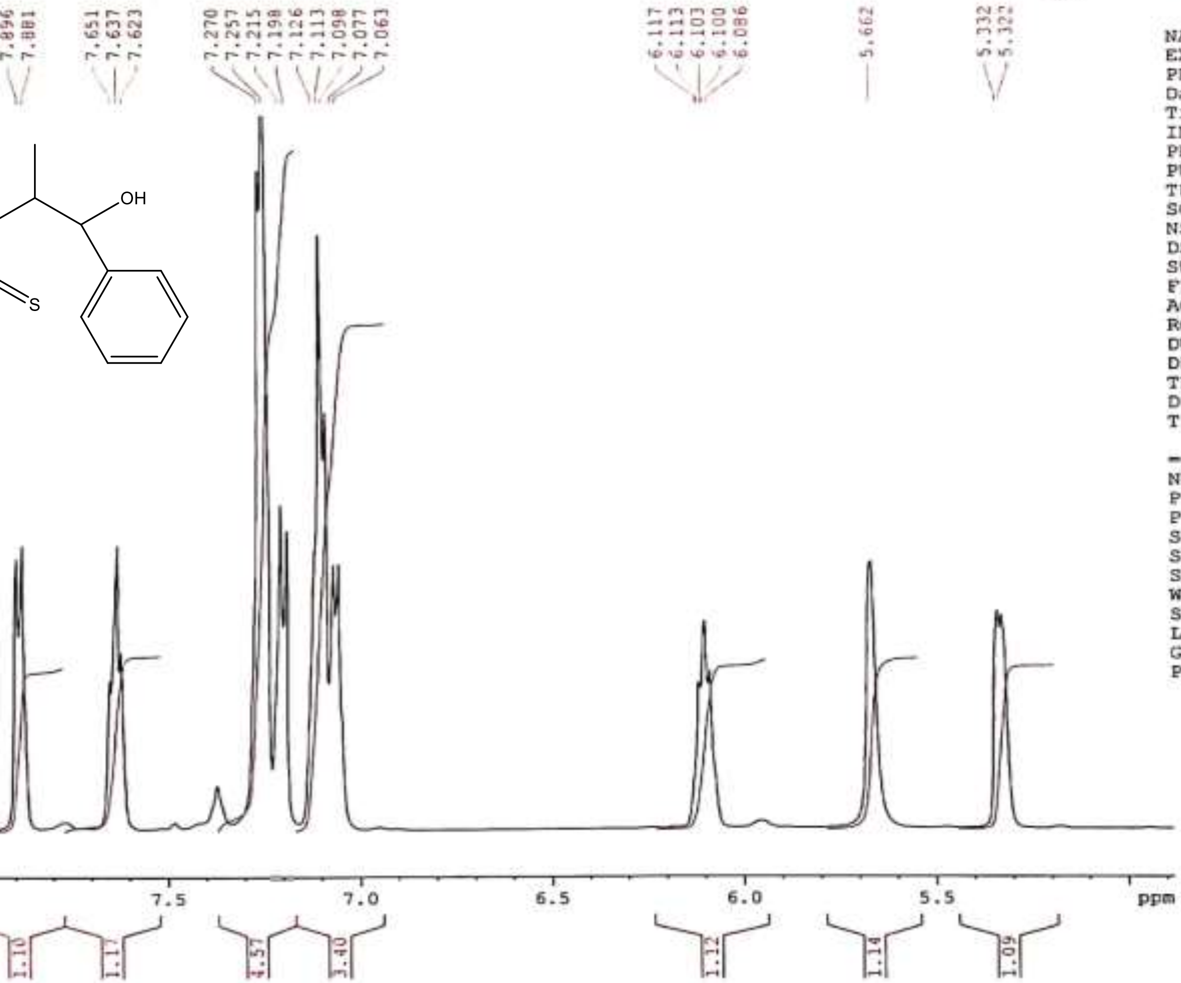

BRUKER

NAME	drqasoumi-14
EXPNO	20
PROCNO	1
Date_	20100609
Time	6.09
INSTRUM	spect
PROBHD	5 mm BBO BB-1H
PULPROG	zg30
TD	65536
SOLVENT	DMSO
NS	32
DS	2
SWH	10330.578 Hz
FIDRES	0.157632 Hz
AQ	3.1720407 sec
RG	114
DW	48.400 usec
DE	6.50 usec
TE	294.2 K
D1	1.00000000 sec
TD0	1

======== CHANNEL f1 ========

NUC1	1H
P1	10.50 usec
PL1	-3.00 dB
SFO1	500.1330885 MHz
SI	32768
SF	500.1300000 MHz
WDW	EM
SSB	0
LB	0.30 Hz
GB	0
PC	1.00

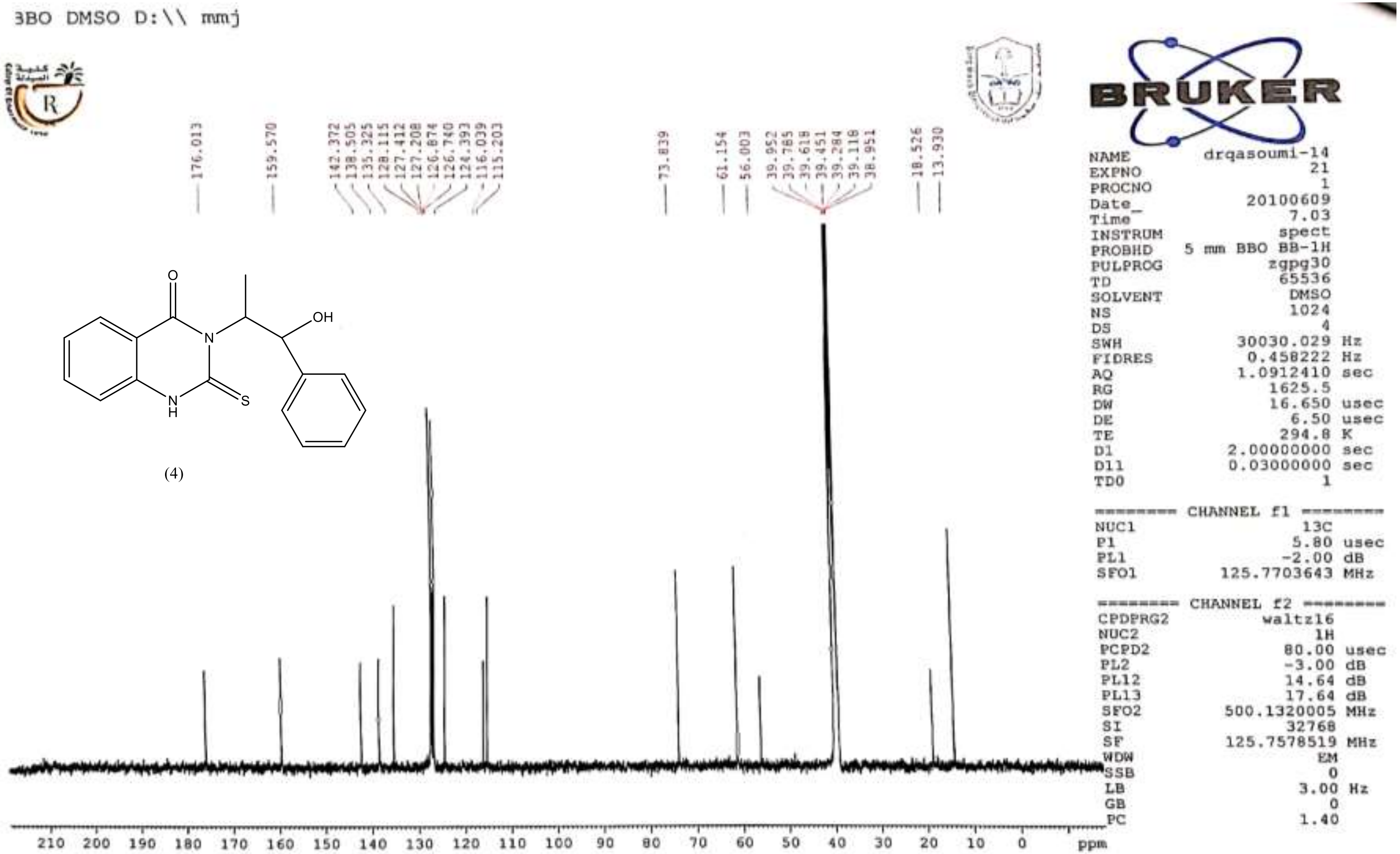
3BO DMSO D:\\ mmj
176.013
159.570
142.372
138.505
135.325
128.115
127.412
127.208
126.874
126.740
124.393
116.039
115.203
73.839
61.154
56.003
39.952
39.785
39.618
39.451
39.284
39.118
38.951
18.526
13.930
BRUKER
NAME drqasoumi-14
EXPNO 21
PROCNO 1
Date_ 20100609
Time 7.03
INSTRUM spect
PROBHD 5 mm BBO BB-1H
PULPROG zgpg30
TD 65536
SOLVENT DMSO
NS 1024
DS 4
SWH 30030.029 Hz
FIDRES 0.458222 Hz
AQ 1.0912410 sec
RG 1625.5
DW 16.650 usec
DE 6.50 usec
TE 294.8 K
D1 2.00000000 sec
D11 0.03000000 sec
TD0 1
======== CHANNEL f1 ========
NUC1 13C
P1 5.80 usec
PL1 -2.00 dB
SFO1 125.7703643 MHz
======== CHANNEL f2 ========
CPDPRG2 waltz16
NUC2 1H
PCPD2 80.00 usec
PL2 -3.00 dB
PL12 14.64 dB
PL13 17.64 dB
SFO2 500.1320005 MHz
SI 32768
SF 125.7578519 MHz
WDW EM
SSB 0
LB 3.00 Hz
GB 0
PC 1.40
210 200 190 180 170 160 150 140 130 120 110 100 90 80 70 60 50 40 30 20 10 0 ppm

(4)

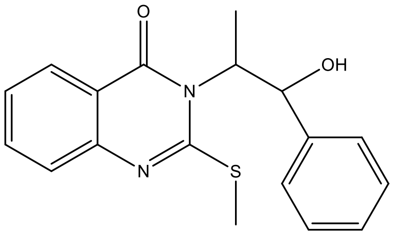

(5)

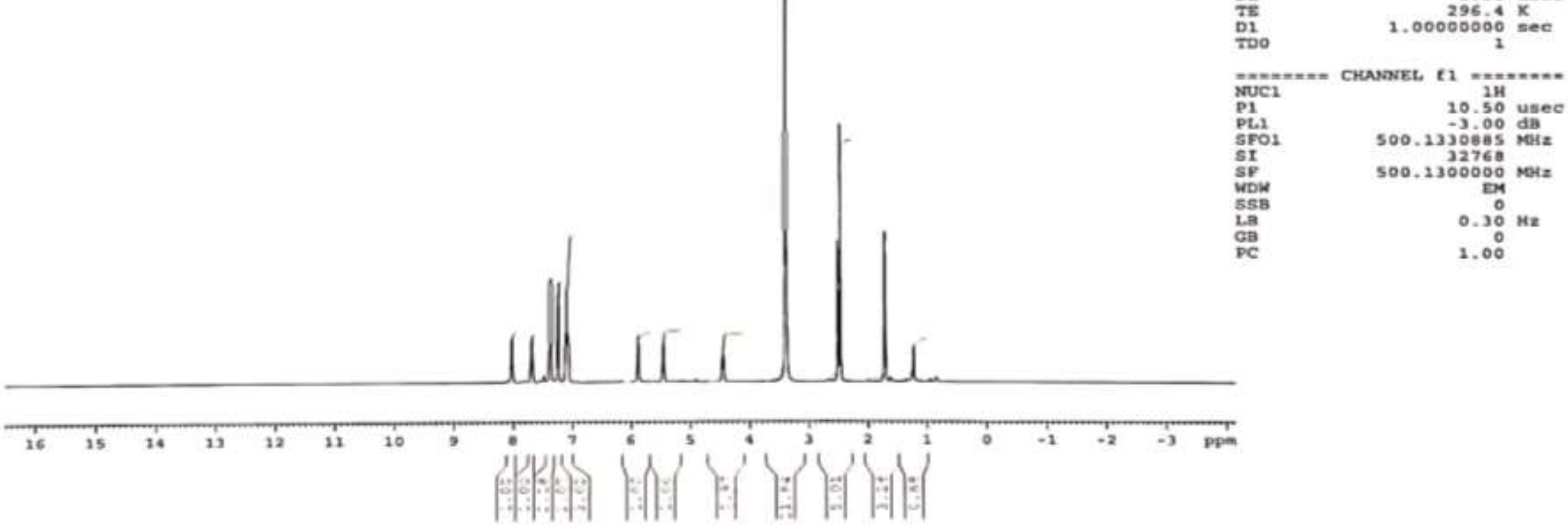
N-BBO DMSO D:\\ n
BRUKER
NAME drghorab-20
EXPNO 10
PROCNO 1
Date_ 20110306
Time 1.10
INSTRUM spect
PROBHD 5 mm BBO BB-1H
PULPROG zg30
TD 65536
SOLVENT DMSO
NS 16
DS 2
SWH 10330.578 Hz
FIDRES 0.157632 Hz
AQ 3.1720407 sec
RG 114
DW 48.400 usec
DE 6.50 usec
TE 296.4 K
D1 1.00000000 sec
TD0 1
======== CHANNEL f1 ========
NUC1 1H
P1 10.50 usec
PL1 -3.00 dB
SFO1 500.1330885 MHz
SI 32768
SF 500.1300000 MHz
WDW EM
SSB 0
LB 0.30 Hz
GB 0
PC 1.00
16 15 14 13 12 11 10 9 8 7 6 5 4 3 2 1 0 -1 -2 -3 ppm

N-BBO DMSO D:\\ n

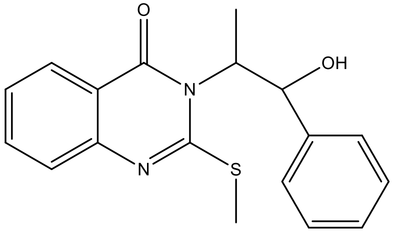

(5)

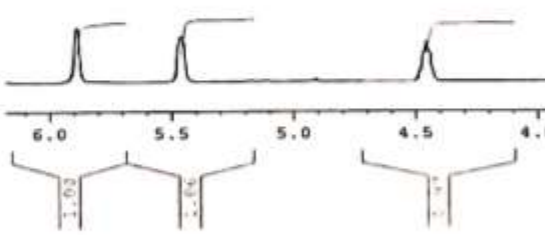
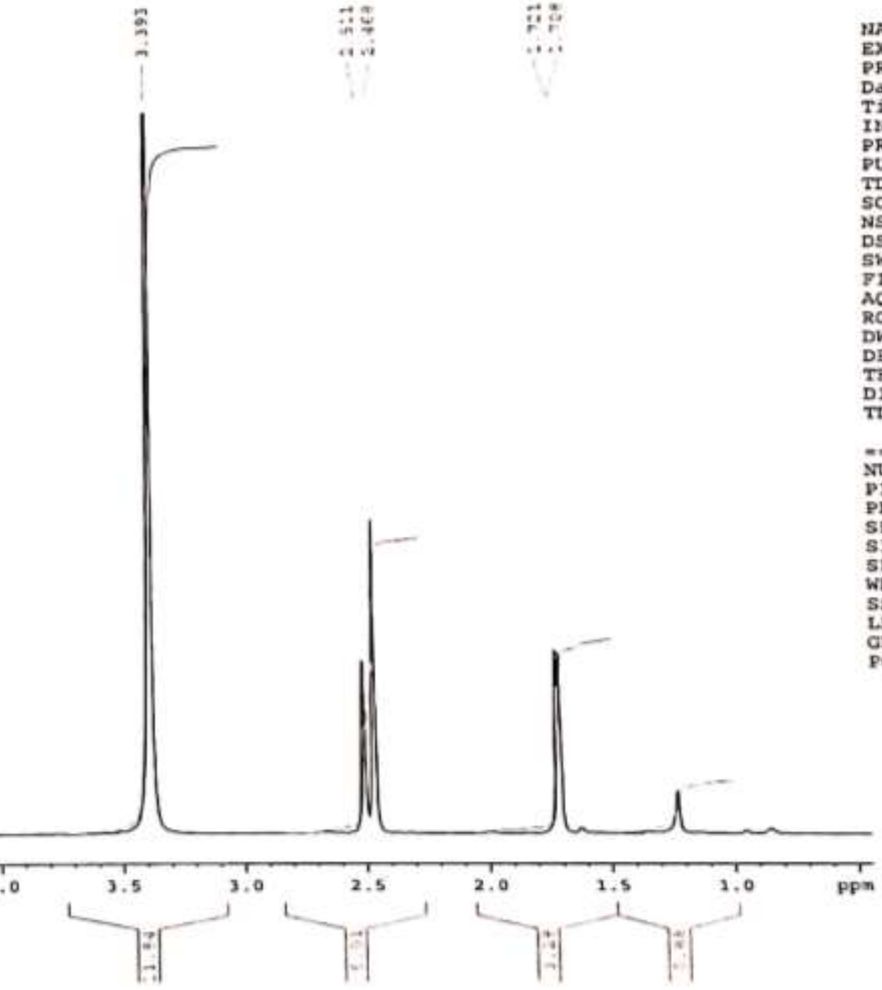

NAME	drghorab-20
EXPNO	10
PROCNO	1
Date_	20110306
Time	1.10
INSTRUM	spect
PROBHD	5 mm BBO BB-1H
PULPROG	zg30
TD	65536
SOLVENT	DMSO
NS	16
DS	2
SWH	10330.578 Hz
FIDRES	0.157632 Hz
AQ	3.1720407 sec
RG	114
DW	48.400 usec
DE	6.50 usec
TE	296.4 K
D1	1.00000000 sec
TD0	1
======== CHANNEL f1 ========	
NUC1	1H
P1	10.50 usec
PL1	-3.00 dB
SFO1	500.1330885 MHz
SI	32768
SF	500.1300000 MHz
WDW	EM
SSB	0
LB	0.30 Hz
GB	0
PC	1.00

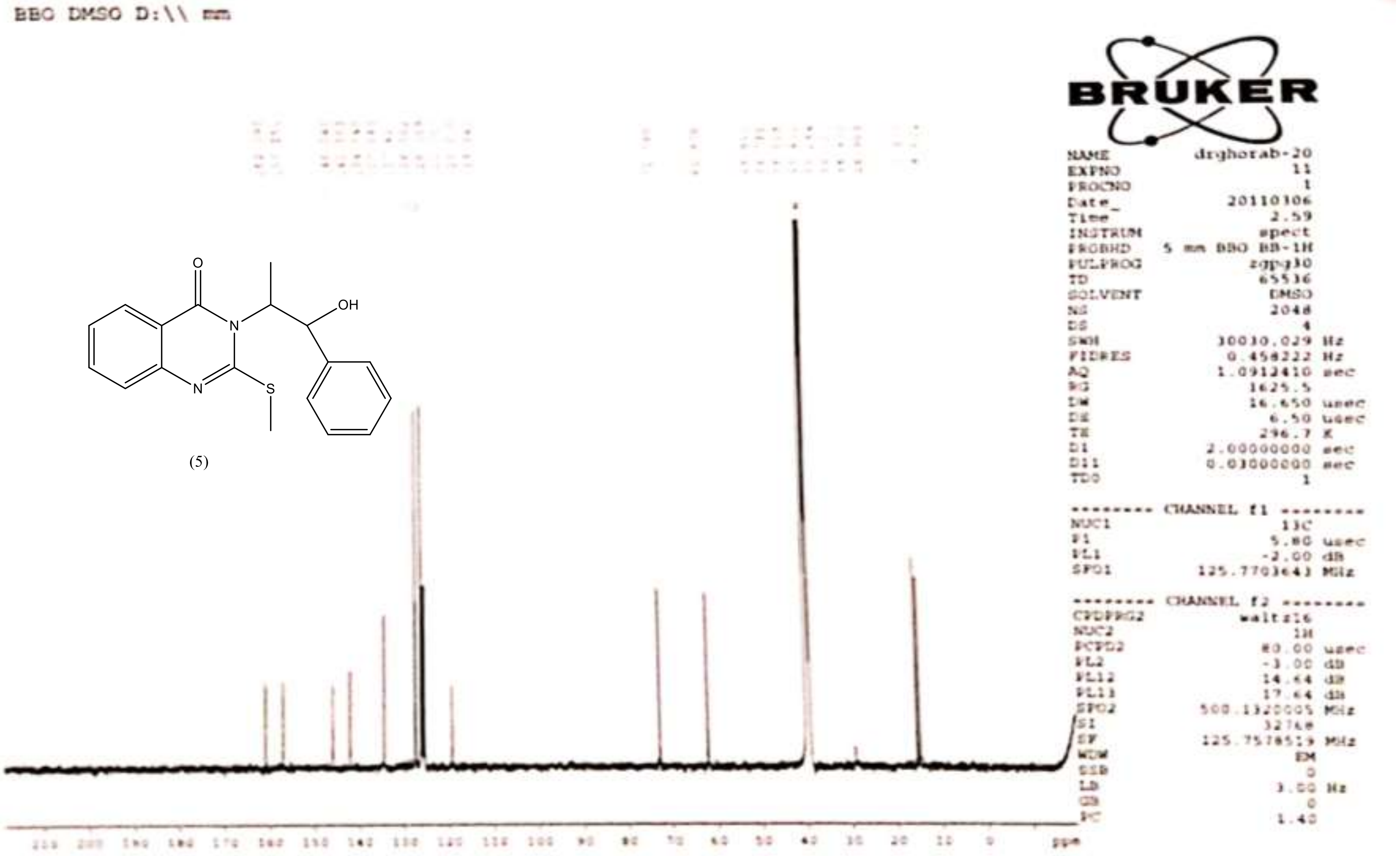

BBO DMSO D:\\ mm
(5)
BRUKER
NAME drghorab-20
EXPNO 11
PROCNO 1
Date_ 20110306
Time 2.59
INSTRUM spect
PROBHD 5 mm BBO BB-1H
PULPROG zgpg30
TD 65536
SOLVENT DMSO
NS 2048
DS 4
SWH 30030.029 Hz
FIDRES 0.458222 Hz
AQ 1.0912410 sec
RG 1625.5
DW 16.650 usec
DE 6.50 usec
TE 296.7 K
D1 2.00000000 sec
D11 0.03000000 sec
TD0 1
======== CHANNEL f1 ========
NUC1 13C
P1 5.80 usec
PL1 -2.00 dB
SFO1 125.7703643 MHz
======== CHANNEL f2 ========
CPDPRG2 waltz16
NUC2 1H
PCPD2 80.00 usec
PL2 -3.00 dB
PL12 14.64 dB
PL13 17.64 dB
SFO2 500.1320005 MHz
SI 32768
SF 125.7578519 MHz
WDW EM
SSB 0
LB 3.00 Hz
GB 0
PC 1.40
ppm

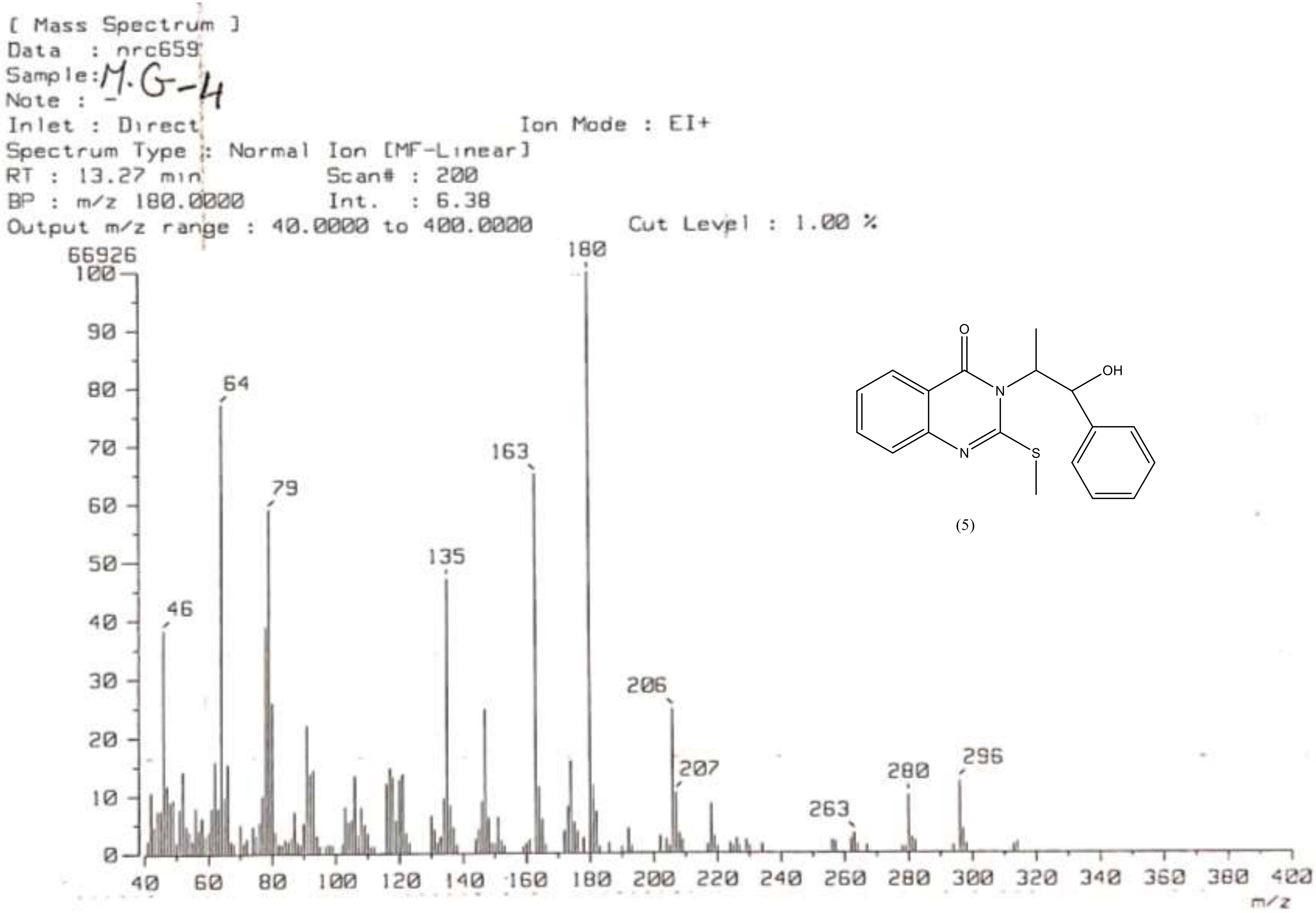
[Mass Spectrum]
Data : nrc659
Sample: M.G-4
Note : -
Inlet : Direct
Ion Mode : EI+
Spectrum Type : Normal Ion [MF-Linear]
RT : 13.27 min
Scan# : 200
BP : m/z 180.0000
Int. : 6.38
Output m/z range : 40.0000 to 400.0000
Cut Level : 1.00 %
66926
180
64
79
163
135
46
206
207
296
280
263
m/z
O
N
N
S
OH

(5)

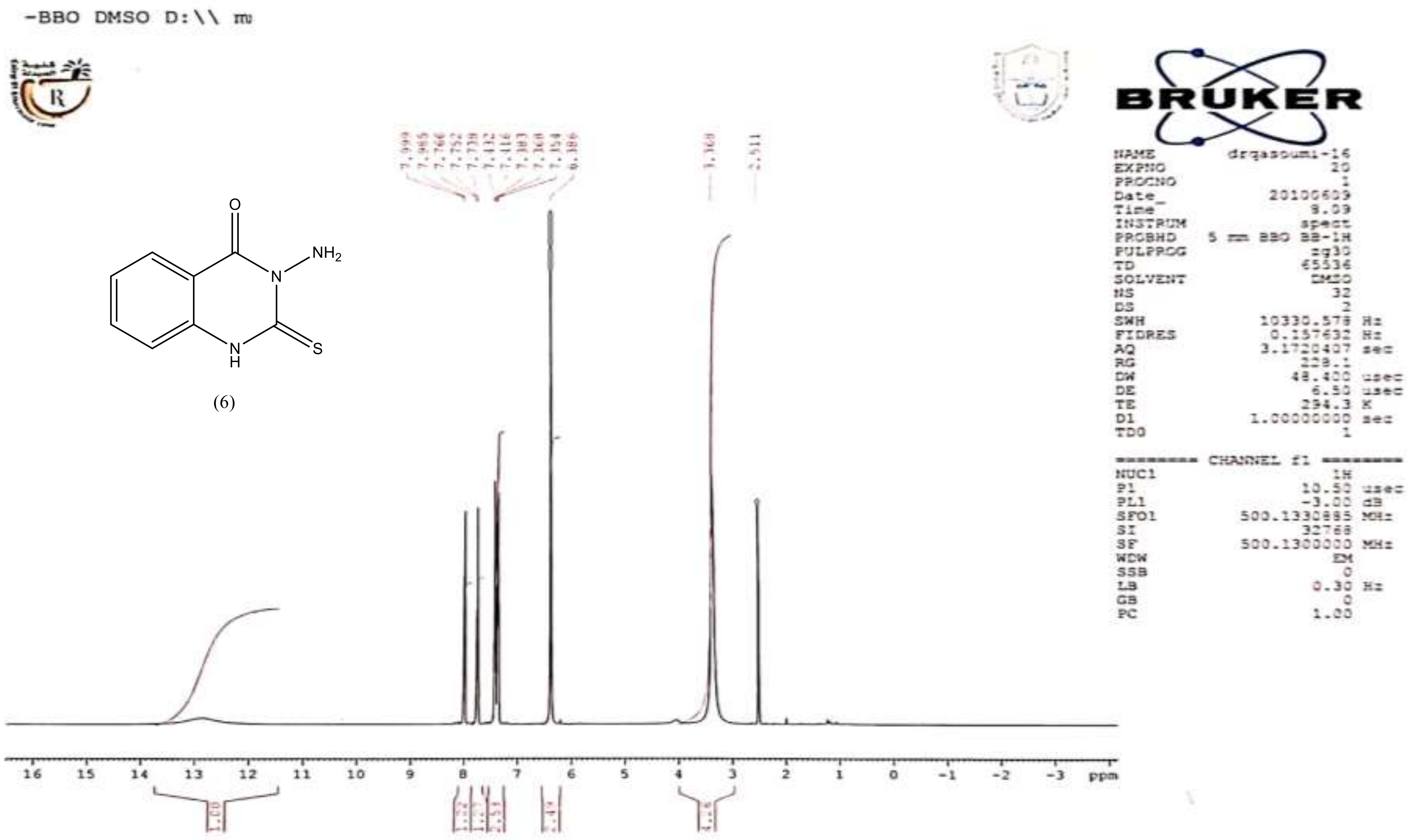
O
NH2
N
N
H
S
(6)
-BBO DMSO D:\\ m
BRUKER
NAME drqasoumi-16
EXPNO 20
PROCNO 1
Date_ 20100609
Time 9.09
INSTRUM spect
PROBHD 5 mm BBO BB-1H
PULPROG zg30
TD 65536
SOLVENT DMSO
NS 32
DS 2
SWH 10330.578 Hz
FIDRES 0.157632 Hz
AQ 3.1720407 sec
RG 228.1
DW 48.400 usec
DE 6.50 usec
TE 294.3 K
D1 1.00000000 sec
TD0 1
======== CHANNEL f1 ========
NUC1 1H
P1 10.50 usec
PL1 -3.00 dB
SFO1 500.1330885 MHz
SI 32768
SF 500.1300000 MHz
WDW EM
SSB 0
LB 0.30 Hz
GB 0
PC 1.00
ppm

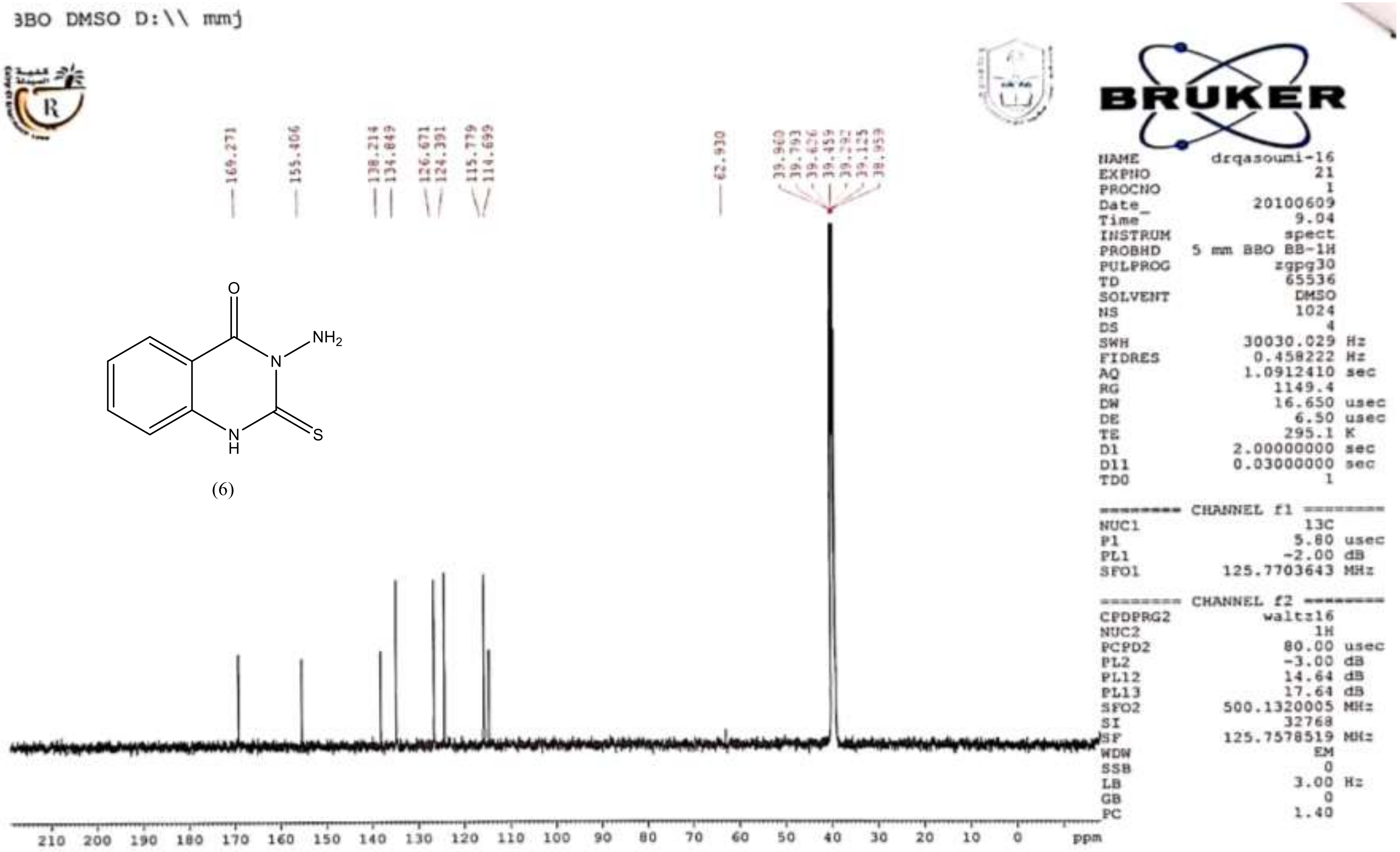
BBO DMSO D:\\ mmj
BRUKER
169.271
155.406
138.214
134.849
126.671
124.391
115.779
114.699
62.930
39.960
39.793
39.626
39.459
39.292
39.125
38.959
O
NH2
N
N
H
S
(6)
NAME drqasoumi-16
EXPNO 21
PROCNO 1
Date_ 20100609
Time 9.04
INSTRUM spect
PROBHD 5 mm BBO BB-1H
PULPROG zgpg30
TD 65536
SOLVENT DMSO
NS 1024
DS 4
SWH 30030.029 Hz
FIDRES 0.458222 Hz
AQ 1.0912410 sec
RG 1149.4
DW 16.650 usec
DE 6.50 usec
TE 295.1 K
D1 2.00000000 sec
D11 0.03000000 sec
TD0 1
======== CHANNEL f1 ========
NUC1 13C
P1 5.80 usec
PL1 -2.00 dB
SFO1 125.7703643 MHz
======== CHANNEL f2 ========
CPDPRG2 waltz16
NUC2 1H
PCPD2 80.00 usec
PL2 -3.00 dB
PL12 14.64 dB
PL13 17.64 dB
SFO2 500.1320005 MHz
SI 32768
SF 125.7578519 MHz
WDW EM
SSB 0
LB 3.00 Hz
GB 0
PC 1.40
210 200 190 180 170 160 150 140 130 120 110 100 90 80 70 60 50 40 30 20 10 0 ppm

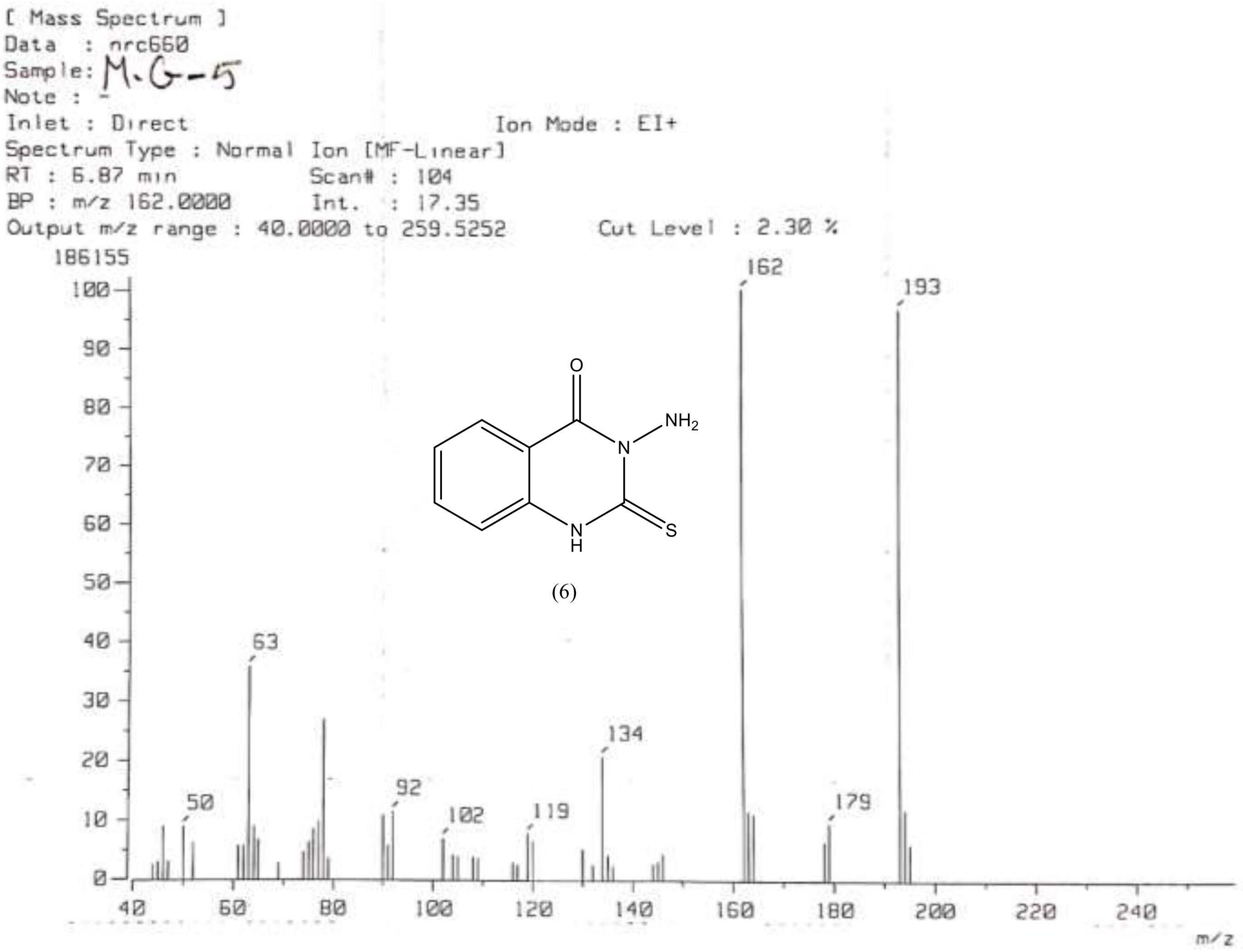
[Mass Spectrum]
Data : nrc660
Sample: M.G-5
Note : -
Inlet : Direct
Ion Mode : EI+
Spectrum Type : Normal Ion [MF-Linear]
RT : 6.87 min
Scan# : 104
BP : m/z 162.0000
Int. : 17.35
Output m/z range : 40.0000 to 259.5252
Cut Level : 2.30 %
186155
162
193
63
134
50
92
102
119
179
O
NH2
N
N
H
S
m/z

(6)

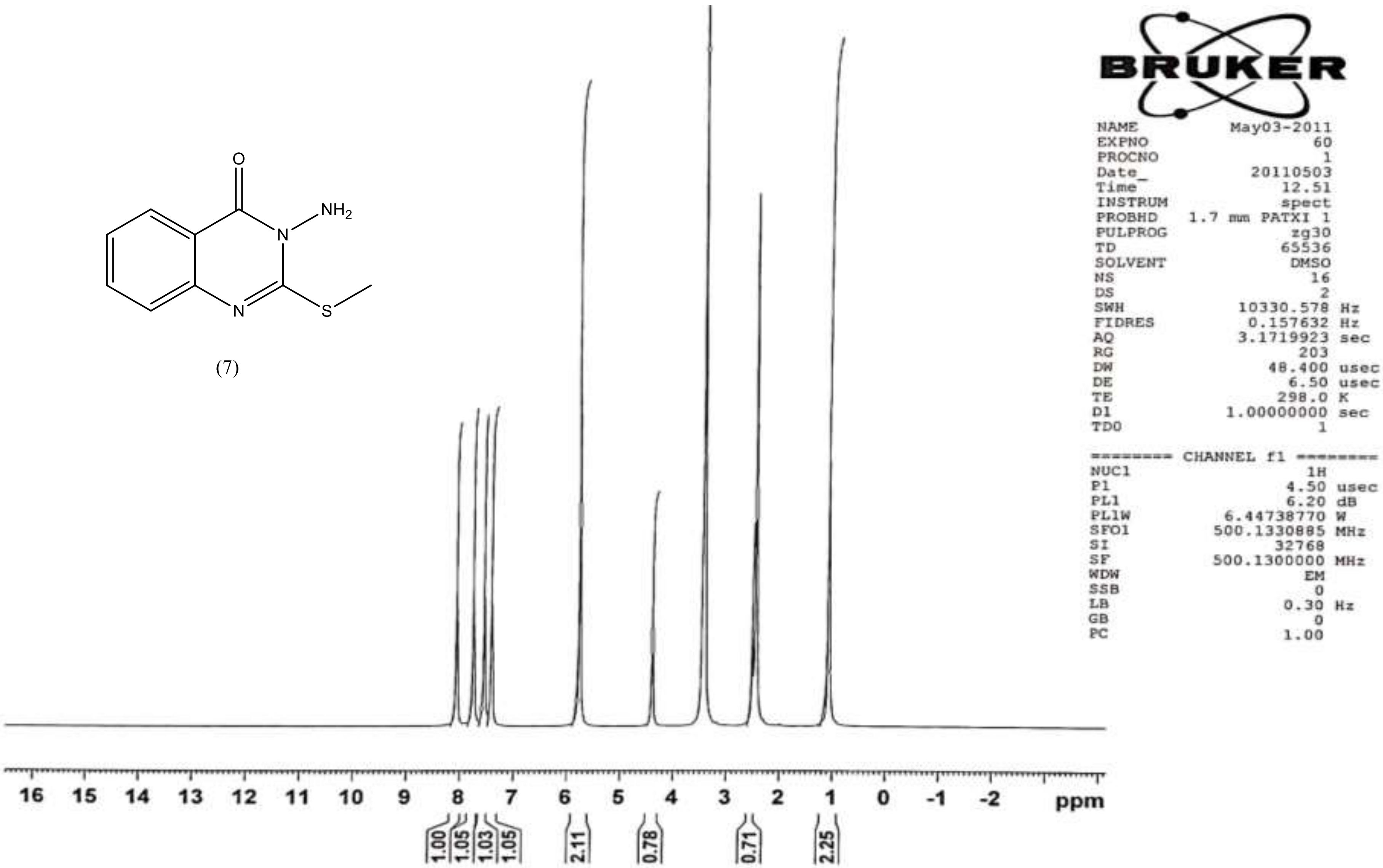
O
NH2
N
N
S
BRUKER
NAME May03-2011
EXPNO 60
PROCNO 1
Date_ 20110503
Time 12.51
INSTRUM spect
PROBHD 1.7 mm PATXI 1
PULPROG zg30
TD 65536
SOLVENT DMSO
NS 16
DS 2
SWH 10330.578 Hz
FIDRES 0.157632 Hz
AQ 3.1719923 sec
RG 203
DW 48.400 usec
DE 6.50 usec
TE 298.0 K
D1 1.00000000 sec
TD0 1
======== CHANNEL f1 ========
NUC1 1H
P1 4.50 usec
PL1 6.20 dB
PL1W 6.44738770 W
SFO1 500.1330885 MHz
SI 32768
SF 500.1300000 MHz
WDW EM
SSB 0
LB 0.30 Hz
GB 0
PC 1.00
16 15 14 13 12 11 10 9 8 7 6 5 4 3 2 1 0 -1 -2 ppm
1.00
1.05
1.03
1.05
2.11
0.78
0.71
2.25

(7)

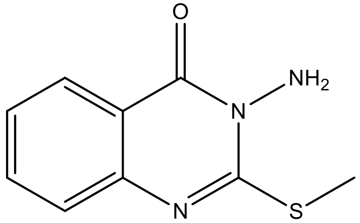
O
NH_2
N
N
S

(7)

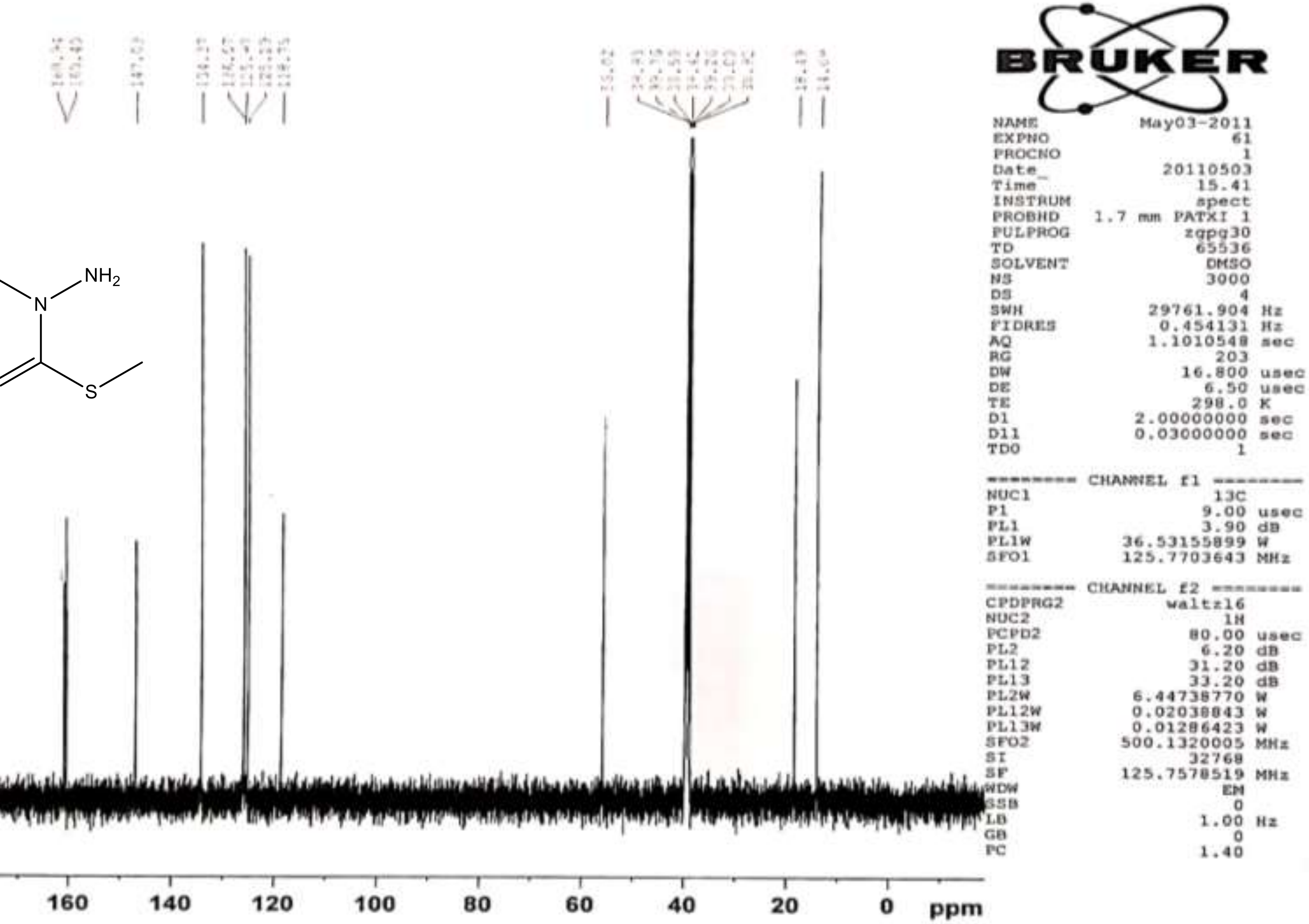
BRUKER
NAME May03-2011
EXPNO 61
PROCNO 1
Date_ 20110503
Time 15.41
INSTRUM spect
PROBHD 1.7 mm PATXI 1
PULPROG zgpg30
TD 65536
SOLVENT DMSO
NS 3000
DS 4
SWH 29761.904 Hz
FIDRES 0.454131 Hz
AQ 1.1010548 sec
RG 203
DW 16.800 usec
DE 6.50 usec
TE 298.0 K
D1 2.00000000 sec
D11 0.03000000 sec
TD0 1
======== CHANNEL f1 ========
NUC1 13C
P1 9.00 usec
PL1 3.90 dB
PL1W 36.53155899 W
SFO1 125.7703643 MHz
======== CHANNEL f2 ========
CPDPRG2 waltz16
NUC2 1H
PCPD2 80.00 usec
PL2 6.20 dB
PL12 31.20 dB
PL13 33.20 dB
PL2W 6.44738770 W
PL12W 0.02038843 W
PL13W 0.01286423 W
SFO2 500.1320005 MHz
SI 32768
SF 125.7578519 MHz
WDW EM
SSB 0
LB 1.00 Hz
GB 0
PC 1.40
200 180 160 140 120 100 80 60 40 20 0 ppm

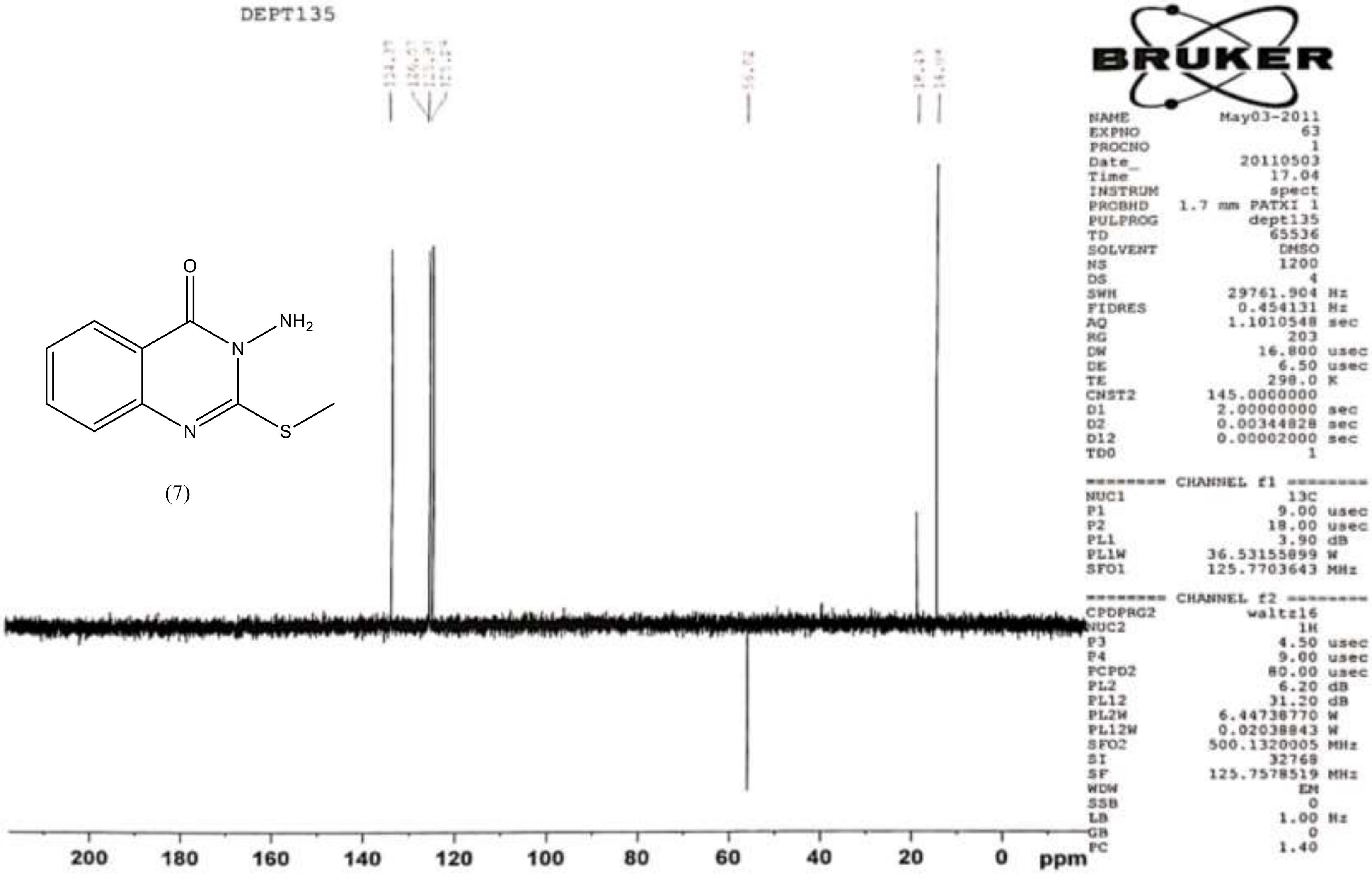
O
NH2
N
N
S
(7)
DEPT135
BRUKER
NAME May03-2011
EXPNO 63
PROCNO 1
Date_ 20110503
Time 17.04
INSTRUM spect
PROBHD 1.7 mm PATXI 1
PULPROG dept135
TD 65536
SOLVENT DMSO
NS 1200
DS 4
SWH 29761.904 Hz
FIDRES 0.454131 Hz
AQ 1.1010548 sec
RG 203
DW 16.800 usec
DE 6.50 usec
TE 298.0 K
CNST2 145.0000000
D1 2.00000000 sec
D2 0.00344828 sec
D12 0.00002000 sec
TD0 1
======== CHANNEL f1 ========
NUC1 13C
P1 9.00 usec
P2 18.00 usec
PL1 3.90 dB
PL1W 36.53155899 W
SFO1 125.7703643 MHz
======== CHANNEL f2 ========
CPDPRG2 waltz16
NUC2 1H
P3 4.50 usec
P4 9.00 usec
PCPD2 80.00 usec
PL2 6.20 dB
PL12 31.20 dB
PL2W 6.44738770 W
PL12W 0.02038843 W
SFO2 500.1320005 MHz
SI 32768
SF 125.7578519 MHz
WDW EM
SSB 0
LB 1.00 Hz
GB 0
PC 1.40
200 180 160 140 120 100 80 60 40 20 0 ppm

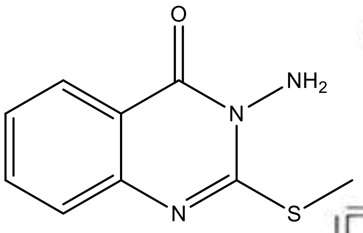
O
NH2
N
N
S

(7)

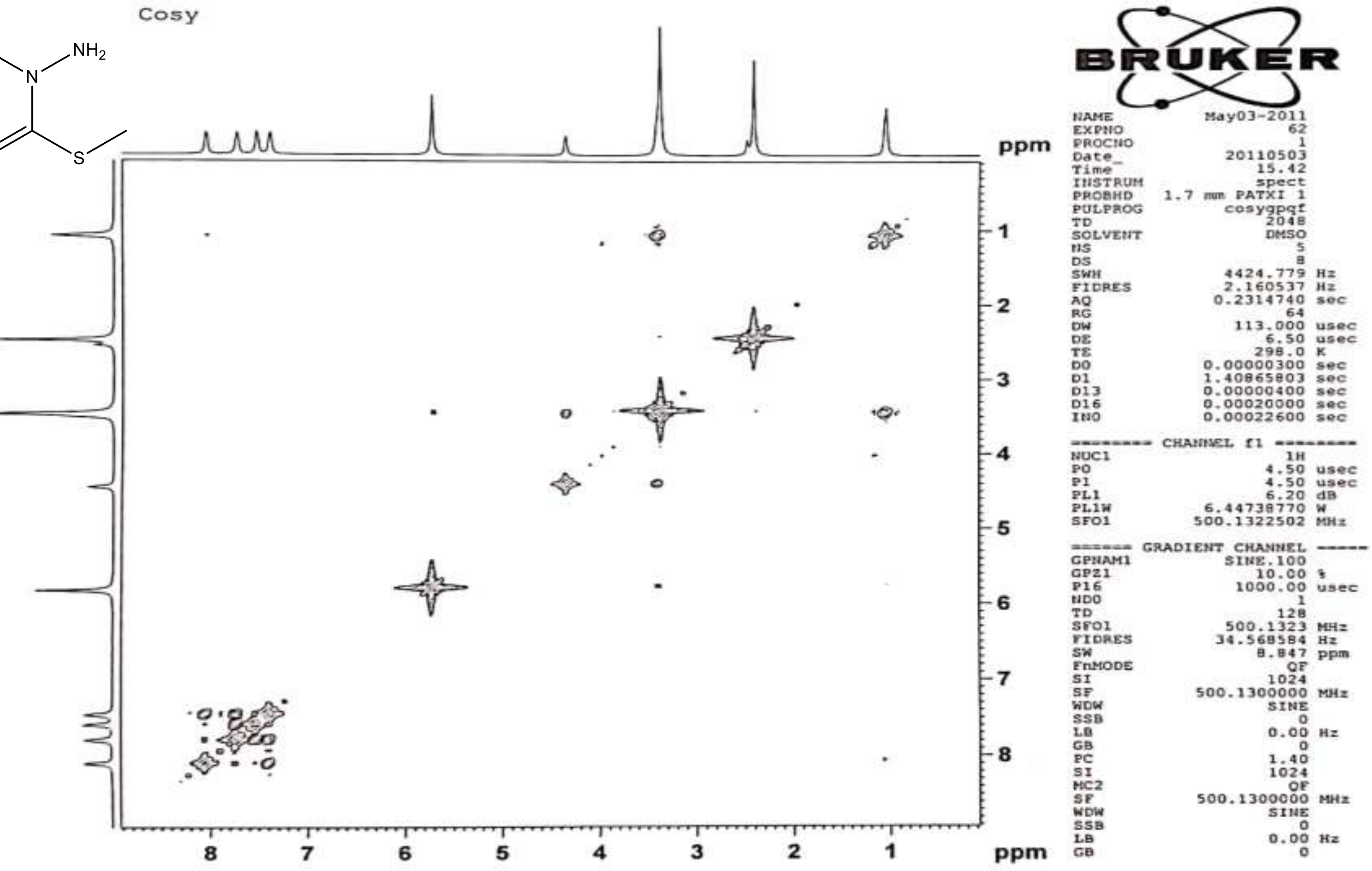
Cosy
ppm
ppm
BRUKER
NAME May03-2011
EXPNO 62
PROCNO 1
Date_ 20110503
Time 15.42
INSTRUM spect
PROBHD 1.7 mm PATXI 1
PULPROG cosygpqf
TD 2048
SOLVENT DMSO
NS 5
DS 8
SWH 4424.779 Hz
FIDRES 2.160537 Hz
AQ 0.2314740 sec
RG 64
DW 113.000 usec
DE 6.50 usec
TE 298.0 K
D0 0.00000300 sec
D1 1.40865803 sec
D13 0.00000400 sec
D16 0.00020000 sec
IN0 0.00022600 sec
======== CHANNEL f1 ========
NUC1 1H
P0 4.50 usec
P1 4.50 usec
PL1 6.20 dB
PL1W 6.44738770 W
SFO1 500.1322502 MHz
====== GRADIENT CHANNEL =====
GPNAM1 SINE.100
GPZ1 10.00 %
P16 1000.00 usec
ND0 1
TD 128
SFO1 500.1323 MHz
FIDRES 34.568584 Hz
SW 8.847 ppm
FnMODE QF
SI 1024
SF 500.1300000 MHz
WDW SINE
SSB 0
LB 0.00 Hz
GB 0
PC 1.40
SI 1024
MC2 QF
SF 500.1300000 MHz
WDW SINE
SSB 0
LB 0.00 Hz
GB 0

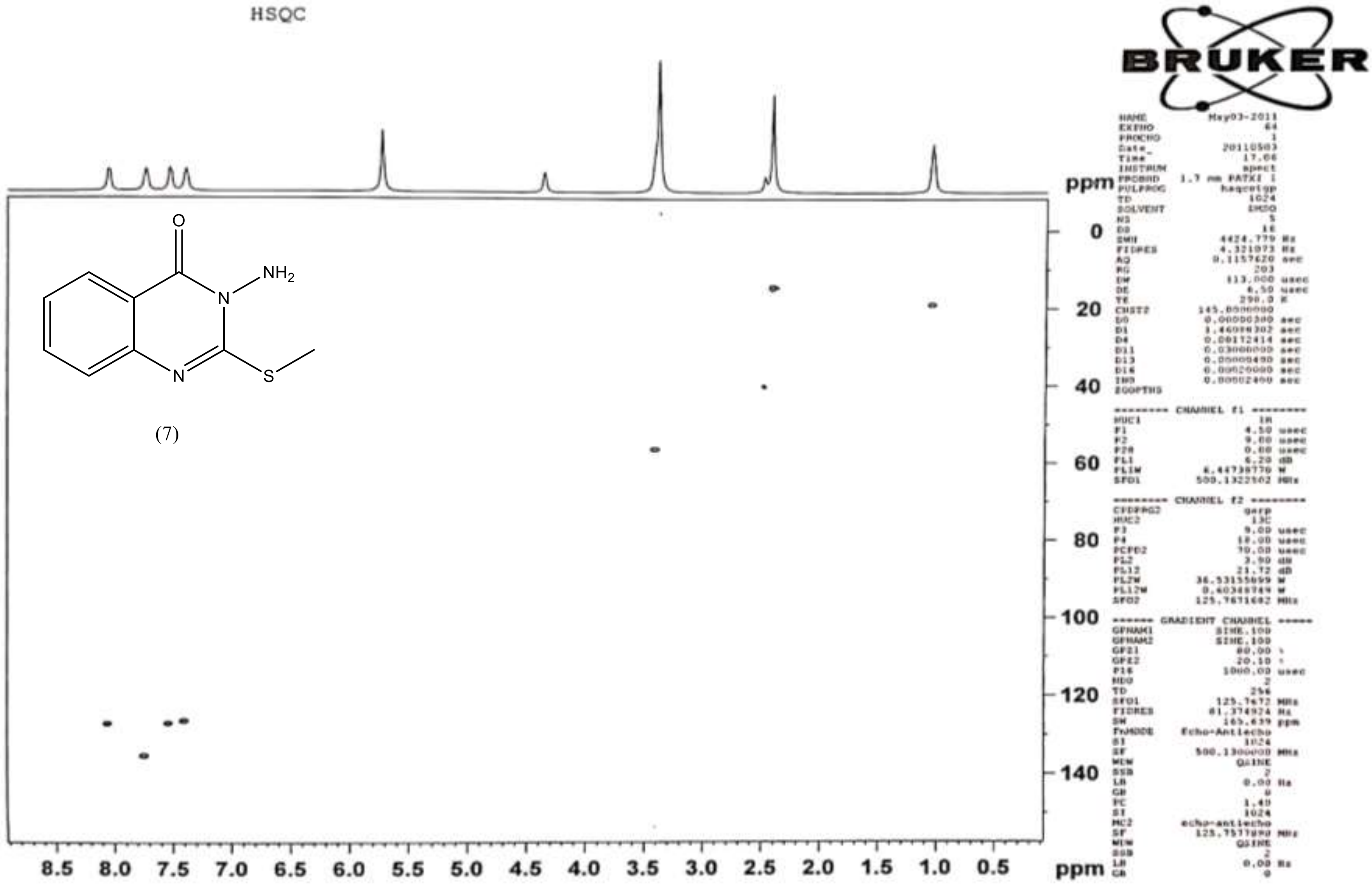
HSQC
O
N
NH2
N
S
(7)
ppm
0
20
40
60
80
100
120
140
8.5 8.0 7.5 7.0 6.5 6.0 5.5 5.0 4.5 4.0 3.5 3.0 2.5 2.0 1.5 1.0 0.5
ppm

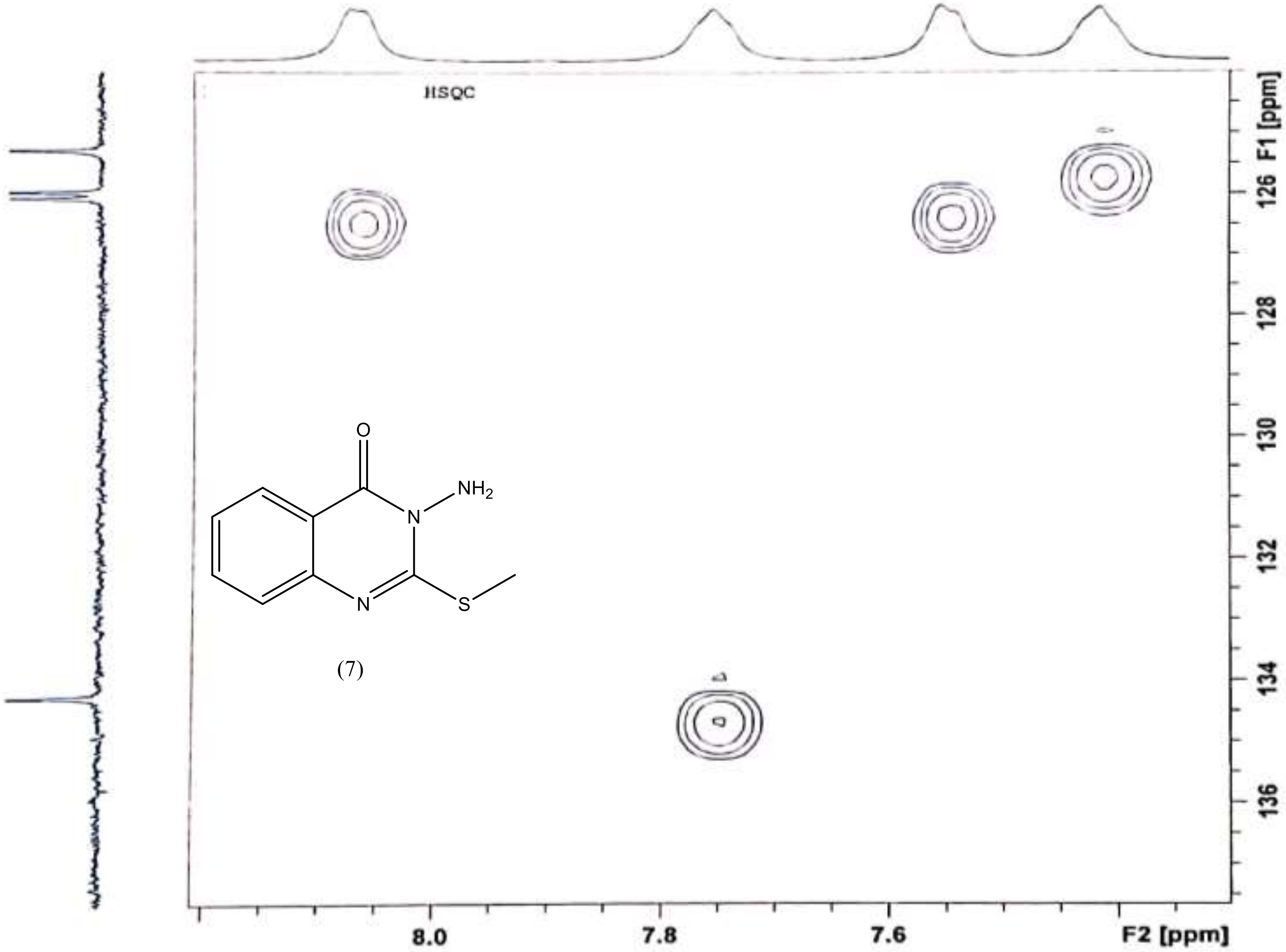
HSQC
F1 [ppm]
126
128
130
132
134
136
F2 [ppm]
8.0
7.8
7.6
O
NH_2
N
N
S
(7)

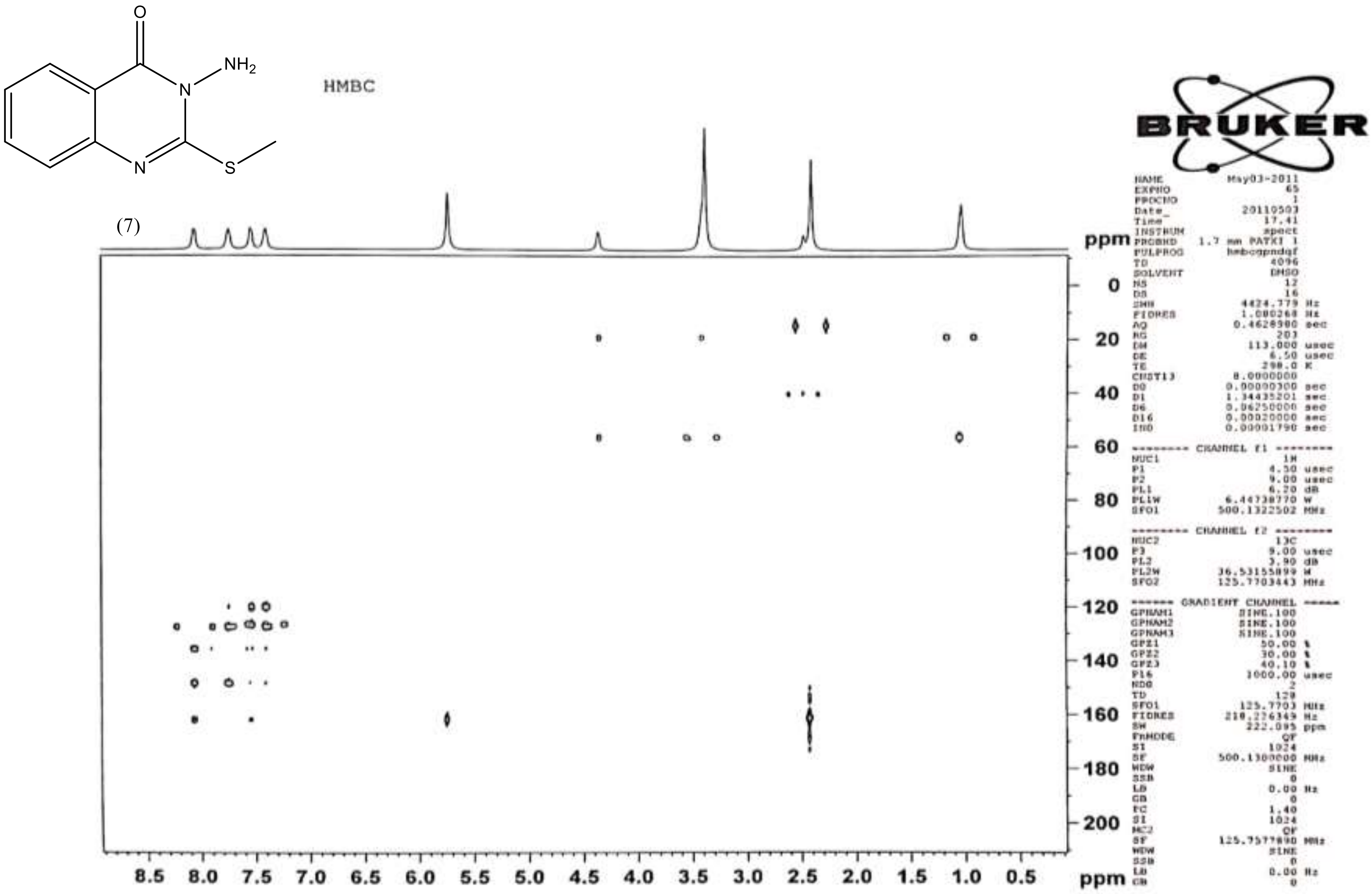
O
NH2
N
N
S
(7)
HMBC
BRUKER
NAME May03-2011
EXPNO 65
PROCNO 1
Date_ 20110503
Time 17.41
INSTRUM spect
PROBHD 1.7 mm PATXI 1
PULPROG hmbcgpndqf
TD 4096
SOLVENT DMSO
NS 12
DS 16
SWH 4424.779 Hz
FIDRES 1.080268 Hz
AQ 0.4628980 sec
RG 203
DW 113.000 usec
DE 6.50 usec
TE 298.0 K
CNST13 8.0000000
D0 0.00000300 sec
D1 1.34435201 sec
D6 0.06250000 sec
D16 0.00020000 sec
IN0 0.00001790 sec
======== CHANNEL f1 ========
NUC1 1H
P1 4.50 usec
P2 9.00 usec
PL1 6.20 dB
PL1W 6.44738770 W
SFO1 500.1322502 MHz
======== CHANNEL f2 ========
NUC2 13C
P3 9.00 usec
PL2 3.90 dB
PL2W 36.53155899 W
SFO2 125.7703443 MHz
====== GRADIENT CHANNEL =====
GPNAM1 SINE.100
GPNAM2 SINE.100
GPNAM3 SINE.100
GPZ1 50.00 %
GPZ2 30.00 %
GPZ3 40.10 %
P16 1000.00 usec
ND0 2
TD 128
SFO1 125.7703 MHz
FIDRES 218.226349 Hz
SW 222.095 ppm
FnMODE QF
SI 1024
SF 500.1300000 MHz
WDW SINE
SSB 0
LB 0.00 Hz
GB 0
PC 1.40
SI 1024
MC2 QF
SF 125.7577890 MHz
WDW SINE
SSB 0
LB 0.00 Hz
GB 0
ppm
0
20
40
60
80
100
120
140
160
180
200
8.5 8.0 7.5 7.0 6.5 6.0 5.5 5.0 4.5 4.0 3.5 3.0 2.5 2.0 1.5 1.0 0.5
ppm

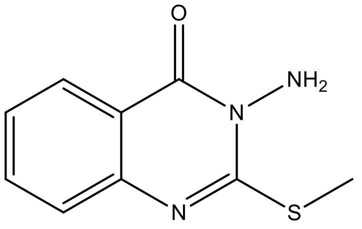

(7)

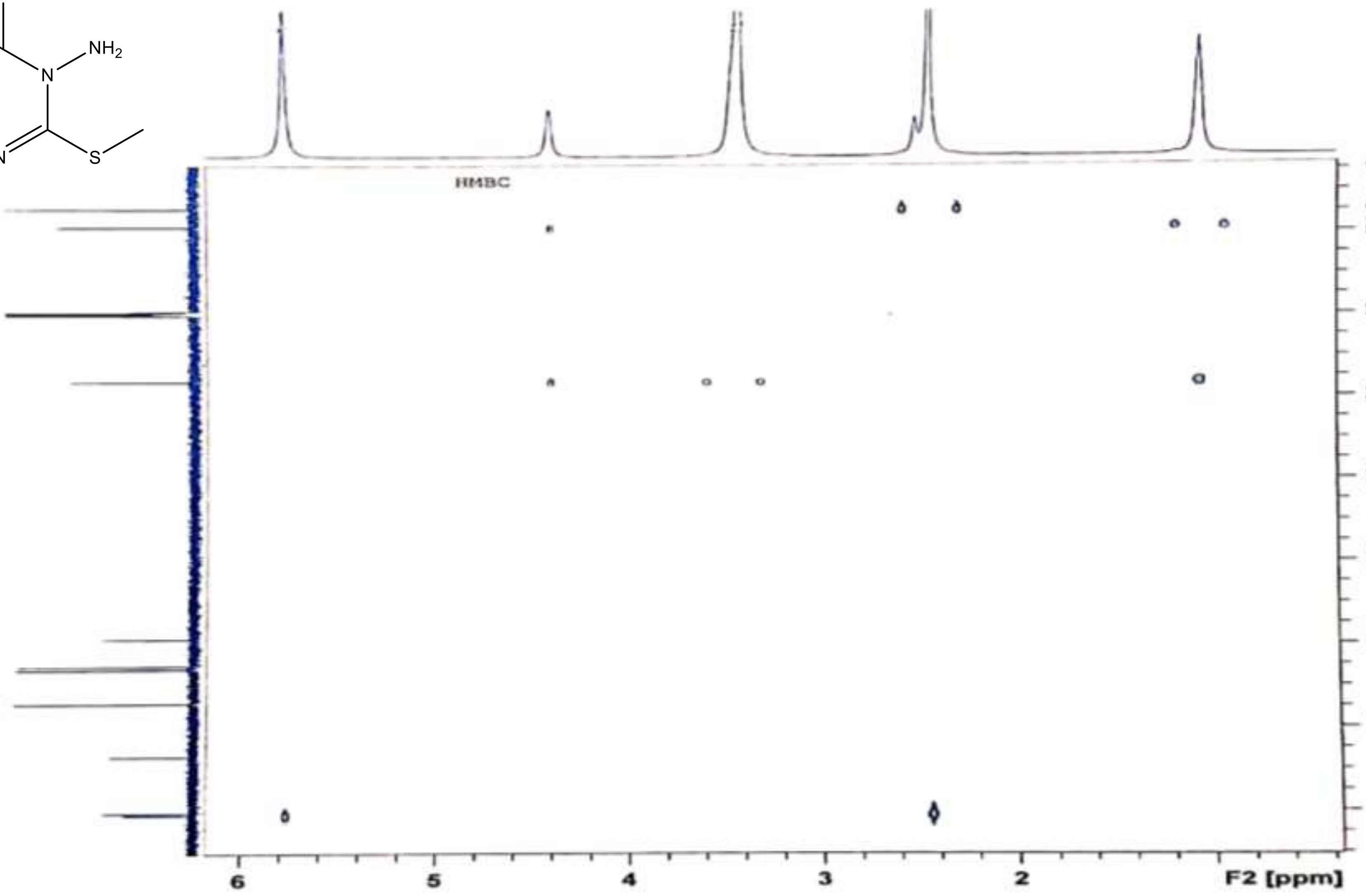

(8)

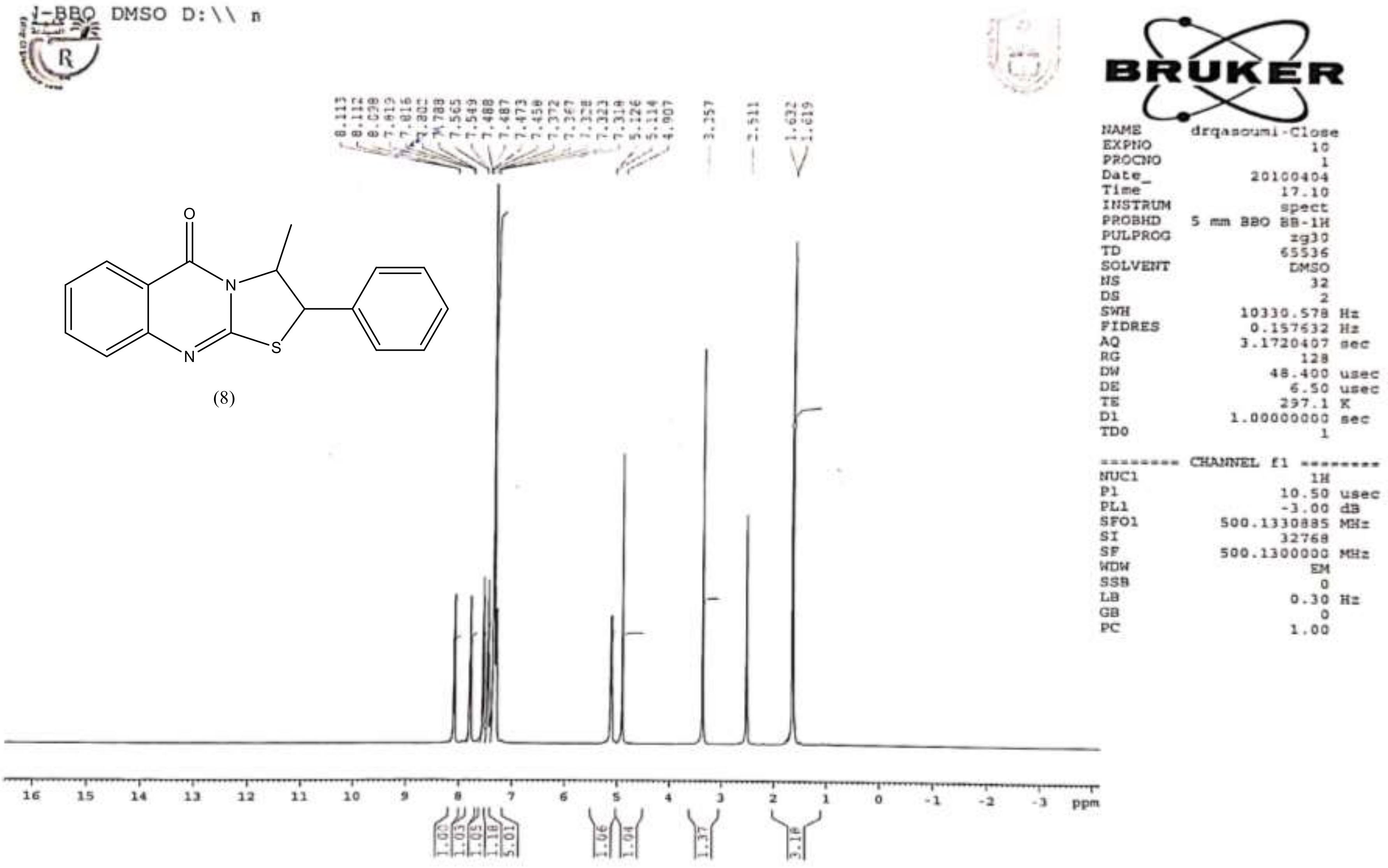
DMSO D:\\ n
BRUKER
NAME drqasoumi-Close
EXPNO 10
PROCNO 1
Date_ 20100404
Time 17.10
INSTRUM spect
PROBHD 5 mm BBO BB-1H
PULPROG zg30
TD 65536
SOLVENT DMSO
NS 32
DS 2
SWH 10330.578 Hz
FIDRES 0.157632 Hz
AQ 3.1720407 sec
RG 128
DW 48.400 usec
DE 6.50 usec
TE 297.1 K
D1 1.00000000 sec
TD0 1
======== CHANNEL f1 ========
NUC1 1H
P1 10.50 usec
PL1 -3.00 dB
SFO1 500.1330885 MHz
SI 32768
SF 500.1300000 MHz
WDW EM
SSB 0
LB 0.30 Hz
GB 0
PC 1.00
ppm

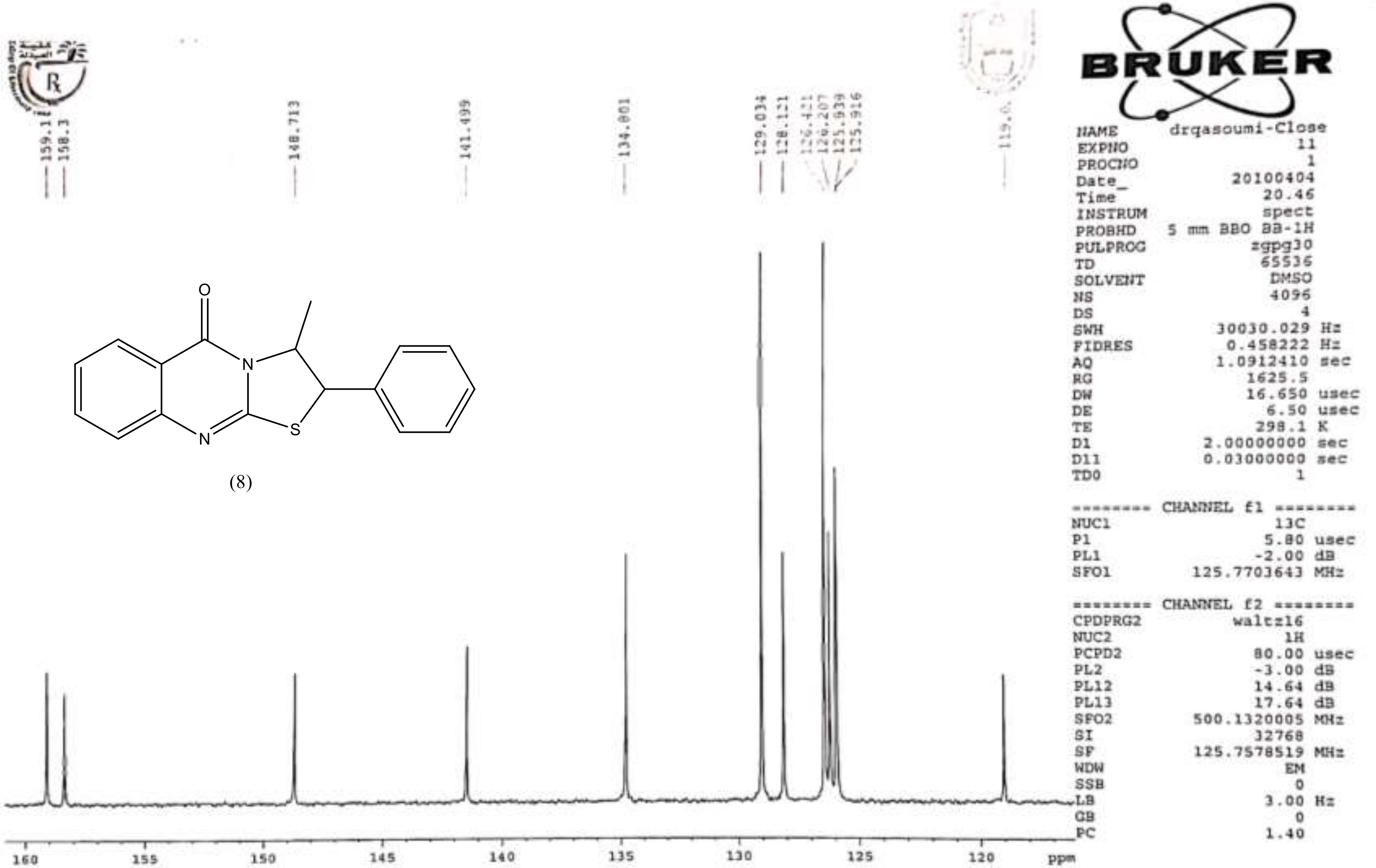
159.1
158.3
148.713
141.499
134.801
129.034
128.121
126.421
126.207
125.916
O
N
S
(8)
BRUKER
NAME drqasoumi-Close
EXPNO 11
PROCNO 1
Date_ 20100404
Time 20.46
INSTRUM spect
PROBHD 5 mm BBO BB-1H
PULPROG zgpg30
TD 65536
SOLVENT DMSO
NS 4096
DS 4
SWH 30030.029 Hz
FIDRES 0.458222 Hz
AQ 1.0912410 sec
RG 1625.5
DW 16.650 usec
DE 6.50 usec
TE 298.1 K
D1 2.00000000 sec
D11 0.03000000 sec
TD0 1
======== CHANNEL f1 ========
NUC1 13C
P1 5.80 usec
PL1 -2.00 dB
SFO1 125.7703643 MHz
======== CHANNEL f2 ========
CPDPRG2 waltz16
NUC2 1H
PCPD2 80.00 usec
PL2 -3.00 dB
PL12 14.64 dB
PL13 17.64 dB
SFO2 500.1320005 MHz
SI 32768
SF 125.7578519 MHz
WDW EM
SSB 0
LB 3.00 Hz
GB 0
PC 1.40
160
155
150
145
140
135
130
125
120
ppm

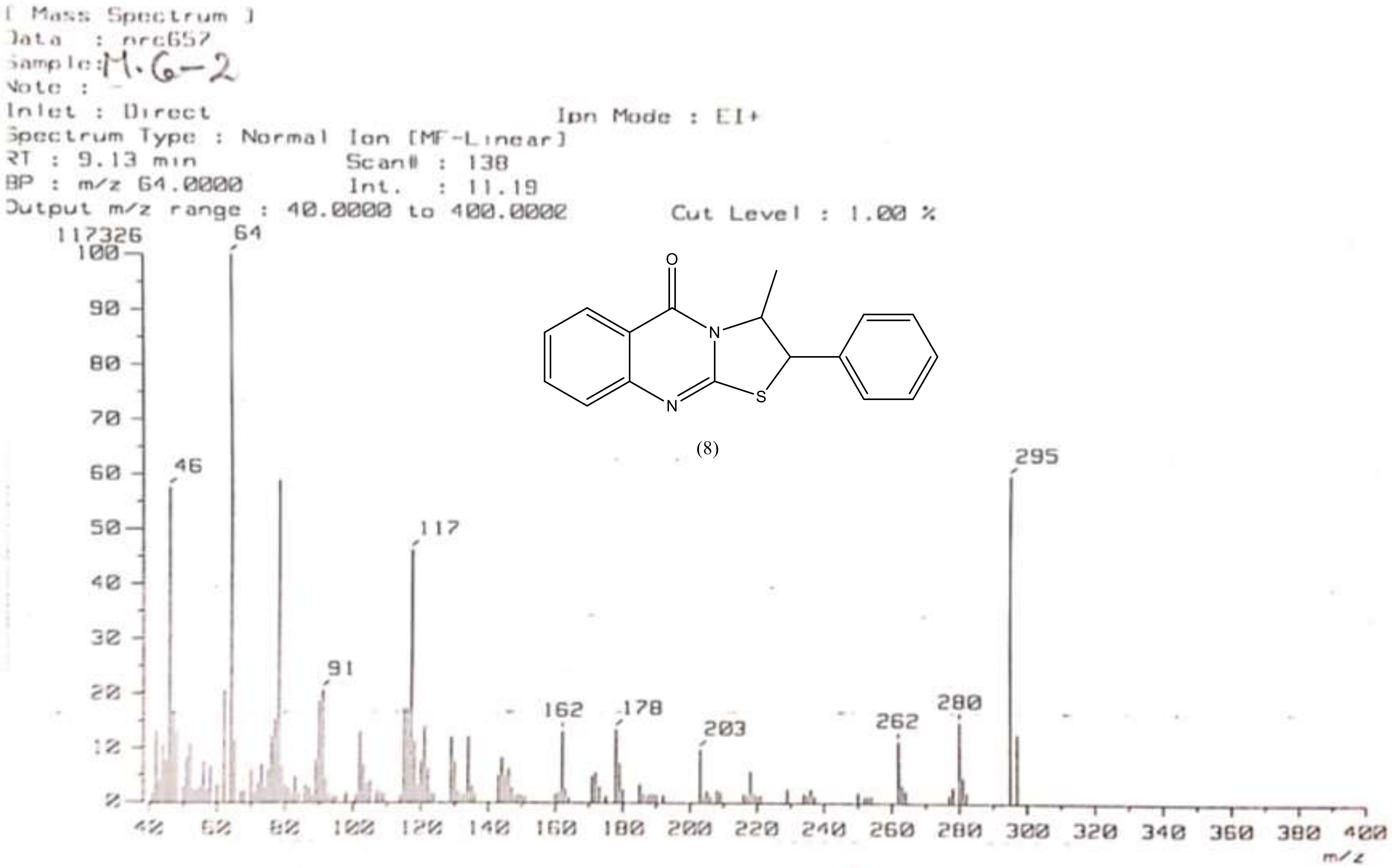
[Mass Spectrum]
Data : nrc657
Sample: M.6-2
Note : -
Inlet : Direct
Ion Mode : EI+
Spectrum Type : Normal Ion [MF-Linear]
RT : 9.13 min
Scan# : 138
BP : m/z 64.0000
Int. : 11.19
Output m/z range : 40.0000 to 400.0000
Cut Level : 1.00 %
117326
O
N
N
S
(8)
64
46
91
117
162
178
203
262
280
295
m/z
